# “Like sugar in milk”: reconstructing the genetic history of the Parsi population

**DOI:** 10.1186/s13059-017-1244-9

**Published:** 2017-06-14

**Authors:** Gyaneshwer Chaubey, Qasim Ayub, Niraj Rai, Satya Prakash, Veena Mushrif-Tripathy, Massimo Mezzavilla, Ajai Kumar Pathak, Rakesh Tamang, Sadaf Firasat, Maere Reidla, Monika Karmin, Deepa Selvi Rani, Alla G. Reddy, Jüri Parik, Ene Metspalu, Siiri Rootsi, Kurush Dalal, Shagufta Khaliq, Syed Qasim Mehdi, Lalji Singh, Mait Metspalu, Toomas Kivisild, Chris Tyler-Smith, Richard Villems, Kumarasamy Thangaraj

**Affiliations:** 1 Evolutionary Biology Group, Estonian Biocentre, Riia23b, Tartu, 51010 Estonia; 2 The Wellcome Trust Sanger Institute, Wellcome Genome Campus, Hinxton, Cambridgeshire, CB10 1SA UK; 3 CSIR - Centre for Cellular and Molecular Biology, Hyderabad, 500007 India; 4 Present address: Birbal Sahni Institute of Palaeosciences, Lucknow, 226007 India; 5 Department of Archaeology, Deccan College Post-Graduate and Research Institute, Pune, Maharashtra 411006 India; 6 Department of Evolutionary Biology, Institute of Molecular and Cell Biology, University of Tartu, Tartu, 51010 Estonia; 7 Department of Zoology, University of Calcutta, Kolkata, 700073 India; 8 Centre for Human Genetics and Molecular Medicine, Sindh Institute of Urology and Transplantation, Karachi, 74200 Pakistan; 9 Department of Psychology, University of Auckland, Auckland, 1142 New Zealand; 10 Centre for Archaeology (CfA), Centre for Extra Mural Studies (CEMS) University of Mumbai (Kalina Campus) Vidyanagri, Santacruz E Mumbai, 400098 India; 11 Department of Human Genetics & Molecular Biology, University of Health Sciences, Lahore, 54000 Pakistan; 12 Genome foundation, C/o Prasad Hospital, Nacharam, Hyderabad, 500076 India; 13 Division of Biological Anthropology, University of Cambridge, Cambridge, CB2 3QG UK

**Keywords:** Parsi, Zoroastrian, autosomes, mtDNA, Y chromosome, ancient DNA

## Abstract

**Background:**

The Parsis are one of the smallest religious communities in the world. To understand the population structure and demographic history of this group in detail, we analyzed Indian and Pakistani Parsi populations using high-resolution genetic variation data on autosomal and uniparental loci (Y-chromosomal and mitochondrial DNA). Additionally, we also assayed mitochondrial DNA polymorphisms among ancient Parsi DNA samples excavated from Sanjan, in present day Gujarat, the place of their original settlement in India.

**Results:**

Among present-day populations, the Parsis are genetically closest to Iranian and the Caucasus populations rather than their South Asian neighbors. They also share the highest number of haplotypes with present-day Iranians and we estimate that the admixture of the Parsis with Indian populations occurred ~1,200 years ago. Enriched homozygosity in the Parsi reflects their recent isolation and inbreeding. We also observed 48% South-Asian-specific mitochondrial lineages among the ancient samples, which might have resulted from the assimilation of local females during the initial settlement. Finally, we show that Parsis are genetically closer to Neolithic Iranians than to modern Iranians, who have witnessed a more recent wave of admixture from the Near East.

**Conclusions:**

Our results are consistent with the historically-recorded migration of the Parsi populations to South Asia in the 7th century and in agreement with their assimilation into the Indian sub-continent's population and cultural milieu "like sugar in milk". Moreover, in a wider context our results support a major demographic transition in West Asia due to the Islamic conquest.

**Electronic supplementary material:**

The online version of this article (doi:10.1186/s13059-017-1244-9) contains supplementary material, which is available to authorized users.

## Background

**Fig. 1:**
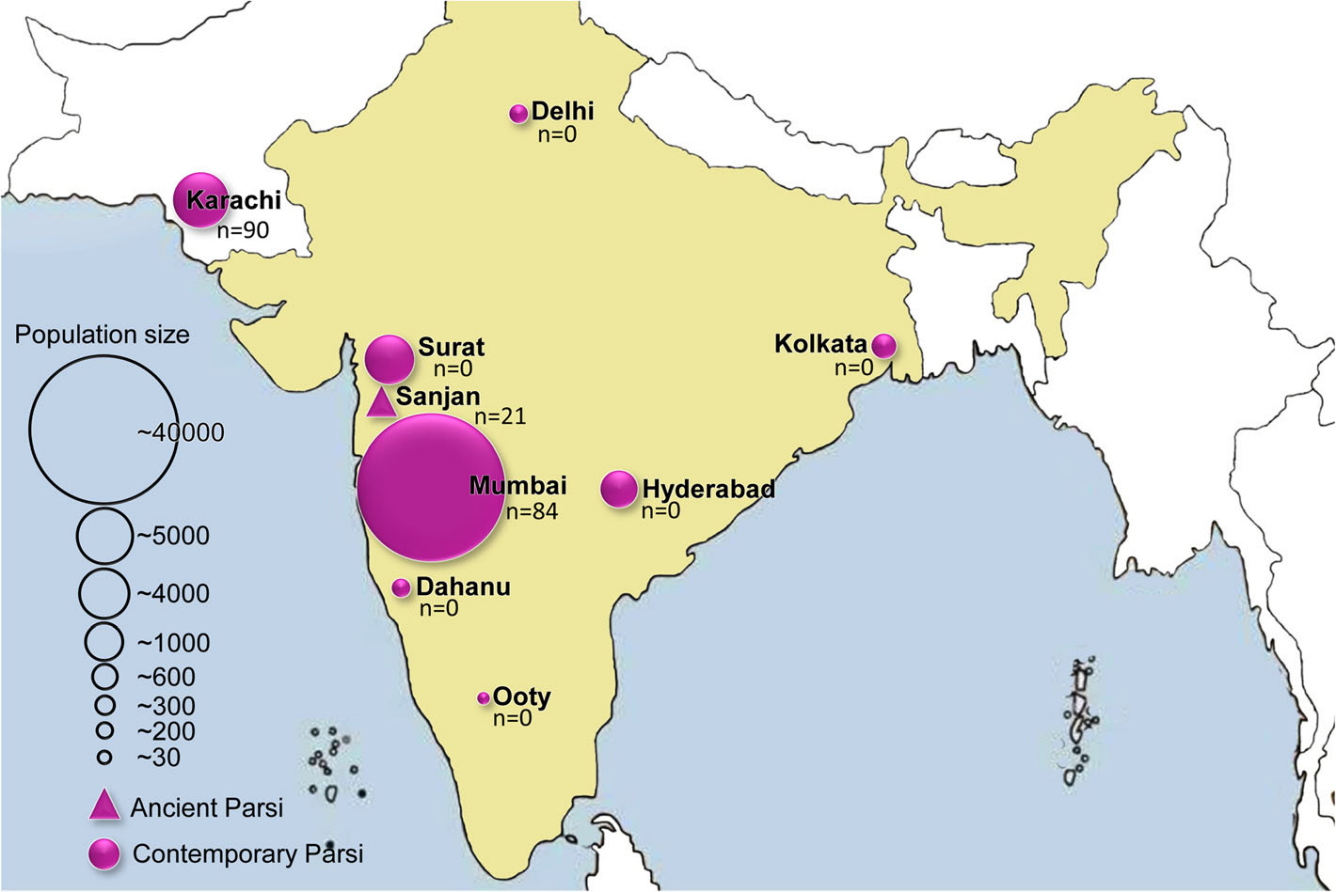
The geographical distribution and sampling locations of modern and ancient Parsi samples. The population data are obtained from the Parzor Foundation, New Delhi, India (http://unescoparzor.com/)

The Parsi (or Parsee) community of the Indian sub-continent are a group of Indo-European speakers and adherents of the Zoroastrian faith, one of the earliest monotheisms that flourished in pre-Islamic Persia (present-day Iran) [1, 2]. Zoroastrianism was the religion of Persia from 600 B.C. to 650 A.D. [3–5] and, despite a long history of well-preserved culture, it now has a limited number of followers [6, 7]. The Parzor Foundation reports the total number of Zoroastrians to be around 137,000, with 69,000 living in India, roughly 20,000 in Iran [8] and 2000–5000 in Pakistan [8, 9] (Fig. 1). This reduction in population is mainly due to strict marriage practices and low birth rates [7, 10–12].

The Parsi trace their ancestry to the ancestors of the Zoroastrians of modern Iran, who are followers of the Prophet Zoroaster or Zarathushtra [3]. In the 7th century, the Zoroastrian Sassanian dynasty was threatened by Islamic conquest and a small group of Zoroastrians fled to Gujarat in present-day India, where they were called ‘Parsi’ (literally meaning ‘people from Paras or Fars’, the local term for Persia) [3, 4, 6, 13]. Several myths narrate their first arrival in the West Coast of India and settlement in Sanjan (Gujarat) [4, 14–17]. The most popular one mentioned in the *Qissa-e-Sanjaan* is that an Indian ruler called *Jadi Rana* sent a glass full of milk to the Parsi group seeking asylum [4, 18]. His message was that his kingdom was full with local people. The Zoroastrian immigrants put sugar (or a ring, in some versions of the story) into the milk to indicate an assimilation of their people into the local society, like “sugar in milk” [14, 18]. In contemporary India and Pakistan, we see their adoption of local languages (Gujarati and Sindhi) and economic integration while maintaining their ethnic identity and practicing strict endogamy [1, 3, 4, 12, 19–21].

Previous genetic analyses of the Parsis have focussed mainly on low-resolution uni-parental markers, which have suggested their affinity with both West Eurasian and Indian populations [22–24]. Autosomal analysis based on microsatellites or human leukocyte antigens (HLA) have revealed their intermediate position among the populations of South Asia and the Middle East/Europe [9, 24]. A study of mitochondrial DNA (mtDNA) variation reported 60% of South Asia lineages among the Pakistani Parsi population [23], whereas the male lineages based on Y chromosome admixture estimates were almost exclusively Iranian [22]. Based on these results, a male-mediated migration followed by assimilation of local South Asia females was concluded [23].

These early studies of the Parsi populations relied mainly on low-resolution markers, limiting the power of the analyses [9, 23–25], and the majority of Parsis (~98.8%), who live in India, have been underrepresented in these studies. Here we present genome-wide genotyping array data from 43 and high-resolution mtDNA and Y-chromosome genotyping data from 174 Parsi samples from India and Pakistan. In addition, we also genotyped mtDNA polymorphisms from 21 ancient Parsi samples excavated from Sanjan, in present-day Gujarat, India (Fig. 1). The human remains from Sanjan *dokhama* (tower of silence) District Valsad, Gujarat, were excavated in 2004. The accelerator mass spectrometry dating of human remains suggest that the *dokhama* belongs to the 14th to 15th century A.D. [17].

We investigated whether the current Parsi people living in India and Pakistan are genetically related amongst themselves and with the present-day Iranian population, and if their genetic composition has been affected by the neighboring Indian and Pakistani populations. We also examined runs of homozygosity (RoH) to study consanguinity. To address the extent to which the current Parsi populations assimilated local females during their long formation history, we compared their mtDNA haplogroup composition with ancient remains excavated in Sanjan, the initial settlement established by these migrants from Persia [17].

## Results and discussion

For autosomal analyses, we used Illumina HumanHap 650 K genotyping chips on 19 Indian Parsi samples collected from Mumbai, and Illumina 2.5 M genotyping chips for 24 Pakistani Parsi individuals from Karachi (Fig. 1 and Additional file 1). The combined Parsi data set was merged with a global data set from the published literature [26–29] and references therein] . Additional file 1: Table S2 lists the populations and number of single-nucleotide polymorphism (SNPs) used for various analyses after quality control (Additional file 1). The mean allele frequency differentiation between the two Parsi (Indian and Pakistani) groups was the lowest (*F*_ST_ Indian and Pakistani Parsi = 0.00033 ± 0.000025), followed by the differentiation of each from the Iranian population (0.011 ± 0.00021 and 0.012 ± 0.00025 for Pakistani and Indian Parsis, respectively), suggesting a common stock for both the Indian and Pakistani Parsis with the closest interpopulation affinity with populations from their putative homeland, Iran (Additional file 1: Figure S1 and Additional file 2: Table S3). Collectively, in *F*_ST_-based analysis, both of the Parsi groups showed a significantly closer connection with West Eurasians than any of the Indian groups (two-tailed *P* < 0.0001).

**Fig. 2:**
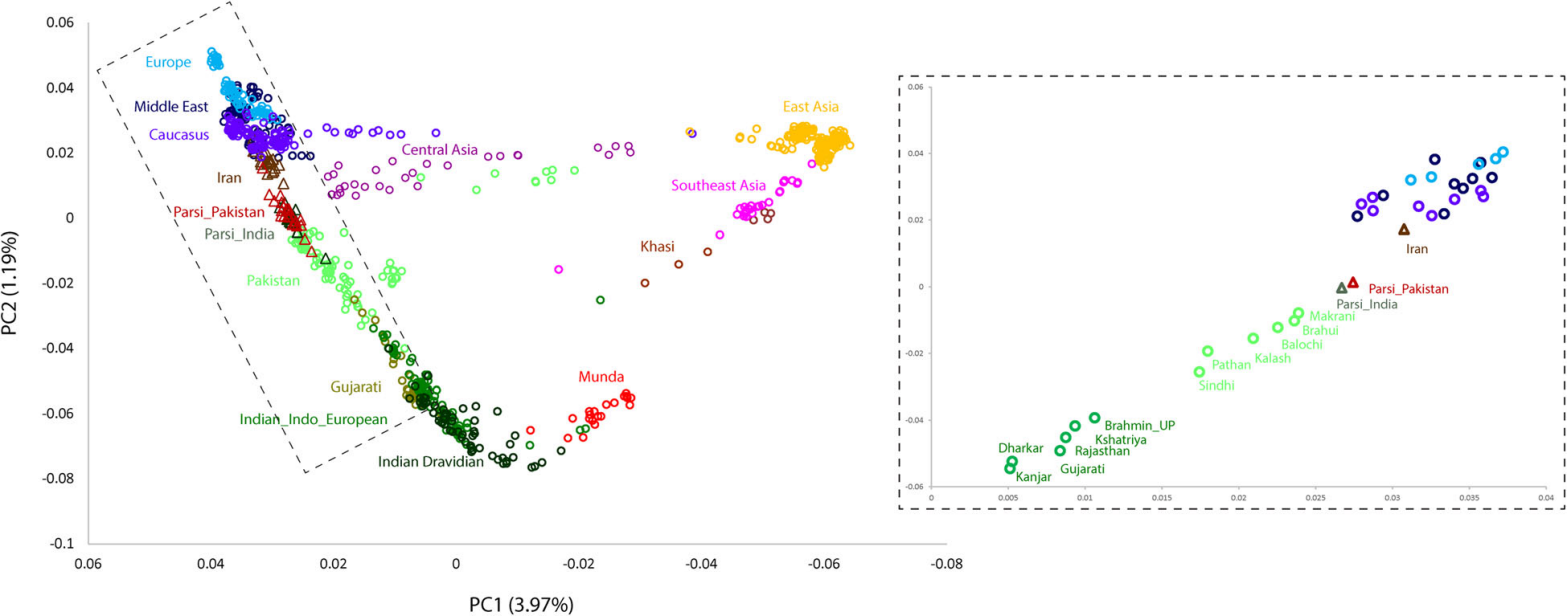
Principal component analysis of the combined autosomal genotypic data of individuals from Eurasia. The inset shows a plot of mean eigenvalues of Parsi and other close-by populations. *PC* principal component

**Fig. 3:**
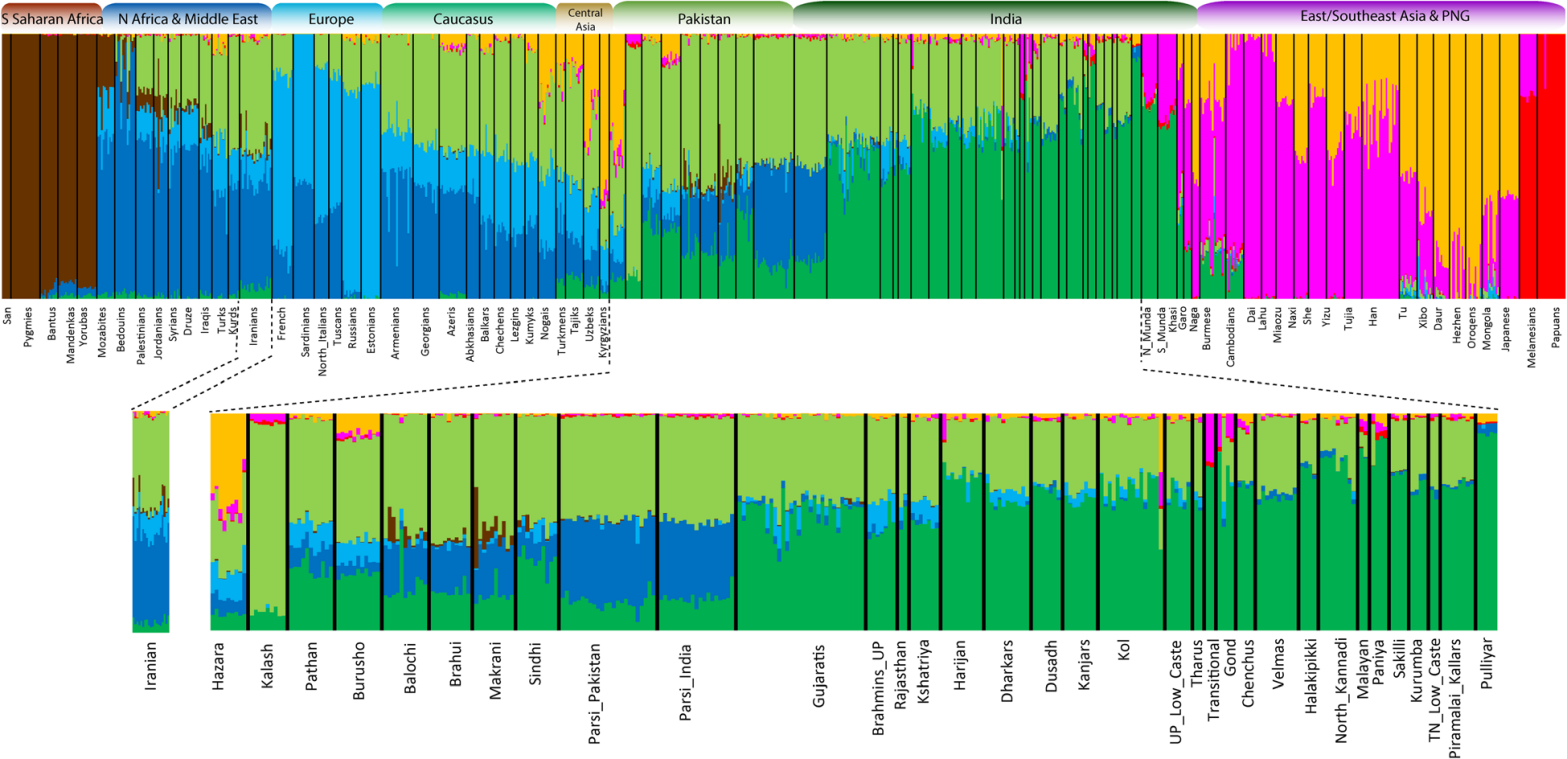
Results of ADMIXTURE analysis (*k* = 8) of world populations with a zoom-in on Iranian, Parsis, and other South Asian populations

We applied the default settings of the SMARTPCA program implemented in the EIGENSOFT package [30] and performed principal-components analysis (PCA) with other Eurasian populations using autosomal SNP data (Additional file 1: Table S2 and supplementary text). Our plot of the first and second principal components (PCs) clusters the Indian and Pakistani Parsis together, along the European–South Asian cline (Fig. 2). A plot of the population-wise mean of the eigenvalues showed their placement between the Pakistani and Iranian populations, indicating that the Parsis might have admixed from these two groups. Such an intermediate position of Parsis closer to Iranians than to their present geographic neighbors (Sindhi and Gujarati) suggests that the Parsis may have major ancestry from West Eurasians (Iranians) and minor ancestry with South Asians (Fig. 2 and Additional file 1: Figure S1). We next applied the model-based clustering method assembled in ADMIXTURE [31] to reveal the positioning of Parsis in the genetic structure canvas of Eurasia. The best model [28, 29] suggested eight major genetic components—sometimes also referred to as “ancestral populations”—and identified the presence of three of the components within the Parsi (Fig. 3 and Additional file 1: Table S4). The distributions of these components among Indian and Pakistani Parsis were unique and resembled each other, but were distinct from their neighboring South Asian populations (Additional file 1: Figure S2a). Supporting the *F*_ST_ and PCA results, the Middle-Eastern-specific (blue) ancestry component was significantly higher (two-tailed *P* < 0.0001) in the Parsis than in any other populations residing in South Asia that were examined. The present-day Iranian population exhibited a striking difference from the Parsis, mainly in carrying an additional European component (light blue) and substantially lower South Asian ancestry (dark green) (Fig. 3 and Additional file 1: Figure S2a and Table S4). Furthermore, the ancestral North Indian ancestry calculated from the *f*4 ancestry estimate showed a substantially higher level of this ancestry among Parsis than any other South Asia population (Additional file 1: Figure S2b).

It has been suggested previously that the Islamic conquest had a major genomic impact on several Middle Eastern populations, including Iranians [32]. Since Parsis diverged from Iranians just after this conquest, they may represent the genetic strata of Iran before the Islamic conquest. To test this scenario, we applied a formal test of admixture *f*3 statistics (Additional file 1: Table S5). For Iranians, a negative value with significant *Z* scores supports the hypothesis that they are descendants of a population formed by the admixture of Neolithic Iranians and populations from the Arabian Peninsula, while for Parsis this test was positive with significant *Z* scores. Therefore, it seems plausible that the additional light blue component we see in ADMIXTURE (Fig. 3) may have been introduced to Iran after the exile of the Parsis, likely via a recent gene flow from the Arabian Peninsula [32–34]. The quantitative estimation of South Indian and Iranian ancestries among Parsis and their South Asian neighbors showed a significant level of differentiation in ancestry composition with an inclination of Parsis towards Iranian ancestry (two-tailed *P* < 0.0001) (Table 1 and Additional file 1: Supplementary text).

**Table 1:** The South Indian and Iranian ancestry among Parsis and neighboring populations

Population (X)	South Indian (SE)	Iranian (SE)
Pathan	31 (2.4)	56.5 (1.8)
Sindhi	22.9 (2.7)	63.1 (1.9)
Parsi (Pakistan)	6.4 (2.4)	76.6 (1.5)
Parsi (India)	8.8 (2.5)	74.6 (1.7)
Gujarati	58.1 (2)	34.3 (1.6)

South Indian ancestry: (Yoruba, Papua; X, French/Yoruba, Papua; South India, French)

Iranian ancestry: (Yoruba, Papua; X, South India/Yoruba, Papua; Iranian, South India)

We computed *D* statistics [35] to determine the nature of the gene flow and admixture of Parsis with their parental (Iranian) and neighboring (Gujarati and Sindhi) populations (Table 2). Consistent with the previous analyses (Figs. 2 and 3), both of the Parsi populations shared a highly significant *D* value with each other. On the other hand, the South Asian populations (Gujarati and Sindhi) had significant levels of gene flow with each other, as well as with both of the Parsi populations when evaluated with respect to the present-day Iranian population (Table 2). Two independent studies have recently reported data from ancient Iranian samples [36, 37]. It was suggested that the early Neolithic Zagros sample showed closer affinity with the Iranian Zoroastrians [36]. Here we estimated the *D* values of Parsis for Neolithic Iranians vs modern Iranians to compare the allele sharing. Our results demonstrated a significant level of genetic affinity between Parsis and Neolithic Iranians (Table 2 and Additional file 1: supplementary text and Table S6). The outgroup *f*3 statistics of ancient Iranian samples supported the close affinity of the Parsis with Neolithic Iranians (Additional file 1: Supplementary text, Figure S3, and Table S6). Moreover, for modern populations, the outgroup *f*3 statistic test and identity-by-state plots supported the closer affinity of the Parsis with the West Eurasian populations than South Asians (Additional file 1: Figure S4 and S5). To compare the shared drift with Iranian and Indian (South Munda) populations (Additional file 1: Figure S6; see Additional file 1: supplementary text for justification of the use of South Munda to represent Indian ancestry), we plotted the derived allele sharing values of Parsis and other Eurasian populations calculated with respect to the Iranian and South Munda (Indian) populations (Additional file 1: supplementary text and Figure S6). This analysis aligned the Parsis closer to the Iranian axis between Pakistani and West Eurasian populations, supporting the historical interpretation of the most recent common ancestry of Parsis with the Iranians. A TreeMix [38] analysis supports these conclusions and shows the Parsis located between the South Asian and Iranians (Additional file 1: Figure S7).

**Table 2:** The test of geneflow (*D* statistics) between Parsis, modern Iranians, Neolithic Iranians, Sindhis, and Gujaratis

Gp1	Gp2	Gp3	*D* value	*Z* score
Parsi (India)	Parsi (Pakistan)	Sindhi	0.0309	35.229
Parsi (India)	Parsi (Pakistan)	Iranians	0.0318	42.175
Parsi (India)	Gujaratis	Sindhi	−0.0015	−1.89
Parsi (Pakistan)	Parsi (India)	Sindhi	0.0309	34.184
Parsi (Pakistan)	Parsi (India)	Iranians	0.0313	39.061
Parsi (Pakistan)	Gujaratis	Sindhi	−0.002	−2.482
Sindhi	Parsi (India)	Parsi (Pakistan)	0	−0.036
Sindhi	Parsi (India)	Gujaratis	−0.0038	−4.609
Sindhi	Parsi (Pakistan)	Gujaratis	−0.0038	−4.688
Sindhi	Iranians	Parsi (Pakistan)	−0.0124	−16.085
Sindhi	Iranians	Parsi (India)	−0.0124	−15.308
Sindhi	Iranians	Gujaratis	−0.016	−17.608
Gujaratis	Parsi (Pakistan)	Parsi (India)	−0.0005	−0.898
Gujaratis	Iranians	Parsi (Pakistan)	−0.0154	−21.245
Gujaratis	Iranians	Sindhi	−0.0211	−22.669
Gujaratis	Parsi (India)	Sindhi	−0.0054	−5.982
Gujaratis	Parsi (Pakistan)	Sindhi	−0.0058	−6.7
Iran (Neolithic)	Sindhi	Iranians	−0.0002	−0.141
Iran (Neolithic)	Gujaratis	Iranians	−0.008	−5.075
Iran (Neolithic)	Parsi (India)	Iranians	0.005	3.2
Iran (Neolithic)	Parsi (Pakistan)	Iranians	0.0058	4.083

*D* = (Gp1, Yoruba; Gp2, Gp3)

We computed a maximum likelihood tree and co-ancestry matrix based on the haplotype structure of the Parsi populations, applying the default settings of ChromoPainter and fineSTRUCTURE (version 1) [39]. The maximum likelihood tree split South Asian and West Eurasian populations into two distinct clusters (Additional file 1: Figure S8). All the Parsi individuals form a unique sub-cluster embedded within the major West Eurasian population trunk. The co-ancestry matrix plot clearly differentiated Parsis from their neighbors in sharing a large number of chunk counts with West Eurasian (mainly Iranian and Middle Eastern) populations (Additional file 1: Figure S9 and supplementary text). Additionally, South Asian populations have donated a significantly higher number of chunks to Parsis than they received from them (two tailed *P* < 0.0001). However, the number of these chunks were significantly lower than the chunk counts shared between any pair of South Asian populations (two-tailed *P* < 0.0001) (Additional file 1: Figure S9). The fineSTRUCTURE and *D* statistic results thus largely suggest unidirectional minor gene flow from South Asians to Parsis (Table 2 and Additional file 1: Figure S9).

We used ALDER, a method based on linkage disequilibrium [40], to estimate the time of admixture between Parsis and their neighboring South Asian populations. For this analysis, we used the present-day Iranian vs Gujarati or Sindhi populations as their surrogate ancestors. We estimated the admixture time of Parsi groups to be around ~40 generations (95% CI 26–50), which yields a time of 1160 years (assuming a generation time of 29 years), in good agreement with their historically recorded migration to South Asia (Table 3). We also tested evidence for a more complex admixture history using MALDER [41], which can be used to infer multiple admixture events. The MALDER analyses confirmed the ALDER results, demonstrating only one admixture event 54 ± 8 generations ago. The ancestral sources with the highest amplitude in MALDER were Sardinian and Dai (Additional file 1: Table S7).

**Table 3:** The formal text of admixture using the ALDER method

Reference 1	Reference 2	Admixed	Generation time	*P* value	*Z* score
Iranian	Gujarati	Parsi (India)	38.26 ± 12.16	0.0017	3.15
Iranian	Sindhi	Parsi (India)	32.96 ± 9.42	0.013	2.48
Iranian	Sindhi	Parsi (Pakistan)	41.32 ± 8.93	1.7 × 10^−5^	4.3
Iranian	Gujarati	Parsi (Pakistan)	30.74 ± 14.04	0.029	2.19

To investigate further the parental relatedness among Parsis [19], we analyzed the RoH and inbreeding coefficient in the population (Additional file 1: Figure S10). For RoH calculations, we applied three window sizes (1000, 2500, and 5000 kb), requiring a minimum of 100 SNPs per window and allowing one heterozygous and five missing calls per window. Long RoH segments characterize consanguinity and also provide a distinctive record of the demographic history for a particular population [42, 43]. As expected, both of the Parsi populations carried a larger number of long segments relative to their putative parental populations and present neighbors at the 1000-kb window length, likely due to the small population size and a high level of inbreeding. However, the Sindhi population from Pakistan also showed a higher level of inbreeding at the larger RoH window sizes, most likely due to an elevated level of cross-cousin marriages (Additional file 1: Figure S10).

To investigate how random genetic drift has shaped the functional genetic variation after admixture in the Parsis, we implemented the population branch statistic [44] using the Sindhi and Iranians as reference and outgroup. We analysed variants over the 99.9th percentile of the genomic distribution, focussing only on those that were annotated as missense, stop gain, stop loss, splice acceptor, or splice donor using the Ensembl Variant Effect Predictor tool [45]. This revealed a cluster of linked SNPs in the HLA region and a missense SNP in CD86 (rs1129055) with a high ancestral G allele frequency in the Parsi (0.87) (Additional file 1: Figure S11 and Table S8). The frequency of this G allele is lower in the Iranians and Sindhi (0.60) and other South Asians (0.52) and East Asians (0.40). This polymorphism has been associated with the pathogenesis of pneumonia-induced sepsis and the G allele has been associated with a decreased risk of active brucellosis in Iranians [46]. The G variant has also been associated as an eQTL for decreased expression of *IQBC1*, an IQ-motif-containing B1 gene that is highly expressed in Epstein–Barr virus-transformed B lymphocytes [47].

**Fig. 4:**
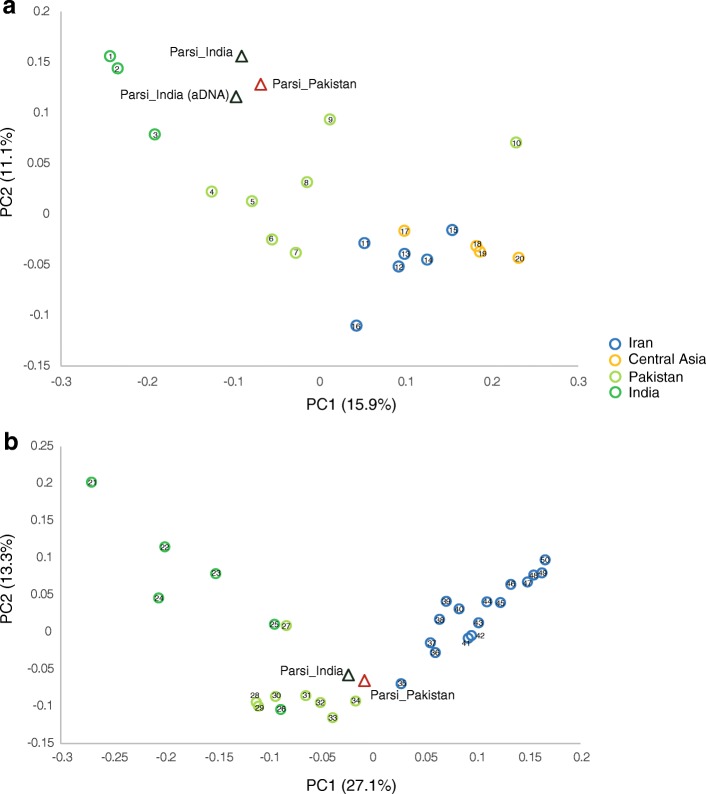
Principal component analysis using haplogroup frequencies for **a** mtDNA and **b** Y chromosome in Parsi, Iranian, Central Asian, Pakistani, and Indian populations. Details of the populations are given in the supplementary text. *aDNA* ancient DNA, *PC* principal component

To obtain a detailed understanding of the sex-specific South Asian and Iranian ancestries, we examined maternally inherited mtDNA and paternally inherited Y chromosome biallelic polymorphisms in a larger sample in both the Indian and Pakistani Parsi populations (Additional file 1: Figure S12, Tables S9 and S10). For the mtDNA analysis, we were also able to assay 21 ancient samples from the Sanjan [21] region, for 108 diagnostic polymorphisms (Additional file 1: Table S11 and supplementary text). Interestingly, we observed 48% South-Asian-specific lineages (haplogroups M2, M3, M5, and R5) among the ancient Parsi samples, which could potentially be explained in two ways. First, these haplogroups might have been carried by the migration of Zoroastrian refugees from Fars (Iran), a possibility that is supported by the presence of these clades in present-day Persian samples (9.9%) [34]. Second, they might have resulted from the assimilation of local females during the initial settlement. The comparison of ancient and modern samples thus identified maternal lineages that can be considered as founding (surviving or lost), as well as those that were subsequently assimilated (Additional file 1: Figure S12 and Tables S9–11). The Y chromosome profiles of Indian and Pakistani Parsi populations revealed a higher frequency of Middle-Eastern-specific lineages than South Asian ones in the Parsis (Additional file 1: Figure S12 and Table S10). The PC analysis of both mtDNA and Y chromosome data placed all the Parsi groups close to each other and showed their contrasting clustering based on maternal or paternal ancestries (Fig. 4). For mtDNA, the Parsi cluster was closer to the Indian and Pakistani cluster (Fig. 4a), whereas for the Y chromosome it aligns between the Iranian and Pakistani populations (Fig. 4b). The Ychromosomal PCA is similar to the autosomal PCA (Fig. 2 and Additional file: Figure S1). The contrasting patterns of maternal and paternal ancestry support a largely female-biased admixture from the South Asian populations to the Parsis.

## Conclusions

In conclusion, our investigation has not only contributed substantial new data, but has also provided a more comprehensive insight into the population structure of Parsis and their genetic links to Iranians and South Asians. We show that the Parsis are genetically closer to Iranian and Caucasian populations than those in South Asia and provide evidence of sex-specific admixture with the prevailing female gene flow from South Asians to the Parsis. Our results are consistent with the historically recorded migration of the Parsi populations to South Asia in the 7th century and in agreement with their assimilation into the Indian sub-continent’s population and cultural milieu “like sugar in milk”.

## Methods

A detailed description of the material and methods can be found in the supplementary text (Additional file 1). The modern Parsi samples were pooled from three independent collections: two from Mumbai, India, and one from Karachi, Pakistan (Fig. 1). Illumina 650 K and 2.5 M chips were used to genotype 19 Indian and 24 Pakistani Parsi individuals, respectively, following the manufacturer’s specifications. We merged our newly generated data of 43 samples with the relevant reference data sets of 829 samples published elsewhere (Additional file 1: supplementary text and Table S2). For mtDNA control and coding region polymorphisms, we genotyped 117 Indian and 50 Pakistani Parsi samples (Additional file 1: Table S9). We followed phylotree (build 17) to classify them into haplogroups. For Y chromosome genotyping, 90 Pakistani samples were genotyped either by sequencing or by (PCR-RFLP) Polymerase Chain Reaction- Restriction Fragment Length Polymorphism for the relevant Y chromosome markers, whereas 84 Indian samples were assayed for 80 Y chromosomal SNPs using Sequenom mass array technology (Additional file 1: Table S10).

Ancient DNA samples were excavated from Sanjan, Gujarat, in 2001 (Additional file 1: supplementary text). Archaeological analysis and accelerator mass spectrometry dating were consistent with these remains belonging most likely to migrant Parsis from the 8–13th centuries (Additional file 1: supplementary text). The teeth obtained from 21 of these specimens were analyzed at the ancient DNA laboratory of the Centre for Cellular and Molecular Biology of the Council of Scientific and Industrial Research (CSIR), Hyderabad, India. We followed our standard published protocol to isolate DNA from teeth [48] (Additional file 1: supplementary text).

For autosomal analyses, after data curation and merging (Additional file 1: supplementary text), we first used the method of Cockerham and Weir [49] to estimate the mean pairwise *F*_ST_. Further, we performed PCA on pruned data using SMARTPCA v.7521 [30] (with default settings). We also used the *F*_*ST*_:Yes method of SMARTPCA to calculate the *F*_*ST*_ with standard errors. We ran unsupervised ADMIXTURE v.1.23 for 25 times for each *K* = 2 to *K* = 12, and used the method described previously to choose the best *K* value [28–29]. The *F* statistics were calculated by the ADMIXTOOLS package v.3 [35] and the haplotype-based analysis was performed by Chromopainter and fineSTRUCTURE v.1 [39]. The maximum likelihood tree of world populations was constructed using TreeMix v.1.12 [38] and the RoH were calculated using PLINK 1.9 [50]. ALDER v 1.03 [40] and MALDER v.1.0 [41] were used to calculate the time and number of admixture events. The population branch statistic method [44] was used to identify genomic regions under selection in the Parsi population.

## Additional files

Additional file 1: Supplementary text explaining the archeological details of ancient samples; isolation of ancient DNA, genotyping, statistical analyses and peopling of South Asia and Parsi chapters. 12 figures and 10 tables are also incorporated in this file. (PDF 13264 kb)

Additional file 2: Table S3. The population-wise *F*_ST_ values based on an autosomal data set for all the populations included in this study. (XLSX 120 kb)
