# Supplementary material for: “Like sugar in milk”: reconstructing the genetic history of the Parsi population
Source: Genome Biol. 2017 Jun 14;18:110. doi: 10.1186/s13059-017-1244-9 (PMC5470188; doi:10.1186/s13059-017-1244-9)
Supplement: Supplementary file 1 — Supplementary text explaining the archeological details of ancient samples; isolation of ancient DNA, genotyping, statistical analyses and peopling of South Asia and Parsi chapters. 12 figures and 10 tables are also incorporated in this file. (PDF 13264 kb) [file 13059_2017_1244_MOESM1_ESM.pdf]

Contents

Sample details 3
Ancient Samples 3
Modern samples 4
Ancient DNA extraction from tooth 5
Statistical analysis 6
Peopling of South Asia and Parsi 9
Additional File 1: Figure and Table Legends: 11
Fig. S1: Mean pairwise F_{ST} comparison of Parsis with other worldwide population groups based on autosomal SNP data. 11
Fig. S2: a) The plot of mean values of eight ancestry components (k=8) for Iranian, Pakistani, Parsis and Gujarati populations. Data is extracted from Fig. 3. b) The ANI (Ancestral North Indian) ancestry for Iranian, Pakistani, Parsis and Gujarati populations, obtained from f4 ancestry ratio estimate. 11
Fig. S3: Outgroup f3 statistics for modern samples used in the present study with respect to the Iranian and Levant ancient samples. 11
Fig. S4: The plot of shared drift obtained by the f3 =(Yoruba; Iranian/Parsi_Pakistan/Parsi_India/Sindhi/Gujarati, X). Where X is another Eurasian populations. 11
Fig. S5: The plot of sharing of mean IBS (Identity-by-state) values of Iranian, Sindhi, Gujarati and Parsis with respect to other worldwide populations. 11
Fig. S6: The f3 values of Eurasian populations plotted on Iranian ancestry (Y axis) against the Indian Ancestry- South Munda (X- axis). 11
Fig. S7: Relationship among Parsi and other Eurasian populations inferred using the Maximum Likelihood (ML) method implemented in TreeMix. 11
Fig. S8: Placement of Indian and Pakistani Parsi individuals over the 35 clades obtained from the fineSTRUCTURE analysis. The frequency of individuals of a population in a particular clade is displayed. Different color codes of populations correspond to the color codes used in autosomal PCA analysis (Figure 2). 11
Fig. S9: Mean sharing of DNA segments (chunkcounts) of Parsis and their neighbours with other Eurasian populations. The chunk sharing of Parsis with respect to their close neighbours (Gujarati and Sindhi) is shown on the right-hand side. 11
Fig. S10: The plot of mean count (upper panel) and inbreeding coefficient F (lower panel) vs length of runs of homozygosity (RoH) segments among Iranians, Parsis, Gujarati and Sindhi populations. We used three different windows of 1,000 kb, 2,500 kb and 5,000 kb. In the lower panel the number of total segments is shown inside each circle. 11
Fig. S11: The F_{ST} based branch length for Parsi, Iranian and Gujaratis; b) Graphical summary (Manhattan plot) of the outlier autosomal SNPs in the Parsis using the population branch statistics (PBS) (details given in Supplementary Table 5); c) F_{ST} based branch length for genomic region under selection. 11
Fig. S12: The summary and distribution of mtDNA (upper panel) and Y chromosome (lower panel) topology among modern and ancient Parsi samples 12
Table Legends: 12
Table S1. The arrival and expansion of Parsis in Indian subcontinent. The details have been obtained from Parzor foundation, New Delhi, India 12
Table S2. Details of the modern populations, number of samples and SNPs used in various analyses based on autosomal data 12
Table S4. The raw values of eight ancestral components used to plot Fig. 3. 12

Table S5. Formal test of admixture in the form of f3 (Iran_Neolithic,X;Y); where X represent Arabian and Middle Eastern populations and Y represent Iranian and Parsi populations. ... 12
Table S6. Computed D Statistic results showing geneflow between Neolithic Iranian and other modern populations. ... 12
Table S7. The MALDER test showing the single admixture event in the Parsis. ... 12
Table S8. Functional annotation of variants over the 99.9th percentile of the distribution using the population branch statistics. ... 12
Table S9. The control and coding region mtDNA mutations for Indian and Pakistani Parsi populations. ... 12
Table S10. The Y chromosome haplogroups frequency in the Indian and Pakistani Parsi groups. ... 12
Table S11. List of diagnostic sites and the results of mtDNA genotyping in 21 ancient Parsi tooth samples. ... 12
References ... 13

Sample details

Ancient Samples

The ancient site of Sanjan (N 20º11'59.6, E 72º48'00.2) was first discovered in 2001. The ancient mound is situated about two kilometers to the north of the modern town of Sanjan on the northern bank of the Varoli River/Creek in the Umargam Taluka of Valsad District, Gujarat, India (Fig. 1; Additional File 1: Table S1). Medium scale excavation were carried out for three seasons, jointly by the World Zarathusti Cultural Foundation, Mumbai, and The Indian Archaeological Society, New Delhi, at three different locations of the site, Sanjan *bandar* (SJN-B), Sanjan kolikhadi (SJN-K) and Sanjan *dokhama* (SJN-D). Excavations conducted over three field seasons have revealed a large number of artefacts, ceramics and structural remains belonging to the period 8th-13th centuries A.D. [1,2].

Sanjan *dokhama* or the Tower of Silence was the only structure found in Season III. At the confluence of Kolikhadi and the Tukkar Nala, a steep mound with a circular depression at its top was found. This mound, according to local tradition, was a "bhastu" – a local word for a *dokhama*. The *dokhama* is a mortuary structure consisting of a large circular wall enclosing a raised platform, at the center of which there is a dry well. This is uniquely Zoroastrian in nature. The dead body is exposed to the natural elements (devoid of garments and ornaments) on this platform and after a time, any osseous remains are swept into the central well. It is in this manner that the Parsis adhere to the Zoroastrian strictures against the pollution of the elements by the dead and, thus, this structure strongly suggests a settlement of the Parsi community. It was decided to take up excavations at this mound, because the presence of a *dokhama* would be the clinching evidence of a Parsi occupancy at Sanjan during the 8th to the 13th century, and make this site the oldest Parsi settlement ever to be excavated.

For precise dating human bones recovered from the *dokhama* were sent to Radiocarbon Accelerator Unit, Research Laboratory for Archaeology and the History of Arts, Oxford University, Oxford, United Kingdom for AMS dating. The result gives very consistent dates confirming that the *dokhama* was in use in the 14th and 15th century A.D. [3]. Careful excavation of the dokhama revealed the tentative remains of at least 180 individuals in about 40% of the *bhandar*. Exact estimates were not possible on site as the bones were disarticulated and lying scattered, showing no specific arrangements or orientation. Tentative estimates were based on the fact that approximately 134 skulls or parts thereof were recovered. The remains were those of a wide spectrum of age groups – newborns,

children, adults and geriatric. Besides human bones the *dokhama* also yielded a large number (or fragments) of glass bangles, rings of copper, silver and mixed metal, and beads of glass. Parsi women wore, and continue to wear, glass bangles as a symbol of their married status, and in some instances rings as a toe ornament. These traditions were borrowed from contact with autochthonous Indian groups, as the same were not extant in Iran. A tradition grew whereby women were interred upon death into the *dokhama* with their ornaments. This tradition probably discontinued only about 200 years ago. Excavations in the *bhandar* also revealed a small gold bead and a copper earring with gold caps at either end.

Modern samples

The modern samples in the present study were collected from both India and Pakistan (Fig. 1). The Indian Parsi samples were from Mumbai, whereas Pakistani samples were from Karachi. Informed consent was obtained from all participating individuals and relevant institutional review boards in India and Pakistan. DNA was extracted from either whole blood or lymphoblastoid cell lines (Parsi from Pakistan) using a standard organic extraction protocol [4]. The autosomal genotyping for Indian and Pakistani samples were done on different platforms. The Indian Parsis were genotyped on Illumina HumanHap 650K array, whereas the Pakistani Parsis were assayed on Illumina HumanOmni 2.5M chip. We merged both of the datasets together with other Illumina arrays published elsewhere [5-11]. After quality control and filtering chromosomes 1-22, we obtained 289,273 high quality SNPs and used these in the subsequent analysis (Additional File 1: Table S2). For mitochondrial DNA (mtDNA) the HVS regions (I and II) were sequenced via Sanger sequencing method. We confirmed the haplogroup affiliation by genotyping the mtDNA coding region variations. All the individuals were haplogrouped based on their control and coding region mutations by following the latest nomenclature of Phylotree [12] (Additional File 1: Table S9). For the Y chromosome, we used a combination of assays covering 80 SNPs that were genotyped by Sequenom MassARRAYiPLEX platform for the Indian Parsi samples. Based on the SNP genotypes we designated them into haplogroups followed by writing the name of the terminal marker (Additional File 1: Table S10). The following populations were used to make PCA plot of mtDNA and Y chromosome (Fig. 4): mtDNA- (1) Charan, (2) Bharwad, (3) Gujarati, (4) Sindhi, (5) Pathan, (6) Baluch, (7) Brahui, (8) Burusho, (9) Hazara, (10) Kalash, (11) Kurd, (12) Gilaki, (13) Persian, (14) Turkish, (15) Lur, (16) Mazamdarian, (17) Uzbek, (18) Shugnan, (19) Turkmen, (20)

Kurdish; Y chromosome- (21) Gujarati Patel, (22) Gujarati mixed 1, (23) Gujarati 1, (24) Bharwad, (25) Gujarati 2, (26) Charan, (27) Kalash, (28) Gujjar, (29) Punjabi, (30) Burusho, (31) Sindhi, (32) Pathan, (33) Balochi, (34) Brahui, (35) Baluch, (36) Queshmi, (37) Afro-Iranian, (38) Persian, (39) Mazandarani, (40) Turkmen, (41) Kurd, (42) Yazd, (43) Zoroastrian, (44) Fars, (45) Azeri, (46) Gilak, (47) Arab, (48) Lur, (49) Armenian, (50) Assyrian.

Ancient DNA extraction from tooth

All equipment were cleaned and exposed under UV before starting the experiment. Required reagents (50% bleach, 70% alcohol, di butyl tinlaurate, and silicone rubber) were prepared before starting the experiment. Silicone rubber which is in semi solid form was mixed with dibutyl tin laurate for solidification and immediately placed into a mould. Each pre cleaned tooth sample was placed in the mould and left for one day. Once the silicone rubber was completely solidified, and the tooth sample was completely settled, a dermal cut off wheel was used to cut the tooth root. Through the tooth root, a small hole was made using dremel bids and the tooth powdered by engraving the pulp and dentine inner surface. About 30 mg of powder was prepared and transferred to an autoclaved Eppendorf (1.5 ml) tube, dissolved in 500 µl of 0.5mM EDTA and incubated at 37^0C using a rotatory shaker. After overnight incubation the lysate was centrifuged at 15,000 rpm for 10 minutes. The supernatant was taken out and mixed with 5 volumes (2.5 ml) of PB buffer (Qiagen), passed through silica column (Qiagen) and finally eluted with 50 µl of low TE buffer.

Multiplex PCR and genotyping using Sequenom

Degraded ancient DNA samples were genotyped using Sequenom iPLEX assay and the MassARRAY system (SEQUENOM, San Diego, CA). Sequenom based method requires low amount of DNA (in picograms) and has ability to work with highly degraded small-size amplicons (~ 100 bp). Therefore, this technique is the most appropriate for PCR based genotyping of ancient DNA.

Various concentrations of ancient DNA (100pg – 1ng) were amplified in a 5.0 µl multiplexed PCR reaction, following manufacturer's instructions. Multiplex PCR was performed in GeneAmp 9700 thermal cycler (Applied Biosystems) using 10X PCR buffer, primer mix, dNTPs and HotStarTaq (Qiagen, Hilden, Germany). The PCR conditions used for amplification were; initial denaturation at 94 ˚C for 15 min for hot start, followed by 45 cycles: denaturation at 94˚C for 20 sec, annealing at 56˚C for 30 sec, extension at 72˚C

for 1 min, and a final extension at 72˚C for 3 min. After PCR, the products were treated with shrimp alkaline phosphatase (SAP, Amersham, Freiburg, Germany) to dephosphorylate the left over dNTPs. The reaction conditions were for 37˚C for 20 minutes to remove any remaining dNTPs, followed by 85˚C for 30 minutes to inactivate the SAP and a final incubation at 4˚C until plate removal from thermal cycler. After SAP treatment, multiplexed iPLEX reaction was performed. The iPLEX reaction components were; 10X iPLEX buffer, iPLEX termination mix, primer mix and iPLEX enzyme. The iPLEX reaction conditions were: 94°C for 30 sec. followed by 40 cycles of 94°C for 5 sec., [5 cycles (52°C for 5 sec., 80°C for 5 sec)] and final extension at 72°C for 3 minutes followed by incubation at 4˚C till the plate was removed from the thermal cycler. The iPLEX reaction mixtures were then cleaned by adding 6 mg cationic resin SpectroCLEAN (Sequenom) using dimple plate and 16 µl of water. The plate was then sealed and placed in a rotating shaker for 20 min to desalt the iPlex solution. Completed iPLEX reaction products were spotted in nanoliter volumes onto a matrix-arrayed silicon chip with 384 elements (Sequenom SpectroCHIP) using the MassARRAY Nanodispenser. SpectroCHIPs were analyzed using the Autoflex MALDI-TOF Mass Spectrometer (Brukar Daltonics), and the spectra were processed using the SpectroTYPER V4 software (SEQUENOM). For ancient mtDNA analysis, 4 sets of pool were designed, which contains 115 variable positions to genotype the haplogroup defining motifs of the entire mitochondrial DNA (Additional File 1: Table S11).

Statistical analysis

For autosomal data management, we used PLINK 1.9 [13]. For modern samples, we have listed the population details and total number of SNPs in Additional File 1 and Table S2. Additionally, for some of the tests (e.g. formal test of admixture *f*3, outgroup *f*3 and *D* statistics), we have independently merged several ancient samples (Additional File 1: Fig. S3 and Tables S5 and S6), published elsewhere [14].

For modern samples, in order to remove one SNP in a pair with high LD (linkage disequilibrium), we thinned the dataset with parameters; $r^2 > 0.4$ in a window of 200 SNPs (sliding the window by 25 SNPs). After the LD pruning, the final dataset carried 177,683 SNPs (Additional File: Table S2). To estimate the relatedness of the populations, we first calculated mean pairwise differences between different populations (Additional File 2: Table S3) as well as different population groups (Additional File 1: Fig. S1a) independently, using the F_{ST} distance measure as described by Cockerham and Weir [15].

We have also used SMARTPCA programme [16] with 'FstOnly' option to validate the results of F_{ST}. In addition, this programme also enabled us to calculate the standard errors for the F_{ST} values. In populationwise F_{ST} analysis, Pathan and Brahmins_UP were closest to Parsis among Pakistani and Indian populations respectively (Additional File 2: Table S3). For the groupwise calculations, we have grouped Azeri, Armenian and Georgian as North Caucasus; Abakhasian, Balkars, Chechens, Lezgins, Kumyks and Nogais as South Caucasus; Kalash, Pathan and Burusho as North Pakistan and; Balochi, Brahui, Makrani and Sindhi as South Pakistan.

We performed a principal component analysis using the *smartpca* programme (with default settings) of the EIGENSOFT package [16] on LD pruned data of Eurasian population individuals as well as estimated the mean population wise eigenvalues from the same run (Fig. 2). Moreover, to have a closer look at the Parsi and their genetically related populations, we ran the same analysis on European, Middle Eastern, Caucasian, Pakistani, Indian Indo-European and Parsis populations (Additional File 1: Fig. S1b). We ran ADMIXTURE with a random seed number generator on the LD-pruned dataset twenty-five times at K = 2 to K = 12. We plotted K=8 as a best K based on the methods applied in our previous analyses [9,17] (Fig. 3 and Additional File 1: Table S4). To compare different ancestry component existing among Parsi, Iranian, Pakistani and Gujarati populations, we extracted and plotted the population wise mean values of all the ancestry components present among these populations (Additional File 1: Fig. S2a).

The ANI (Ancestral North Indian) ancestry was calculated via *f4* ancestry estimation test: *f*4 = (Yoruba,French;X,Munda_South/Yoruba,French;Georgian,Munda_South); where X= Iranian/Pakistani/Parsi/Gujarati [18] (Additional File 1: Fig. S2b). The *f*4 ancestry test was also used to estimate the South Indian (SIND) as well as Iranian specific ancestries (Table 1). For SIND ancestry, *f*4 ancestry ratio = (Yoruba,Papuan;X,French/Yoruba,Papuan;South_India,French; where X= Iranian/Pathan/Sindhi/Parsi/Gujarati. For SIND, we grouped the South Indian populations (e.g. Paniya, Malayan and Pulliyar) as a reference population withthe least amount of ANI ancestry. To estimate Iranian ancestry in the same set of the populations: *f*4 =(Yoruba,Papuan;X,French/Yoruba,Papuan;Iranian,French); where X= Iranian/Pathan/Sindhi/Parsi/Gujarati (Table 1).

We also compared the variation of various ancestry components as well as ANI ancestry ratio of Parsis with the Pakistani and Gujarati populations. In the admixture plot, the

Parsis showed a significant difference with the Pakistani populations in sharing the Middle Eastern component (blue) (two tailed p value < 0.0001). Moreover, the ANI (Ancestral North Indian) ancestry of Balochi, Brahui and Makrani were significantly lower from Parsis (two tailed p value < 0.0001) (Additional File 1: Fig. S2).

To test the geneflow from Islamic invasion to the modern Iranian populations, we have applied formal test of admixture *f*3 [18] (Additional File 1: Table S5). We took Neolithic Iranian as source 1 and other Middle Eastern populations (Mozabites, Bedouin, Palestinians, Jordanians, Syrians, Druze, Iraqis, Turks and Kurds) as source 2. We tested if modern Iranians are admixed group, of populations related to Neolithic Iranians and Levantines in comparison with the Parsis. The results were negative with significant Z scores, suggesting that modern Iranians are descended from a population formed by the mixing of Neolithic Iranians and Levantine populations, whereas the Parsis showed a positive values with significant Z scores. These results suggested that the modern Iranians and Parsis descended from distinct ancestral populations (Additional File 1: Table S5).

To evaluate the gene flow between modern as well as ancient populations, we applied the *D* statistics [18] with different combinations (Table 2; Additional File 1: Tables S5 and S6). We mainly focused on the gene flow between Indian and Pakistani Parsis and their present-day geographic neighbours (Gujarati, Sindhi) and putative ancestral populations (modern and ancient Iranians). To test whether Parsis share more alleles either with modern or Neolithic Iranians, we merged our data with the ancient Iranian samples published in Lazaridis *et al.* [14]. Due to the low quality of SNPs as well as limited number of common SNPs, several ancient samples failed to give high quality of large number of SNPs, therefore we have used samples id I1290 in most of our analyses (Table 2; Additional File 1: Tables S5 and S6). The merge of I1290 with our data has yielded 208374 SNPs. The total number of common SNPs went significantly down (27472) after adding additional Neolithic sample (I1945). However, it has also supported the higher geneflow of Neolithic Iranians with the Parsis. To validate the result, we have also compared HotuIIIB, Late Neolithic, Chalcolithic and Levant Neolithic samples with different number of SNPs for *D* statistics as well as for outgroup *f*3 statistics (Additional File 1: Fig. S3 and Table S6).

To investigate the derived allele sharing of Indian and Pakistani Parsis with other Eurasian populations, we computed outgroup *f3* statistics [18]. An allele sharing plot was constructed specifically to compare the alleles shared with other Eurasian populations

with respect to the Parsis from India and Pakistan, Iranians, Sindhis and Gujaratis (Additional File 1: Fig. S4). The IBS distance was obtained by PLINK 1.9 [13] and plotted against Parsis and their contemporary populations (Additional File 1: Fig. S5). Among Indian populations the South Munda populations carried the highest ASI (Ancestral South Indian) ancestry ([17,19], therefore we have selected South_Munda group to represent Indian ancestry. Outgroup *f*3 values of Eurasia populations with respect to Iranian *vs* Indian South_Munda groups were plotted to compare the shared drift of Parsis with other Eurasian populations (Additional File 1: Fig. S6). We constructed the Maximum Likelihood tree of world populations using TreeMix [20] (Additional File 1: Fig. S7). For haplotype based analysis we used fineSTRUCTURE [21]. A subset of the genotyped samples (Additional File 1: Table S2) were phased with Beagle 3.3.2 [22]. A coancestry matrix was constructed using ChromoPainter [21] and fineSTRUCTURE was used to perform an MCMC iteration using 10^7 burning runtime and 10,000 MCMC samples. A tree built using fineSTRUCTURE with the default settings showed 82 clusters with several single samples making unique clusters. After one step merge we obtained 53 clusters and 35 clusters after two steps merge (Additional File 1: Fig. S8). The co-ancestry matrix was plotted for the Indian and Pakistani Parsis, as a recipient of number of chunks from one another as well as from other Eurasian groups (Additional File 1: Fig. S9).

To estimate the admixture time we used the ALDER software [23], comparing Parsis and local Indian as well as Pakistani populations. For the Indian and Pakistani neighbouring reference populations, we used their present day neighbours, the Gujarati and Sindhi populations, respectively, whereas we took Iranians from Iran as surrogate populations (Table 3). To find out the number of admixture event(s), we also used MALDER [24] (Additional File 1: Table S7). The Runs of Homozygosity (RoH) were calculated using PLINK 1.9[13] (Additional File 1: Fig. S10). For RoH estimation, we applied three different window sizes (1,000 kb, 2,500 kb and 5,000 kb), a minimum of 100 SNPs per window allowing one heterozygous and five missing calls per window. We applied the population branch statistic method (PBS) [25] using as reference and outgroup populations the Sindhi and Iranians (Additional File 1: Fig. S11 and Table S8).

Peopling of South Asia and Parsi

Due to their geographic location and diverse variation in phenotype, South Asian populations are of considerable interest to biological anthropologists and geneticists. The haploid DNA studies are in coherence with a deep maternal ancestry of the

subcontinent which has been reshaped by minor influence of East and West Eurasian lineages [26-28], and Y chromosomal studies have provided a male perspective [27-31]. More recently the autosomal data has helped improve our understanding of the demographic history of South Asia in more details [9,32-34]. All the autosomal studies have consistently identified two major components among South Asian populations; an ASI (Ancestral South Indian) component that is mainly present in the Indian sub-continent, and an ANI (Ancestral North Indian) component that is shared with populations from Central Asia, the Caucasus, Middle East and Europe. Recently, ancient DNA (aDNA) studies, (though not directly on South Asian ancient remains), have suggested that the ANI component in South Asia is closely related with Caucasus Hunter Gatherer (CHG)[35] or Neolithic Iranian [36], or that it might represent an amalgamation of Neolithic Iranian and Steppe ancestries [37]. However, surprisingly the proposed Steppe ancestry was much more widely distributed geographically than it was suggested by the linguists and Indologists [38-40], therefore, aDNA data from India is of utmost importance to resolve these complexities.

The Parsis also carry both of the ancestries widely reported among majority of Indian populations. However, the amount of these ancestries are significantly different (two tailed p value < 0.0001), from their present day neighbours in South Asia (Table 1; Additional File 1: Fig. S2 and Tables S2 and S4). Moreover, we have observed that the genetic contribution from the Parsis to South Asians is negligible, likely due to small number of founders of Parsis, coupled with high level of endogamy practices in South Asia (Fig. S9). Therefore, they do not appear to have had any major impact on the large South Asian genepool. However, in context of Middle Eastern (more specifically Iran and adjoining regions), the genetic architecture of Parsi may signify the Iranian ancestry before the Islamic conquest, which had a major impact on the peopling of this region [41] (Additional File 1: Table S5).

Additional File 1: Figure and Table Legends:

Fig. S1: Mean pairwise F_{ST} comparison of Parsis with other worldwide population groups based on autosomal SNP data.

Fig. S2: a) The plot of mean values of eight ancestry components (k=8) for Iranian, Pakistani, Parsis and Gujarati populations. Data is extracted from Fig. 3. **b)** The ANI (Ancestral North Indian) ancestry for Iranian, Pakistani, Parsis and Gujarati populations, obtained from *f*4 ancestry ratio estimate.

Fig. S3: Outgroup *f*3 statistics for modern samples used in the present study with respect to the Iranian and Levant ancient samples.

Fig. S4: The plot of shared drift obtained by the *f3* =(Yoruba; Iranian/Parsi_Pakistan/Parsi_India/Sindhi/Gujarati, X). Where X is another Eurasian populations.

Fig. S5: The plot of sharing of mean IBS (Identity-by-state) values of Iranian, Sindhi, Gujarati and Parsis with respect to other worldwide populations.

Fig. S6: The *f*3 values of Eurasian populations plotted on Iranian ancestry (Y axis) against the Indian Ancestry- South Munda (X- axis).

Fig. S7: Relationship among Parsi and other Eurasian populations inferred using the Maximum Likelihood (ML) method implemented in TreeMix.

Fig. S8: Placement of Indian and Pakistani Parsi individuals over the 35 clades obtained from the fineSTRUCTURE analysis. The frequency of individuals of a population in a particular clade is displayed. Different color codes of populations correspond to the color codes used in autosomal PCA analysis (Figure 2).

Fig. S9: Mean sharing of DNA segments (chunkcounts) of Parsis and their neighbours with other Eurasian populations. The chunk sharing of Parsis with respect to their close neighbours (Gujarati and Sindhi) is shown on the right-hand side.

Fig. S10: The plot of mean count (upper panel) and inbreeding coefficient F (lower panel) *vs* length of runs of homozygosity (RoH) segments among Iranians, Parsis, Gujarati and Sindhi populations. We used three different windows of 1,000 kb, 2,500 kb and 5,000 kb. In the lower panel the number of total segments is shown inside each circle.

Fig. S11: The F_{ST} based branch length for Parsi, Iranian and Gujaratis; b) Graphical summary (Manhattan plot) of the outlier autosomal SNPs in the Parsis using the population branch statistics (PBS) (details given in Supplementary Table 5); c) F_{ST} based branch length for genomic region under selection.

Fig. S12: The summary and distribution of mtDNA (upper panel) and Y chromosome (lower panel) topology among modern and ancient Parsi samples.

Table Legends:

Table S1. The arrival and expansion of Parsis in Indian subcontinent. The details have been obtained from Parzor foundation, New Delhi, India.

Table S2. Details of the modern populations, number of samples and SNPs used in various analyses based on autosomal data

Table S4. The raw values of eight ancestral components used to plot Fig. 3.

Table S5. Formal test of admixture in the form of *f*3 (Iran_Neolithic,X;Y); where X represent Arabian and Middle Eastern populations and Y represent Iranian and Parsi populations.

Table S6. Computed *D* Statistic results showing geneflow between Neolithic Iranian and other modern populations.

Table S7. The MALDER test showing the single admixture event in the Parsis.

Table S8. Functional annotation of variants over the 99.9th percentile of the distribution using the population branch statistics.

Table S9. The control and coding region mtDNA mutations for Indian and Pakistani Parsi populations.

Table S10. The Y chromosome haplogroups frequency in the Indian and Pakistani Parsi groups.

Table S11. List of diagnostic sites and the results of mtDNA genotyping in 21 ancient Parsi tooth samples.

References

1. Gupta S, Dalal K, Dandekar A, Nanji R, Mitra R, Pandey R, et al. A Preliminary Report on the Excavations at Sanjan. Puratattva 2002;32:182-198.
2. Gupta S, Dalal KE, Dandekar A, Nanji R, Aravazhi P, Bomble S, et al. On the Footsteps of Zoroasition Parsis in lndia Excavations of Sanjan on the West Coast 2004.
3. Mushrif-Tripathy V, Walimbe SR. Human Skeletal Remains from the Medieval Site of Sanjan: Osteobiographic Analysis. Archaeopress; 2012.
4. Sambrook J, Fritsch EF, Maniatis T, et al. Molecular cloning: a laboratory manual. Cold Spring Harbor, NY: Cold Spring Harbor Laboratory Press; 1989.
5. Li JZ, Absher DM, Tang H, Southwick AM, Casto AM, Ramachandran S, et al. Worldwide human relationships inferred from genome-wide patterns of variation. Science 2008;319
6. Behar DM, Yunusbayev B, Metspalu M, Metspalu E, Rosset S, Parik J, et al. The genome-wide structure of the Jewish people. Nature 2010;466(7303):238-242.
7. Yunusbayev B, Metspalu M, Järve M, Kutuev I, Rootsi S, Metspalu E, et al. The Caucasus as an asymmetric semipermeable barrier to ancient human migrations. Mol Biol Evol 2012;29(1):359-365.
8. International HapMap 3 Consortium, Altshuler DM, Gibbs RA, Peltonen L, Dermitzakis E, Schaffner SF, et al. Integrating common and rare genetic variation in diverse human populations. Nature 2010;467(7311):52-58.
9. Metspalu M, Romero IG, Yunusbayev B, Chaubey G, Mallick CB, Hudjashov G, et al. Shared and unique components of human population structure and genome-wide signals of positive selection in South Asia. Am J Hum Genet 2011;89(6):731-744.
10. Chaubey G, Metspalu M, Choi Y, Mägi R, Romero IG, Soares P, et al. Population Genetic Structure in Indian Austroasiatic speakers: The Role of Landscape Barriers and Sex-specific Admixture. Mol Biol Evol 2011;28(2):1013-1024.
11. Raghavan M, Skoglund P, Graf KE, Metspalu M, Albrechtsen A, Moltke I, et al. Upper Palaeolithic Siberian genome reveals dual ancestry of Native Americans. Nature 2014;505(7481):87-91.
12. van Oven M, Kayser M. Updated comprehensive phylogenetic tree of global human mitochondrial DNA variation. Hum Mutat 2009;30(2):E386-E394.
13. Chang CC, Chow CC, Tellier LC, Vattikuti S, Purcell SM, Lee JJ, et al. Second-generation PLINK: rising to the challenge of larger and richer datasets. BMC Biol 2015;4(1):1-16.
14. Lazaridis I, Nadel D, Rollefson G, Merrett DC, Rohland N, Mallick S, et al. Genomic insights into the origin of farming in the ancient Near East. Nature 2016;
15. Cockerham CC, Weir BS. Covariances of relatives stemming from a population undergoing mixed self and random mating. Biometrics 1984;40(1):157-164.

16. Patterson N, Price AL, Reich D. Population structure and eigenanalysis. PLoS Genet 2006;2(12):e190.
17. Chaubey G, Kadian A, Bala S, Rao VR. Genetic Affinity of the Bhil, Kol and Gond Mentioned in Epic Ramayana. PloS one 2015;10(6):e0127655.
18. Patterson N, Moorjani P, Luo Y, Mallick S, Rohland N, Zhan Y, et al. Ancient admixture in human history. Genetics 2012;192(3):1065-1093.
19. Chaubey G, Singh M, Rai N, Kariappa M, Singh K, Singh A, et al. Genetic affinities of the Jewish populations of India. Scientific reports 2016;6:19166.
20. Pickrell JK, Pritchard JK. Inference of population splits and mixtures from genome-wide allele frequency data. PLoS Genet 2012;8(11):e1002967.
21. Lawson DJ, Hellenthal G, Myers S, Falush D. Inference of population structure using dense haplotype data. PLoS Genet 2012;8(1):e1002453.
22. Browning BL, Yu Z. Simultaneous genotype calling and haplotype phasing improves genotype accuracy and reduces false-positive associations for genome-wide association studies. Am J Hum Genet 2009;85(6):847-861.
23. Loh PR, Lipson M, Patterson N, Moorjani P, Pickrell JK, Reich D, et al. Inferring admixture histories of human populations using linkage disequilibrium. Genetics 2013;193(4):1233-1254.
24. Pickrell JK, Patterson N, Loh PR, Lipson M, Berger B, Stoneking M, et al. Ancient west Eurasian ancestry in southern and eastern Africa. Proc Natl Acad Sci U S A 2014;111(7):2632-2637.
25. Yi X, Liang Y, Huerta-Sanchez E, Jin X, Cuo ZXP, Pool JE, et al. Sequencing of 50 human exomes reveals adaptation to high altitude. Science 2010;329(5987):75-78.
26. Metspalu M, Kivisild T, Metspalu E, Parik J, Hudjashov G, Kaldma K, et al. Most of the extant mtDNA boundaries in south and southwest Asia were likely shaped during the initial settlement of Eurasia by anatomically modern humans. BMC Genet 2004;5:26.
27. Kivisild T, Rootsi S, Metspalu M, Mastana S, Kaldma K, Parik J, et al. The genetic heritage of the earliest settlers persists both in Indian tribal and caste populations. Am J Hum Genet 2003;72(2):313-332.
28. Chaubey G, Metspalu M, Kivisild T, Villems R. Peopling of South Asia: investigating the caste-tribe continuum in India. Bioessays 2007;29(1):91-100.
29. Cordaux R, Aunger R, Bentley G, Nasidze I, Sirajuddin SM, Stoneking M, et al. Independent origins of Indian caste and tribal paternal lineages. Curr Biol 2004;14(3):231-235.
30. Sahoo S, Singh A, Himabindu G, Banerjee J, Sitalaximi T, Gaikwad S, et al. A prehistory of Indian Y chromosomes: evaluating demic diffusion scenarios. Proc Natl Acad Sci U S A 2006;103(4):843-848.

31. Sengupta S, Zhivotovsky LA, King R, Mehdi SQ, Edmonds CA, Chow CE, et al. Polarity and temporality of high-resolution y-chromosome distributions in India identify both indigenous and exogenous expansions and reveal minor genetic influence of Central Asian pastoralists. Am J Hum Genet 2006;78(2):202-221.
32. Xing J, Watkins WS, Witherspoon DJ, Zhang Y, Guthery SL, Thara R, et al. Fine-scaled human genetic structure revealed by SNP microarrays. Genome Res 2009;19(5):815-825.
33. Reich D, Thangaraj K, Patterson N, Price AL, Singh L. Reconstructing Indian population history. Nature 2009;461(7263):489-494.
34. Moorjani P, Thangaraj K, Patterson N, Lipson M, Loh PR, Govindaraj P, et al. Genetic evidence for recent population mixture in India. Am J Hum Genet 2013;93(3):422-438.
35. Jones ER, Gonzalez-Fortes G, Connell S, Siska V, Eriksson A, Martiniano R, et al. Upper Palaeolithic genomes reveal deep roots of modern Eurasians. Nature communications 2015;6:8912.
36. Broushaki F, Thomas MG, Link V, López S, van Dorp L, Kirsanow K, et al. Early Neolithic genomes from the eastern Fertile Crescent. Science 2016;
37. Haak W, Lazaridis I, Patterson N, Rohland N, Mallick S, Llamas B, et al. Massive migration from the steppe was a source for Indo-European languages in Europe. Nature 2015;
38. Witzel M. Central Asian roots and acculturation in Indian subcontinent: linguistic and archaeological evidence from Western Central Asia, the Hindukush and northwestern Indian subcontinent for early Indo-Aryan language and religion. In: Osada, T, editor. Liguistics, Archaeology and the Human Past.. Koyto, Japan: Research Institute for Humanity and Nature, Koyto; 2005. p. 87-211.
39. Renfrew C. The coming of the Aryans to Iran and India and the cultural and ethnic identity of the Dasas. By Asko Parpola. (Studia Orientalia, Vol. 64.) pp. 195?302, 33 figs. Helsinki, The Finnish Oriental Society, 1988. Journal of the Royal Asiatic Society (Third Series) 1991;1(01):106-109.
40. Lal B. The Earliest Civilization of South Asia: Rise, Maturity, and Decline. Aryan Books International; 1997.
41. Haber M, Gauguier D, Youhanna S, Patterson N, Moorjani P, Botigué LR, et al. Genome-Wide Diversity in the Levant Reveals Recent Structuring by Culture. PLOS Genetics 2013;9(2):e1003316-e1003316.

Fig. S1

a

	Middle East	Iranian	Europe	Caucasus_North	Caucasus_South	Central Asia	Pakistan_North	Pakistan_South	Parsi_Pakistan	Parsi_India	Gujarati	India_IE	India_DRA
Middle East		0.0022	0.0091	0.0058	0.0030	0.0166	0.0169	0.0115	0.0138	0.0152	0.0269	0.0302	0.0366
Iranian	0.0022		0.0098	0.0034	0.0020	0.0117	0.0104	0.0057	0.0106	0.0119	0.0200	0.0232	0.0296
Europe	0.0091	0.0098		0.0075	0.0076	0.0193	0.0210	0.0187	0.0209	0.0224	0.0329	0.0356	0.0437
Caucasus_North	0.0058	0.0034	0.0075		0.0019	0.0089	0.0106	0.0086	0.0128	0.0143	0.0211	0.0237	0.0308
Caucasus_South	0.003	0.0020	0.0076	0.0019		0.0142	0.0136	0.0097	0.0127	0.0143	0.0245	0.0280	0.0351
Central Asia	0.0166	0.0117	0.0193	0.0089	0.0142		0.0090	0.0101	0.0183	0.0191	0.0142	0.0143	0.0189
Pakistan_North	0.0169	0.0104	0.0210	0.0106	0.0136	0.0090		0.0060	0.0158	0.0166	0.0097	0.0108	0.0160
Pakistan_South	0.0115	0.0057	0.0187	0.0086	0.0097	0.0101	0.0060		0.0123	0.0131	0.0099	0.0122	0.0170
Parsi_Pakistan	0.0138	0.0106	0.0209	0.0128	0.0127	0.0183	0.0158	0.0123		0.0003	0.0222	0.0246	0.0303
Parsi_India	0.0152	0.0119	0.0224	0.0143	0.0143	0.0191	0.0166	0.0131	0.0003		0.0228	0.0250	0.0303
Gujarati	0.0269	0.0200	0.0329	0.0211	0.0245	0.0142	0.0097	0.0099	0.0222	0.0228		0.0034	0.0061
India_IE	0.0302	0.0232	0.0356	0.0237	0.0280	0.0143	0.0108	0.0122	0.0246	0.0250	0.0034		0.0031
India_DRA	0.0366	0.0296	0.0437	0.0308	0.0351	0.0189	0.0160	0.0170	0.0303	0.0303	0.0061	0.0031	

0.0003 0.0365

b

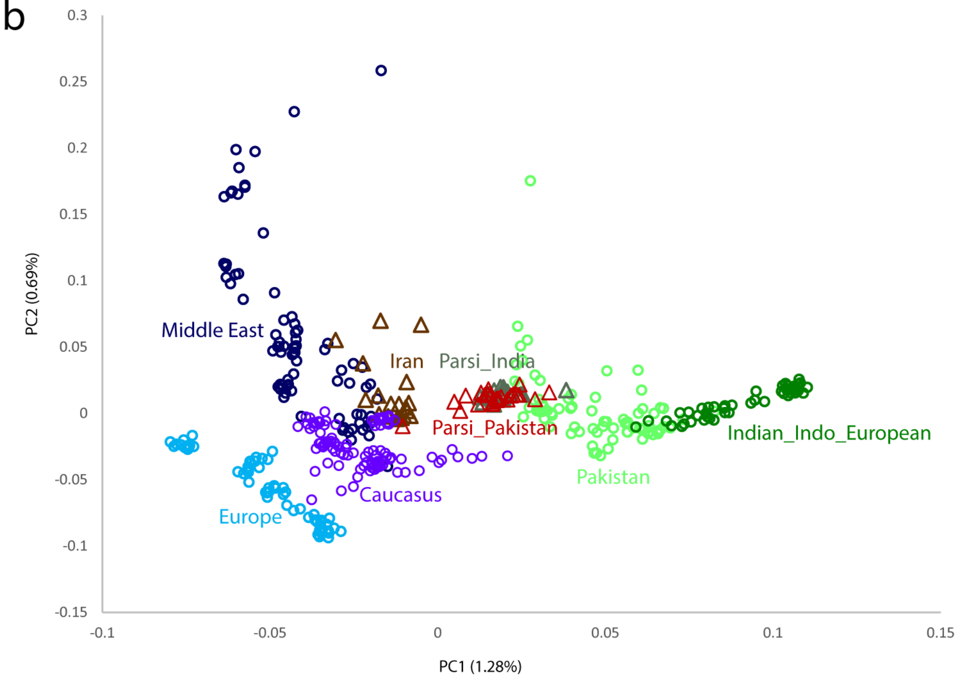

Fig. S2

a

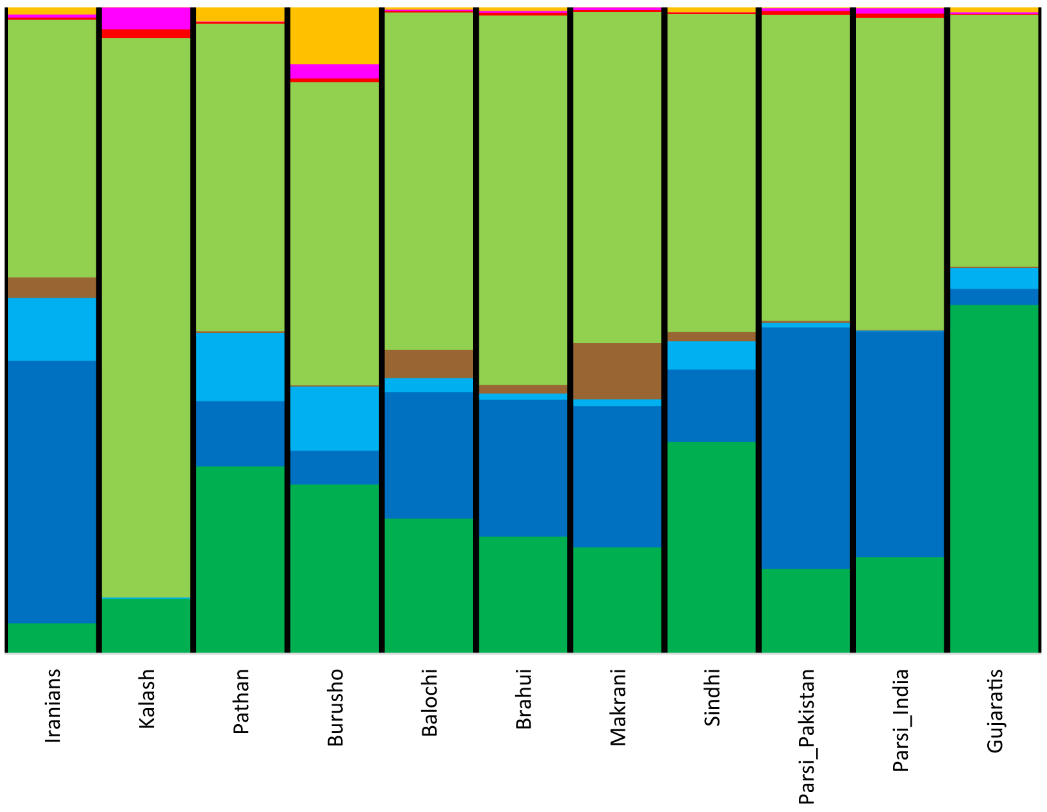
Iranians
Kalash
Pathan
Burusho
Balochi
Brahui
Makrani
Sindhi
Parsi_Pakistan
Parsi_India
Gujaratis

b

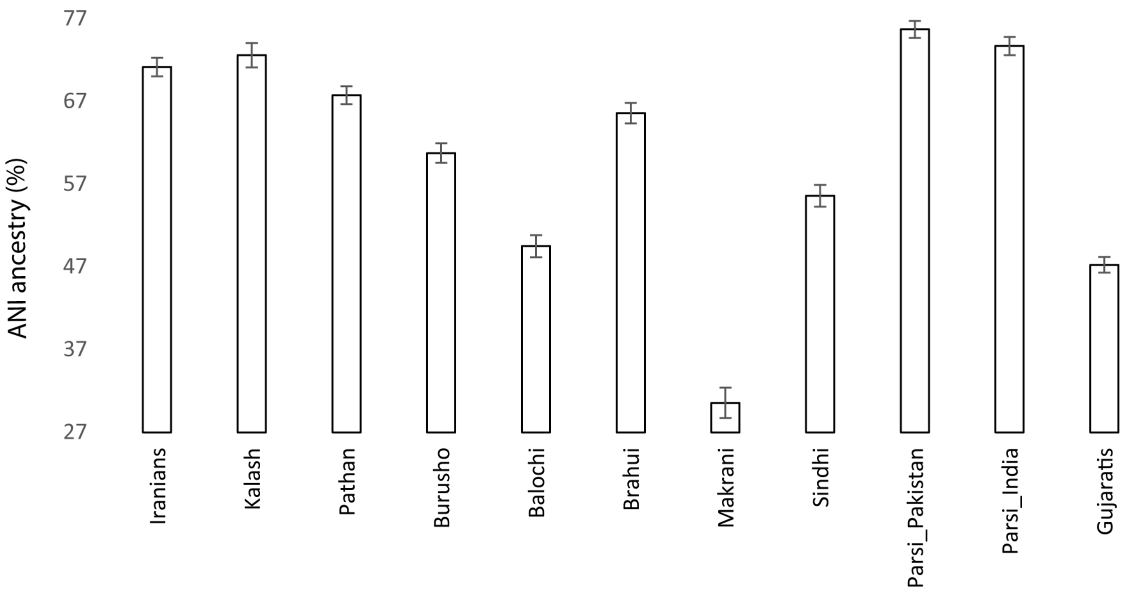
ANI ancestry (%)
77
67
57
47
37
27
Iranians
Kalash
Pathan
Burusho
Balochi
Brahui
Makrani
Sindhi
Parsi_Pakistan
Parsi_India
Gujaratis

Fig. S3

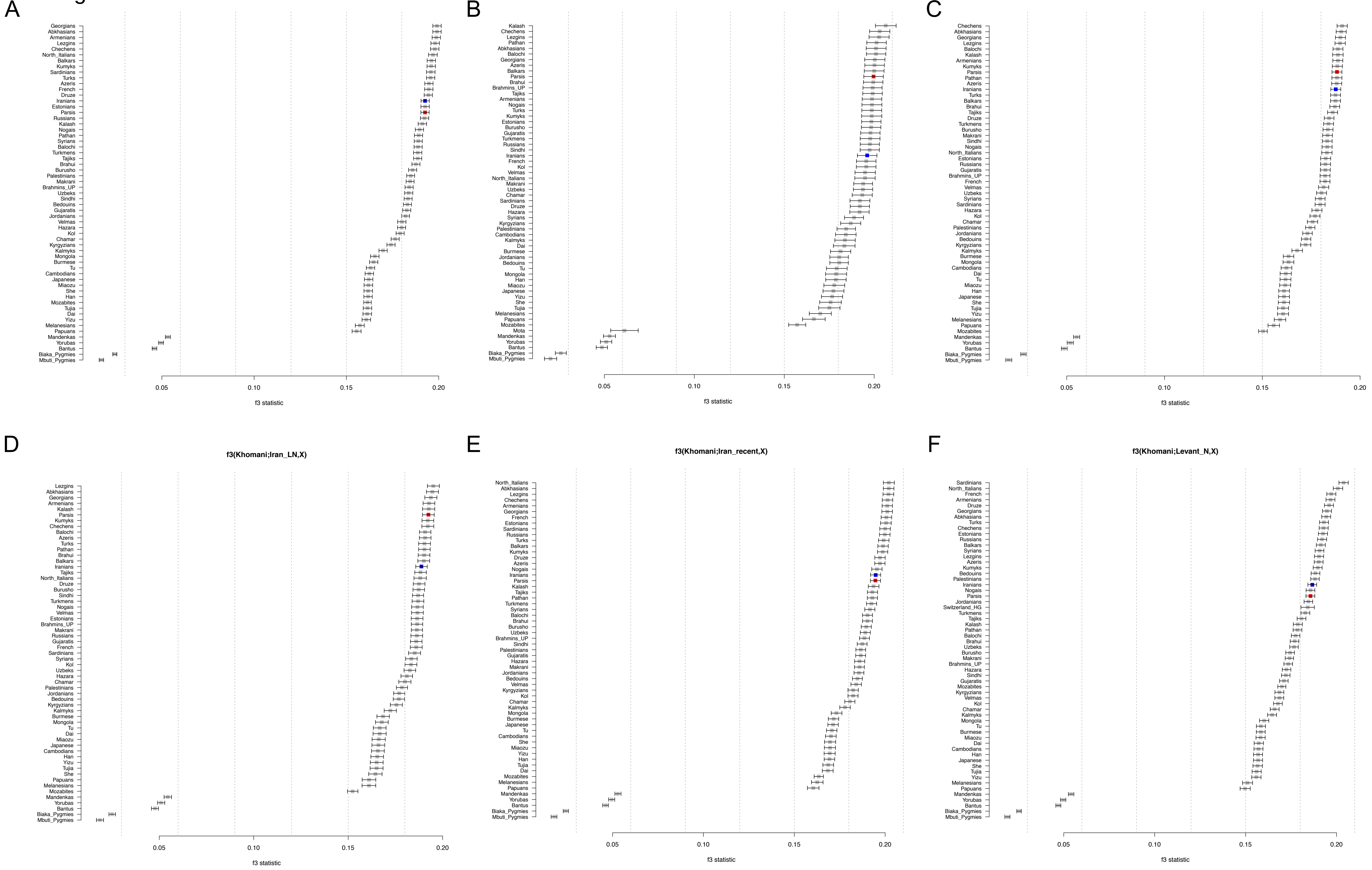

A
f3(Khomani;Iran_ChL,X)
f3 statistic
0.05
0.10
0.15
0.20
B
f3(Khomani;Iran_HotuIIIb,X)
f3 statistic
C
f3(Khomani;Iran_N,X)
f3 statistic
D
f3(Khomani;Iran_LN,X)
f3 statistic
E
f3(Khomani;Iran_recent,X)
f3 statistic
F
f3(Khomani;Levant_N,X)
f3 statistic

Fig. S4

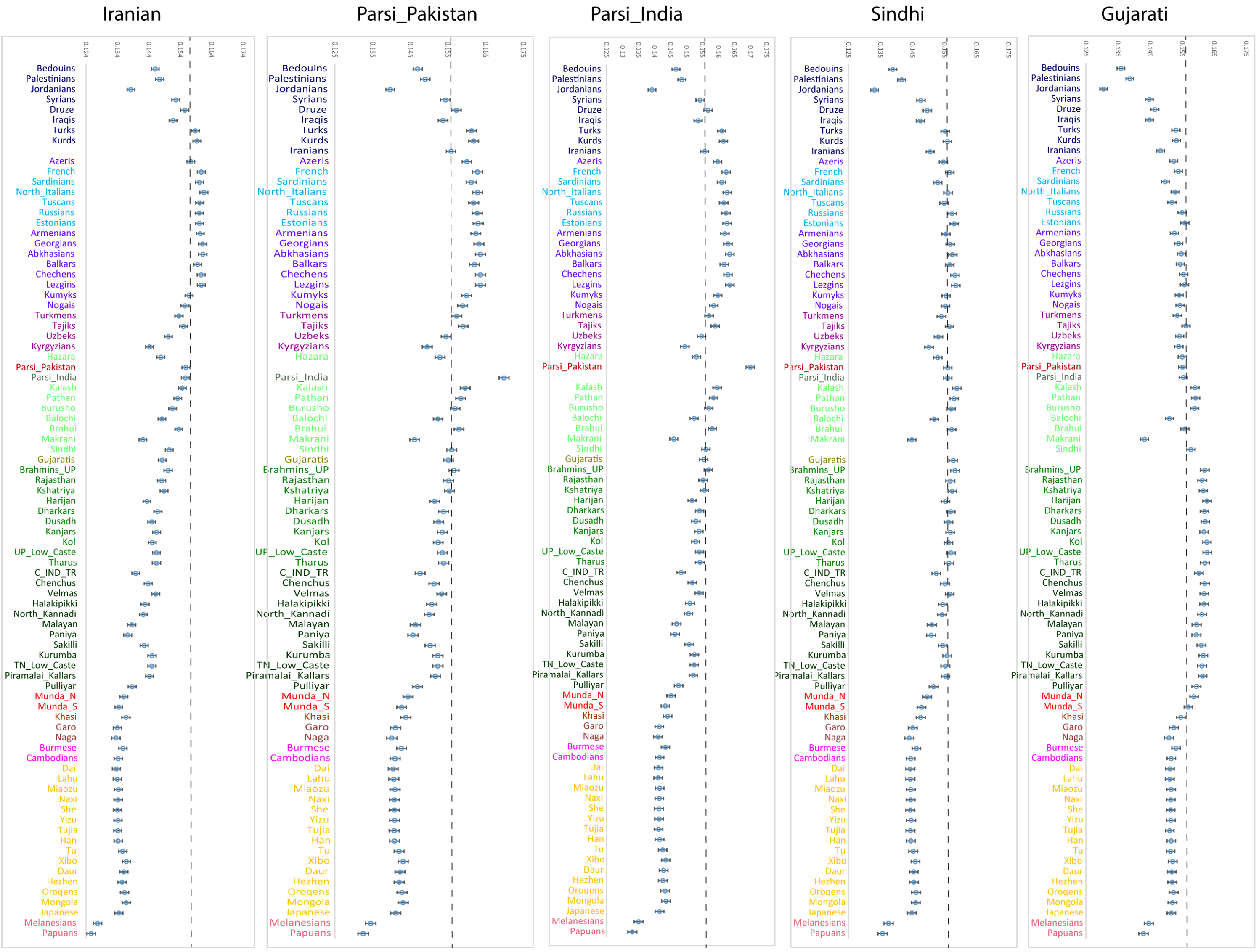
Iranian
Parsi_Pakistan
Parsi_India
Sindhi
Gujarati
Bedouins
Palestinians
Jordanians
Syrians
Druze
Iraqis
Turks
Kurds
Azeris
French
Sardinians
North_Italians
Tuscans
Russians
Estonians
Armenians
Georgians
Abkhasians
Balkars
Chechens
Lezgins
Kumyks
Nogais
Turkmens
Tajiks
Uzbeks
Kyrgyzians
Hazara
Parsi_Pakistan
Parsi_India
Kalash
Pathan
Burusho
Balochi
Brahui
Makrani
Sindhi
Gujaratis
Brahmins_UP
Rajasthan
Kshatriya
Harijan
Dharkars
Dusadh
Kanjars
Kol
UP_Low_Caste
Tharus
C_IND_TR
Chenchus
Velmas
Halakipikki
North_Kannadi
Malayan
Paniya
Sakilli
Kurumba
TN_Low_Caste
Piramalai_Kallars
Pulliyar
Munda_N
Munda_S
Khasi
Garo
Naga
Burmese
Cambodians
Dai
Lahu
Miaozu
Naxi
She
Yizu
Tujia
Han
Tu
Xibo
Daur
Hezhen
Oroqens
Mongola
Japanese
Melanesians
Papuans

Fig. S5

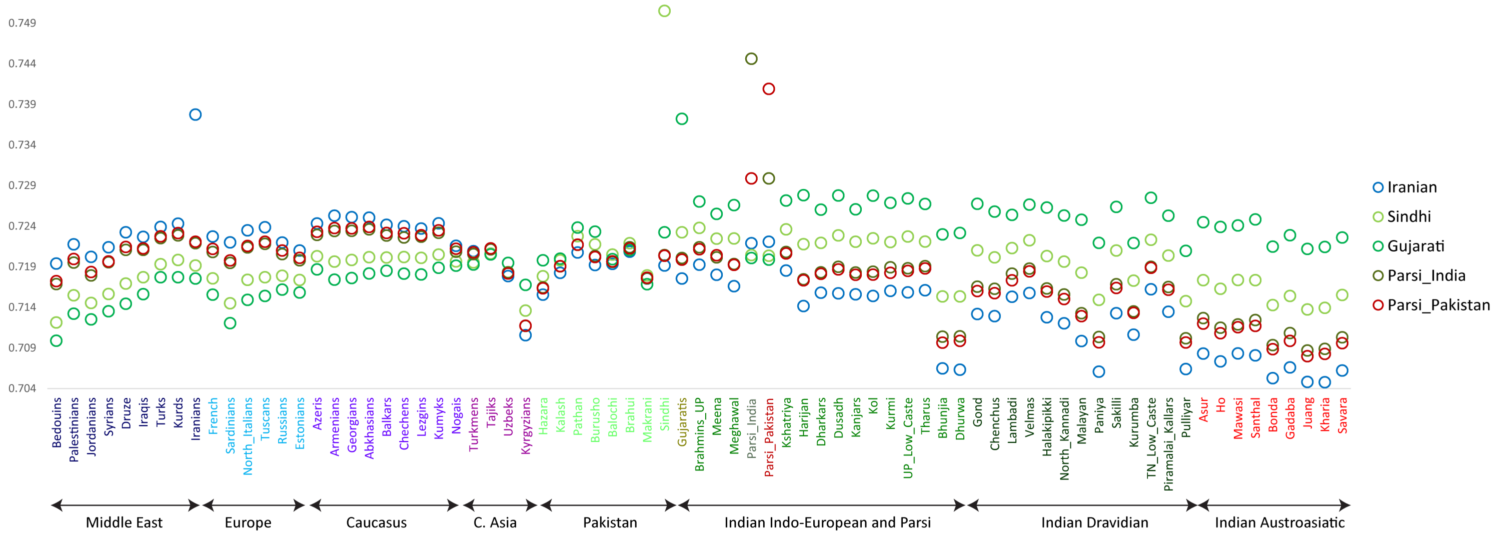
0.749
0.744
0.739
0.734
0.729
0.724
0.719
0.714
0.709
0.704
Iranian
Sindhi
Gujarati
Parsi_India
Parsi_Pakistan
Bedouins
Palestinians
Jordanians
Syrians
Druze
Iraqis
Turks
Kurds
Iranians
French
Sardinians
North_Italians
Tuscans
Russians
Estonians
Azeris
Armenians
Georgians
Abkhasians
Balkars
Chechens
Lezgins
Kumyks
Nogais
Turkmens
Tajiks
Uzbeks
Kyrgyzians
Hazara
Kalash
Pathan
Burusho
Balochi
Brahui
Makrani
Sindhi
Gujaratis
Brahmins_UP
Meena
Meghawal
Parsi_India
Parsi_Pakistan
Kshatriya
Harijan
Dharkars
Dusadh
Kanjars
Kol
Kurmi
UP_Low_Caste
Tharus
Bhunjia
Dhurwa
Gond
Chenchus
Lambadi
Velmas
Halakipikki
North_Kannadi
Malayan
Paniya
Sakilli
Kurumba
TN_Low_Caste
Piramalai_Kallars
Pulliyar
Asur
Ho
Mawasi
Santhal
Bonda
Gadaba
Juang
Kharia
Savara
Middle East
Europe
Caucasus
C. Asia
Pakistan
Indian Indo-European and Parsi
Indian Dravidian
Indian Austroasiatic

Fig. S6

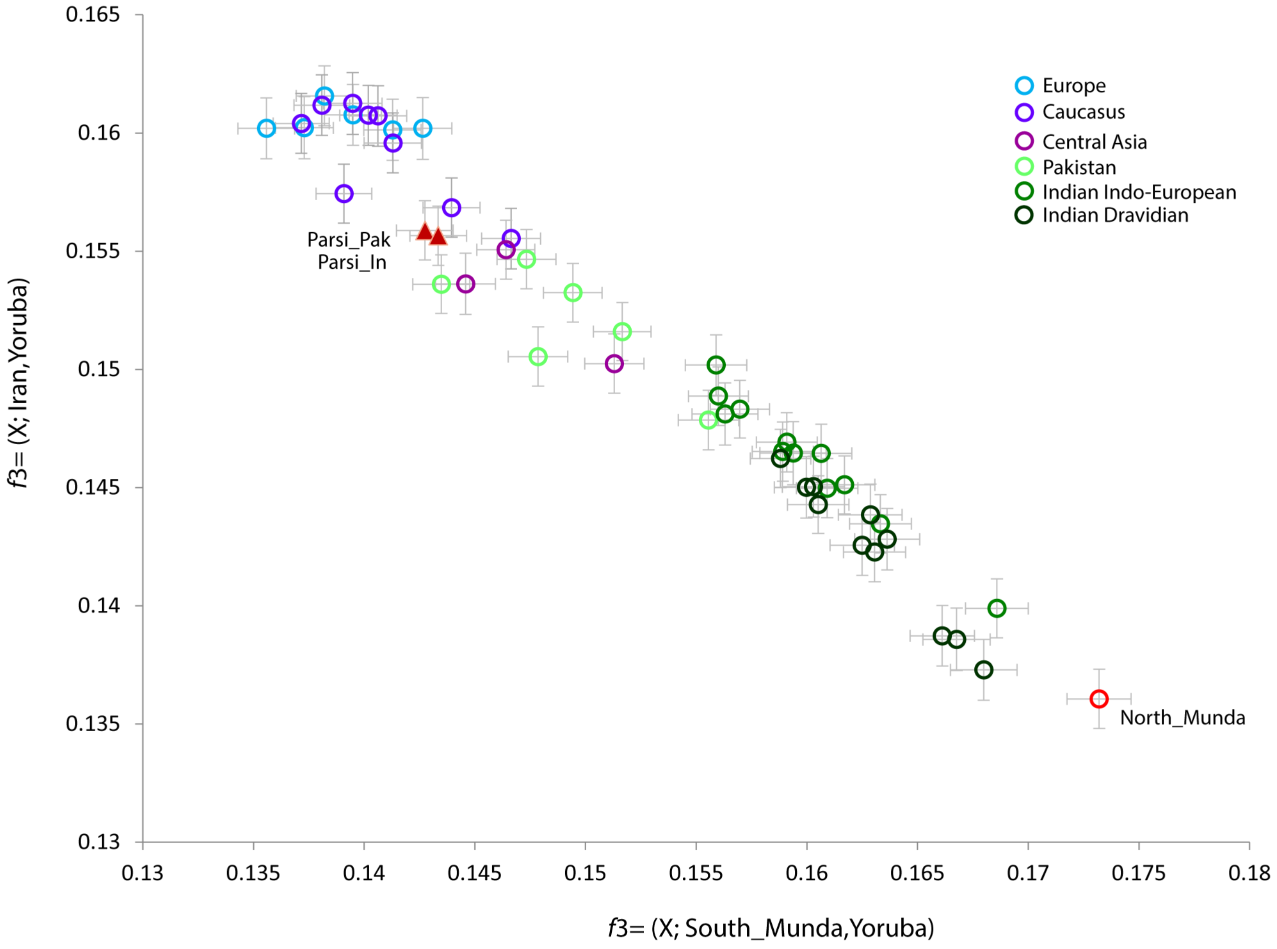

Europe
Caucasus
Central Asia
Pakistan
Indian Indo-European
Indian Dravidian
Parsi_Pak
Parsi_In
North_Munda
0.165
0.16
0.155
0.15
0.145
0.14
0.135
0.13
0.13
0.135
0.14
0.145
0.15
0.155
0.16
0.165
0.17
0.175
0.18
f3= (X; Iran,Yoruba)
f3= (X; South_Munda,Yoruba)

Fig. S7

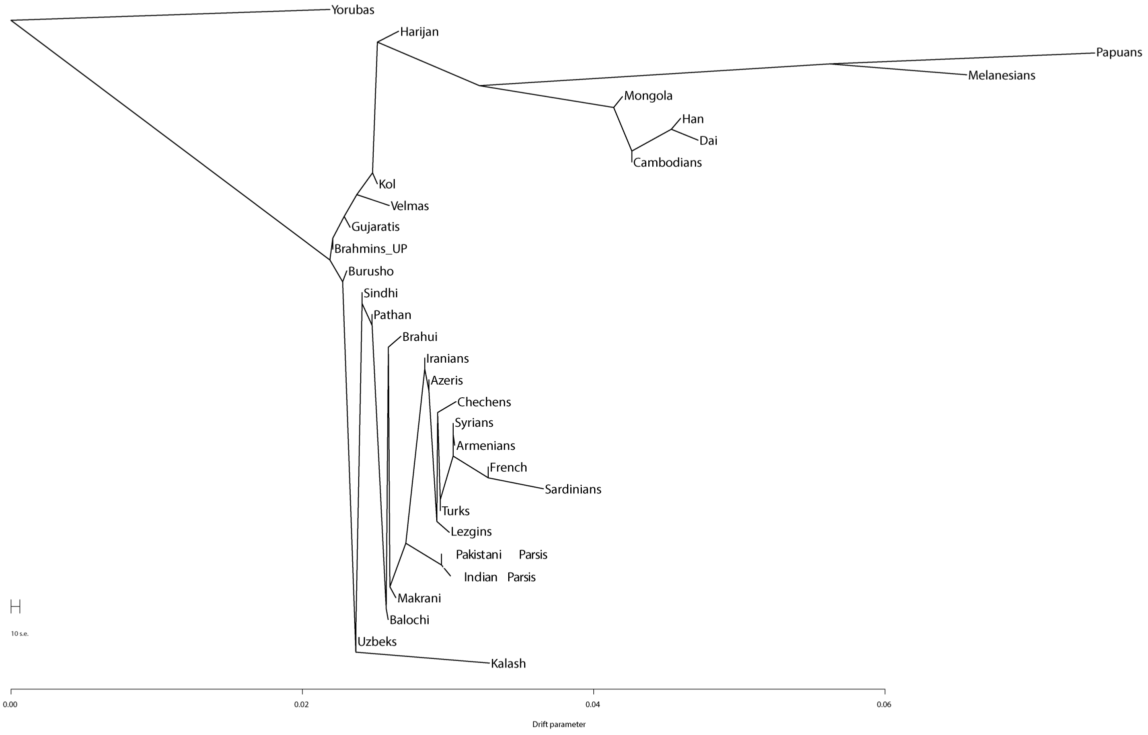

Yorubas
Harijan
Papuans
Melanesians
Mongola
Han
Dai
Cambodians
Kol
Velmas
Gujaratis
Brahmins_UP
Burusho
Sindhi
Pathan
Brahui
Iranians
Azeris
Chechens
Syrians
Armenians
French
Sardinians
Turks
Lezgins
Pakistani Parsis
Indian Parsis
Makrani
Balochi
Uzbeks
Kalash
10 s.e.
0.00
0.02
0.04
0.06
Drift parameter

Fig. S8

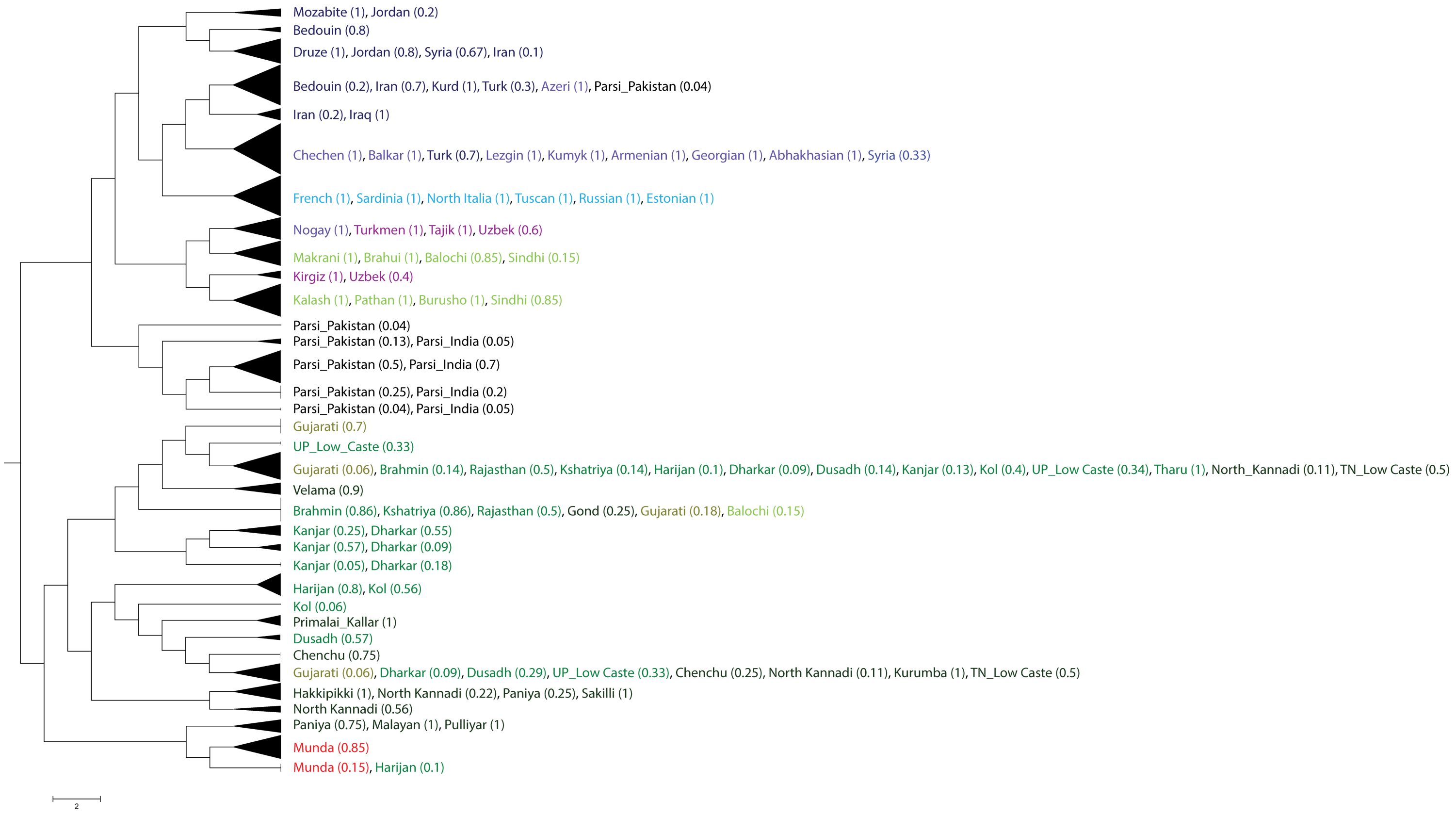
Mozabite (1), Jordan (0.2)
Bedouin (0.8)
Druze (1), Jordan (0.8), Syria (0.67), Iran (0.1)
Bedouin (0.2), Iran (0.7), Kurd (1), Turk (0.3), Azeri (1), Parsi_Pakistan (0.04)
Iran (0.2), Iraq (1)
Chechen (1), Balkar (1), Turk (0.7), Lezgin (1), Kumyk (1), Armenian (1), Georgian (1), Abhakhasian (1), Syria (0.33)
French (1), Sardinia (1), North Italia (1), Tuscan (1), Russian (1), Estonian (1)
Nogay (1), Turkmen (1), Tajik (1), Uzbek (0.6)
Makrani (1), Brahui (1), Balochi (0.85), Sindhi (0.15)
Kirgiz (1), Uzbek (0.4)
Kalash (1), Pathan (1), Burusho (1), Sindhi (0.85)
Parsi_Pakistan (0.04)
Parsi_Pakistan (0.13), Parsi_India (0.05)
Parsi_Pakistan (0.5), Parsi_India (0.7)
Parsi_Pakistan (0.25), Parsi_India (0.2)
Parsi_Pakistan (0.04), Parsi_India (0.05)
Gujarati (0.7)
UP_Low_Caste (0.33)
Gujarati (0.06), Brahmin (0.14), Rajasthan (0.5), Kshatriya (0.14), Harijan (0.1), Dharkar (0.09), Dusadh (0.14), Kanjar (0.13), Kol (0.4), UP_Low Caste (0.34), Tharu (1), North_Kannadi (0.11), TN_Low Caste (0.5)
Velama (0.9)
Brahmin (0.86), Kshatriya (0.86), Rajasthan (0.5), Gond (0.25), Gujarati (0.18), Balochi (0.15)
Kanjar (0.25), Dharkar (0.55)
Kanjar (0.57), Dharkar (0.09)
Kanjar (0.05), Dharkar (0.18)
Harijan (0.8), Kol (0.56)
Kol (0.06)
Primalai_Kallar (1)
Dusadh (0.57)
Chenchu (0.75)
Gujarati (0.06), Dharkar (0.09), Dusadh (0.29), UP_Low Caste (0.33), Chenchu (0.25), North Kannadi (0.11), Kurumba (1), TN_Low Caste (0.5)
Hakkipikki (1), North Kannadi (0.22), Paniya (0.25), Sakilli (1)
North Kannadi (0.56)
Paniya (0.75), Malayan (1), Pulliyar (1)
Munda (0.85)
Munda (0.15), Harijan (0.1)
2

Fig. S9

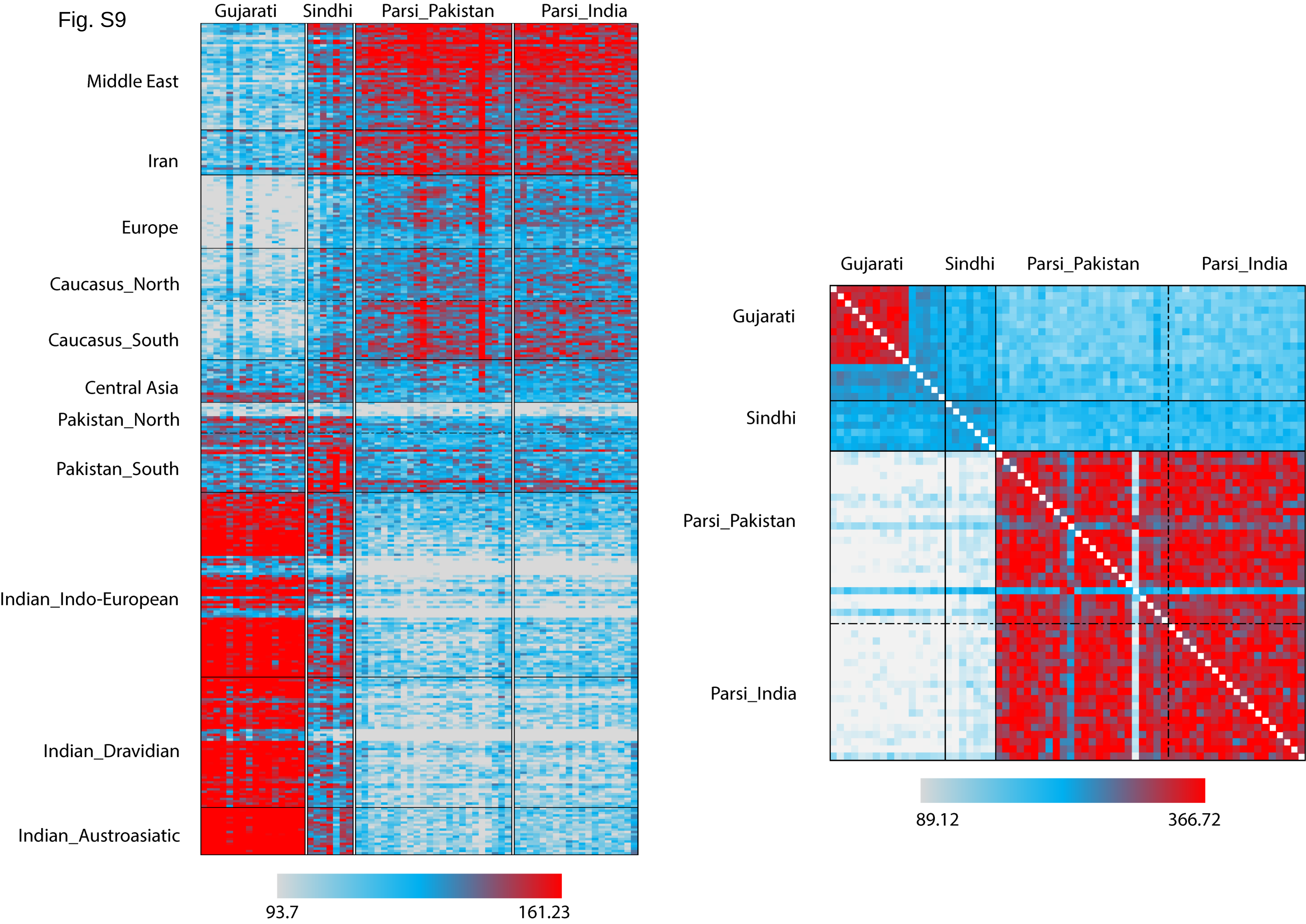
Gujarati
Sindhi
Parsi_Pakistan
Parsi_India
Middle East
Iran
Europe
Caucasus_North
Caucasus_South
Central Asia
Pakistan_North
Pakistan_South
Indian_Indo-European
Indian_Dravidian
Indian_Austroasiatic
93.7
161.23
Gujarati
Sindhi
Parsi_Pakistan
Parsi_India
Gujarati
Sindhi
Parsi_Pakistan
Parsi_India
89.12
366.72

Fig. S10

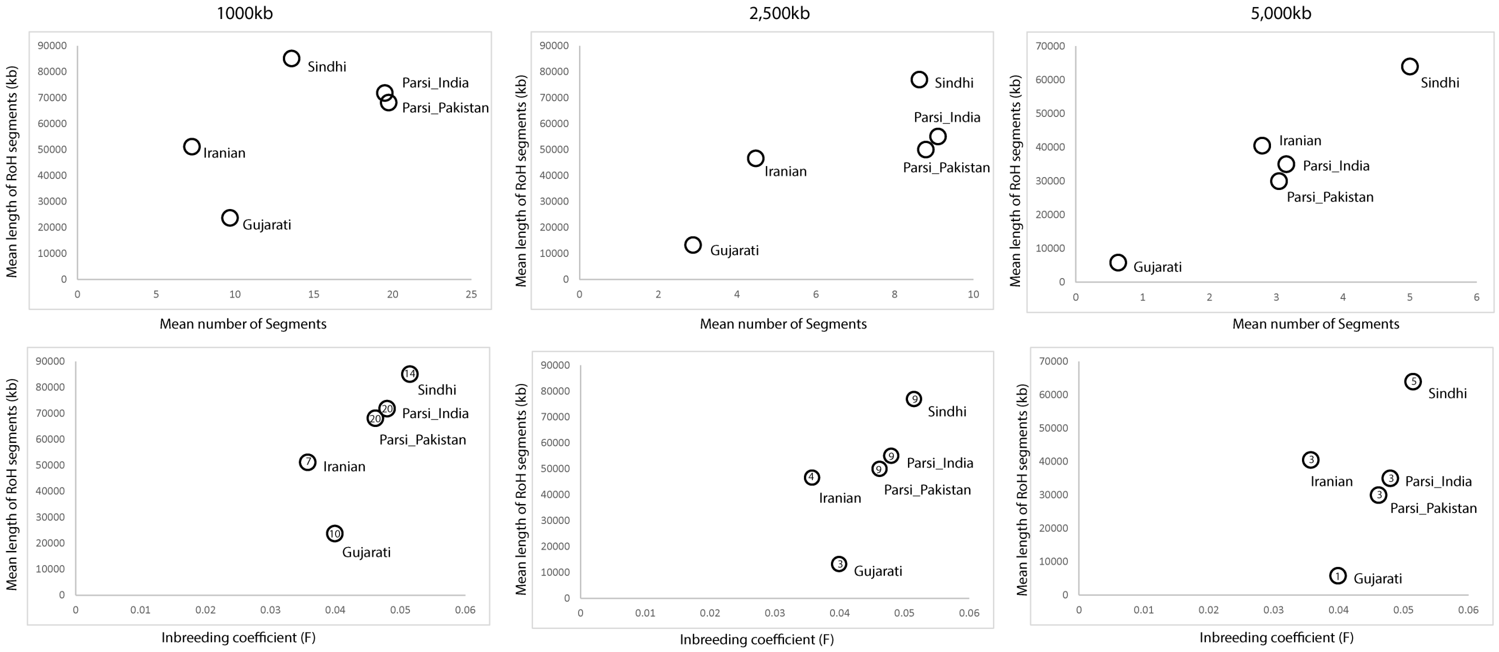

1000kb
2,500kb
5,000kb
Mean length of RoH segments (kb)
Mean number of Segments
Inbreeding coefficient (F)
Sindhi
Parsi_India
Parsi_Pakistan
Iranian
Gujarati

Fig. S11

A

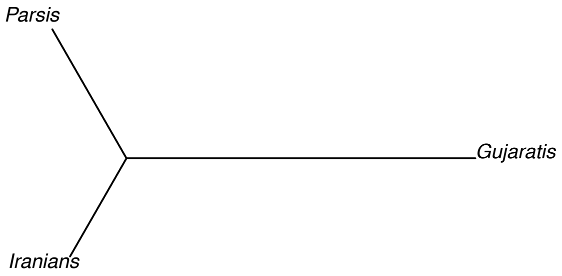
Parsis
Gujaratis
Iranians

B

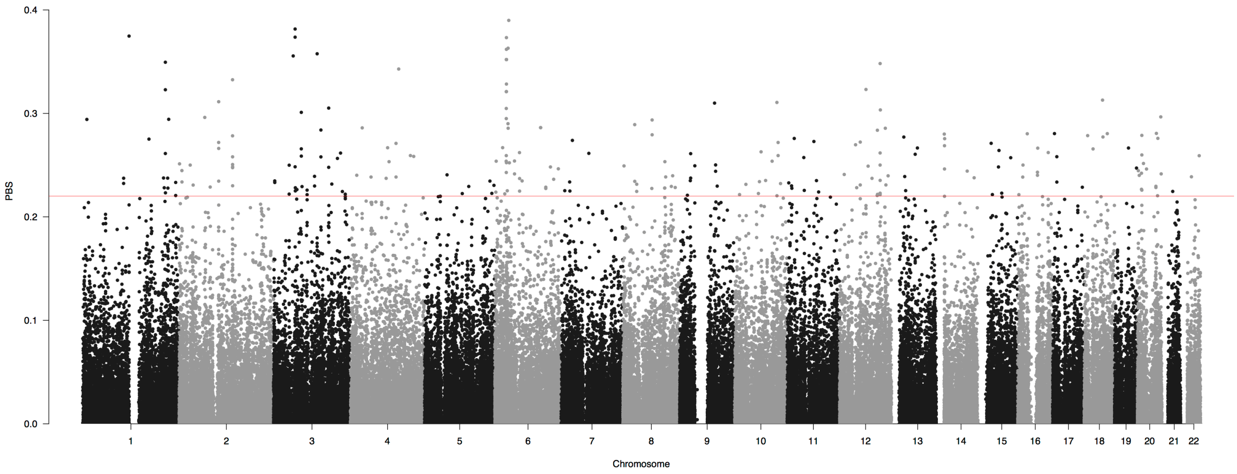
0.4
0.3
0.2
0.1
0.0
PBS
1
2
3
4
5
6
7
8
9
10
11
12
13
14
15
16
17
18
19
20
21
22
Chromosome

C

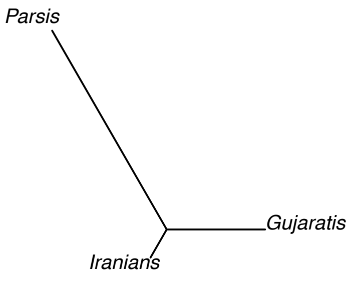
Parsis
Gujaratis
Iranians

Fig. S12

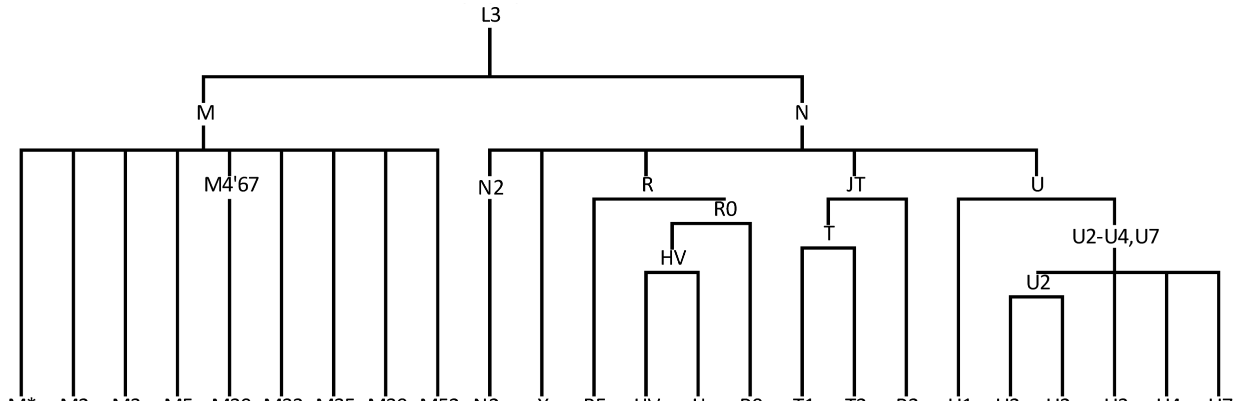

	N	M*	M2	M3	M5	M30	M33	M35	M39	M52	N2a	X	R5	HV	H	R0	T1	T2	R2	U1	U2c	U2e	U3	U4	U7
Parsi_India	117	0.01	0.03	0.25	0.03	0.15	0.02	0.09	0.01	0.03	-	-	-	0.06	0.08	0.01	0.03	0.03	-	-	0.02	0.02	0.02	0.09	0.04
Parsi_India (aDNA)	21	-	0.05	0.29	0.10	-	-	-	-	-	-	0.10	0.05	0.05	-	0.05	0.05	0.05	0.05	0.05	-	-	-	-	0.14
Parsi_Pakistan	50	-	-	0.12	0.02	0.14	-	-	0.12	0.08	0.02	-	-	0.14	-	0.04	0.06	0.04	-	0.06	-	-	-	0.10	0.06

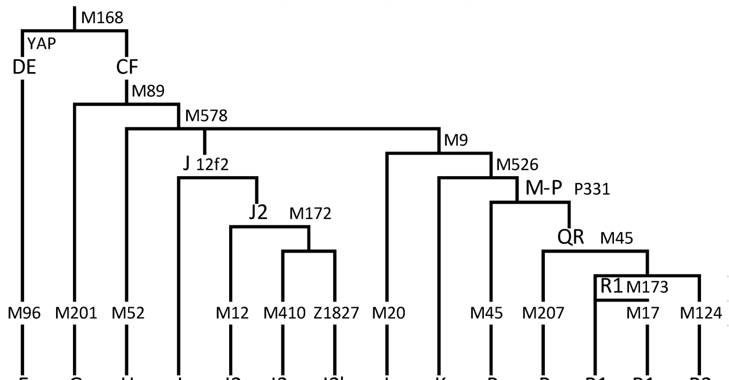

Population	*N*	E	G	H	J	J2	J2a	J2b	L	K	P	R	R1	R1a	R2
Parsi_India	84	0.06	0.02	0.01	0.01	0.04	0.30	0.02	0.21	0.01	0.04	-	0.04	0.18	0.06
Parsi_Pakistan	90	0.06	0.01	0.02	-	-	0.39	-	0.18	-	-	0.01	0.04	0.08	0.21

Table S1. The arrival and expansion of Parsis in Indian subcontinent. The details have been obtained from Parzor foundation, New Delhi, India.

Region	Current population	Founding time	Purpose of migration
Delhi (India)	250-300	16th Century	Clock-keepers in Mughal courts
Kolkata (India)	600	1839	Opium and cotton trade
Sanjan/Surat (India)	3584	7th Century	Fled from Iran
Bombay (India)	40,000	1640	Ship builder
Dahanu (India)	180	19th Century	Pioneered Chikoo farming
Hyderabad (India)	1136	19th Century	Court of Nizam
Ooty/Coonoor (India)	30	19th Century	Tea plantation
Karachi (Pakistan)	2000-5000	18th Century	Trade

Table S2. Details of the modern populations, number of samples and SNPs used in various analyses based on autosomal data

Region	Population	N	PCA	ADMIXTURE/Fst	fineSTRUCTURE	$f3$/D	References
Sub Saharan Africa	San	5	-	5	-	5	Li et al. 2008
	Biaka_Pygmies	7	-	7	-	7	Li et al. 2008
	Mbuti_Pygmies	10	-	10	-	10	Li et al. 2008
	Bantus	10	-	10	-	10	Li et al. 2008
	Mandenkas	11	-	11	-	11	Li et al. 2008
	Yorubas	11	-	11	-	11	Li et al. 2008
North Africa and Middle East	Mozabites	10	-	10	-	10	Li et al. 2008
	Bedouins	12	12	12	5	12	Li et al. 2008
	Palestinians	10	10	10	5	10	Li et al. 2008
	Jordanians	8	8	8	5	8	Behar et al. 2010
	Syrians	7	7	7	3	7	Behar et al. 2010
	Druze	10	10	10	5	10	Li et al. 2008
	Iraqis	7	7	7	7	7	Behar et al. 2010
	Turks	9	9	9	7	9	Behar et al. 2010
	Kurds	6	6	6	4	6	Behar et al. 2010
	Iranians	19	19	19	19	19	Behar et al. 2010
Europe	French	12	12	12	6	12	Li et al. 2008
	Sardinians	12	12	12	7	12	Li et al. 2008
	North_Italians	8	8	8	5	8	Li et al. 2008
	Tuscans	7	7	7	4	7	Li et al. 2008
	Russians	11	11	11	4	11	Li et al. 2008
	Estonians	11	11	11	5	11	Raghavan et al. 2014
Caucasus	Azeris	16	16	16	9	16	Yunusbayev et al. 2012
	Armenians	19	19	19	7	19	Behar et al. 2010, Yunusbayev et al. 2012
	Georgians	15	15	15	5	15	Yunusbayev et al. 2012
	Abkhasians	7	7	7	4	7	Yunusbayev et al. 2012
	Balkars	8	8	8	4	8	Yunusbayev et al. 2012
	Chechens	9	9	9	6	9	Yunusbayev et al. 2012
	Lezgins	8	8	8	4	8	Yunusbayev et al. 2012
	Kumyks	7	7	7	3	7	Yunusbayev et al. 2012
	Nogais	10	10	10	5	10	Yunusbayev et al. 2012
Central Asia	Turkmens	5	5	5	3	5	Yunusbayev et al. 2012
	Tajiks	10	10	10	6	10	Yunusbayev et al. 2012
	Uzbeks	9	9	9	5	9	Behar et al. 2010
	Kyrgyzians	5	5	5	4	5	Raghavan et al 2014
Pakistan	Hazara	9	9	9	-	9	Li et al. 2008
	Kalash	9	9	9	6	9	Li et al. 2008
	Pathan	11	11	11	7	11	Li et al. 2008
	Burusho	11	11	11	6	11	Li et al. 2008
	Balochi	11	11	11	6	11	Li et al. 2008
	Brahui	10	10	10	7	10	Li et al. 2008
	Makrani	10	10	10	6	10	Li et al. 2008
	Sindhi	10	10	10	7	10	Li et al. 2008
	Parsi_Pakistan	24	24	24		24	Present study
India	Gujaratis	32	32	32	16	32	HapMap3
	Brahmins_UP	7	7	7	7	7	Metspalu et al. 2011
	Rajasthan	2	2	2	2	2	Metspalu et al. 2011
	Parsi_India	19	19	19	19	19	Present study
	Kshatriya	7	7	7	7	7	Metspalu et al. 2011
	Harijan	10	10	10	10	10	Metspalu et al. 2011
	Dharkars	11	11	11	11	11	Metspalu et al. 2011
	Dusadh	7	7	7	7	7	Metspalu et al. 2011
	Kanjars	8	8	8	8	8	Metspalu et al. 2011
	Kol	16	16	16	16	16	Metspalu et al. 2011
	UP_Low_Caste	5	5	5	5	5	Metspalu et al. 2011
	Tharus	2	2	2	2	2	Metspalu et al. 2011
	Transitional	2	2	2	2	2	Metspalu et al. 2011
	Gond	4	4	4	4	4	Metspalu et al. 2011
	Chenchus	4	4	4	4	4	Metspalu et al. 2011
	Velmas	10	10	10	10	10	Metspalu et al. 2011
	Hakkipikki	4	4	4	4	4	Metspalu et al. 2011
	North_Kannadi	9	9	9	9	9	Behar et al. 2010
	Malayan	2	2	2	2	2	Behar et al. 2010
	Paniya	4	4	4	4	4	Behar et al. 2010
	Sakilli	4	4	4	4	4	Behar et al. 2010
	Kurumba	4	4	4	4	4	Metspalu et al. 2011
	TN_Low_Caste	2	2	2	2	2	Metspalu et al. 2011
	Piramalai_Kallars	8	8	8	8	8	Metspalu et al. 2011
	Pulliyar	5	5	5	5	5	Metspalu et al. 2011
	North Munda	9	9	9	9	9	Chaubey et al. 2011
	South Munda	11	11	11	11	11	Chaubey et al. 2011
	Khasi	3	3	3	-	3	Chaubey et al. 2011
	Garo	4	4	4	-	4	Chaubey et al. 2011
	Naga	4	4	4	-	4	Metspalu et al. 2011
Southeast Asia	Burmese	15	15	15	-	15	Chaubey et al. 2011
	Cambodians	10	10	10	-	10	Li et al. 2008
East Asia	Dai	10	10	10	-	10	Li et al. 2008
	Lahu	8	8	8	-	8	Li et al. 2008
	Miaozu	10	10	10	-	10	Li et al. 2008
	Naxi	8	8	8	-	8	Li et al. 2008
	She	10	10	10	-	10	Li et al. 2008
	Yizu	10	10	10	-	10	Li et al. 2008
	Tujia	10	10	10	-	10	Li et al. 2008
	Han	22	22	22	-	22	Li et al. 2008
	Tu	10	10	10	-	10	Li et al. 2008
	Xibo	9	9	9	-	9	Li et al. 2008
	Daur	9	9	9	-	9	Li et al. 2008
	Hezhen	9	9	9	-	9	Li et al. 2008
	Oroqens	9	9	9	-	9	Li et al. 2008
	Mongola	10	10	10	-	10	Li et al. 2008
	Japanese	11	11	11	-	11	Li et al. 2008
PNG	Melanesians	10	-	10	-	10	Li et al. 2008

	Papuans	17	-	17	-	17	Li et al. 2008
Total number of samples		872	781	872	393	872	
Total number of SNPs		289273	177683	177683	177683	289273	

References:

Li, J. Z. *et al.* Worldwide human relationships inferred from genome-wide patterns of variation. *Science.* **319**, 1100-1104 (2008).

Behar, D. M. *et al.* The genome-wide structure of the Jewish people. *Nature.* **466**, 238-242 (2010).

Yunusbayev, B. *et al.* The Caucasus as an asymmetric semipermeable barrier to ancient human migrations. *Mol Biol Evol.* **29**, 359-365 (2012).

International HapMap 3 Consortium *et al.* Integrating common and rare genetic variation in diverse human populations. *Nature.* **467**, 52-58 (2010).

Metspalu, M. *et al.* Shared and unique components of human population structure and genome-wide signals of positive selection in South Asia. *Am J Hum Genet.* **89**, 731-744 (2011).

Chaubey, G. *et al.* Population Genetic Structure in Indian Austroasiatic speakers: The Role of Landscape Barriers and Sex-specific Admixture. *Mol Biol Evol.* **28**, 1013-1024 (2011).

Raghavan, M. *et al.* Upper Palaeolithic Siberian genome reveals dual ancestry of Native Americans. *Nature.* **505**, 87-91 (2014).

Table S4. The raw values of eight ancestral components used to plot Fig. 3.

San	HGDP00991	0.000015	0.00001	0.00001	0.999924	0.00001	0.00001	0.00001	0.00001
San	HGDP00992	0.000014	0.00001	0.000014	0.999919	0.00001	0.000013	0.00001	0.00001
San	HGDP01029	0.00001	0.00001	0.00001	0.999928	0.00001	0.000012	0.00001	0.00001
San	HGDP01032	0.00001	0.00001	0.00001	0.999927	0.000013	0.00001	0.00001	0.00001
San	HGDP01036	0.000012	0.00001	0.000017	0.999916	0.00001	0.000015	0.00001	0.00001
Biaka_Pygmies	HGDP00461	0.00001	0.00001	0.00001	0.99993	0.00001	0.00001	0.00001	0.00001
Biaka_Pygmies	HGDP00464	0.00001	0.00001	0.00001	0.999927	0.00001	0.000013	0.00001	0.00001
Biaka_Pygmies	HGDP00465	0.00001	0.00001	0.00001	0.99993	0.00001	0.00001	0.00001	0.00001
Biaka_Pygmies	HGDP00466	0.00001	0.00001	0.00001	0.999922	0.00001	0.000018	0.00001	0.00001
Biaka_Pygmies	HGDP00469	0.00001	0.00001	0.00002	0.999916	0.00001	0.000014	0.00001	0.00001
Biaka_Pygmies	HGDP00470	0.000017	0.00001	0.00001	0.999923	0.00001	0.00001	0.00001	0.00001
Biaka_Pygmies	HGDP00472	0.00001	0.00001	0.00001	0.99993	0.00001	0.00001	0.00001	0.00001
Mbuti_Pygmies	HGDP00449	0.00001	0.00001	0.00001	0.99993	0.00001	0.00001	0.00001	0.00001
Mbuti_Pygmies	HGDP00450	0.00001	0.00001	0.00001	0.99993	0.00001	0.00001	0.00001	0.00001
Mbuti_Pygmies	HGDP00456	0.00001	0.00001	0.00001	0.99993	0.00001	0.00001	0.00001	0.00001
Mbuti_Pygmies	HGDP00462	0.00001	0.00001	0.00001	0.99993	0.00001	0.00001	0.00001	0.00001
Mbuti_Pygmies	HGDP00463	0.00001	0.00001	0.00001	0.99993	0.00001	0.00001	0.00001	0.00001
Mbuti_Pygmies	HGDP00467	0.00001	0.00001	0.00001	0.99993	0.00001	0.00001	0.00001	0.00001
Mbuti_Pygmies	HGDP00471	0.00001	0.00001	0.00001	0.99993	0.00001	0.00001	0.00001	0.00001
Mbuti_Pygmies	HGDP00474	0.00001	0.00001	0.00001	0.99993	0.00001	0.00001	0.00001	0.00001
Mbuti_Pygmies	HGDP00476	0.00001	0.00001	0.00001	0.99993	0.00001	0.00001	0.00001	0.00001
Mbuti_Pygmies	HGDP01081	0.00001	0.00001	0.00001	0.99993	0.00001	0.00001	0.00001	0.00001
Bantus	HGDP00993	0.008212	0.011522	0.00001	0.979224	0.00001	0.001002	0.00001	0.00001
Bantus	HGDP00994	0.00001	0.012626	0.00001	0.982659	0.00001	0.004665	0.00001	0.00001
Bantus	HGDP01033	0.007951	0.026085	0.00001	0.964224	0.00001	0.00001	0.00001	0.0017
Bantus	HGDP01030	0.00001	0.00001	0.00001	0.996323	0.00001	0.003617	0.00001	0.00001
Bantus	HGDP01034	0.00001	0.022848	0.00001	0.966936	0.00001	0.003964	0.006212	0.00001
Bantus	HGDP01028	0.013526	0.020902	0.00001	0.959259	0.00001	0.003518	0.002766	0.00001
Bantus	HGDP01035	0.00001	0.03336	0.00001	0.962285	0.00001	0.00001	0.004306	0.00001
Bantus	HGDP01031	0.015385	0.022279	0.00001	0.962286	0.00001	0.00001	0.00001	0.00001
Bantus	HGDP01405	0.014222	0.047932	0.00001	0.932663	0.00001	0.003862	0.001292	0.00001
Bantus	HGDP01408	0.0229	0.054515	0.00001	0.916937	0.00001	0.00001	0.005608	0.00001
Mandenkas	HGDP00904	0.00367	0.046195	0.00001	0.942083	0.00001	0.008013	0.00001	0.00001
Mandenkas	HGDP00905	0.012262	0.053457	0.00001	0.929289	0.00001	0.004952	0.00001	0.00001
Mandenkas	HGDP00906	0.009103	0.058207	0.00001	0.932639	0.00001	0.00001	0.00001	0.00001
Mandenkas	HGDP00907	0.01506	0.043643	0.00001	0.941247	0.00001	0.00001	0.00001	0.00001
Mandenkas	HGDP00908	0.025317	0.037999	0.00001	0.936634	0.00001	0.00001	0.00001	0.00001
Mandenkas	HGDP00909	0.011158	0.04727	0.00001	0.940529	0.00001	0.001003	0.00001	0.00001
Mandenkas	HGDP00910	0.012611	0.05267	0.00001	0.934669	0.00001	0.00001	0.00001	0.00001
Mandenkas	HGDP00911	0.020213	0.051245	0.00001	0.925286	0.00001	0.003217	0.00001	0.00001
Mandenkas	HGDP00912	0.020802	0.040805	0.00001	0.938343	0.00001	0.00001	0.00001	0.00001
Mandenkas	HGDP00913	0.006959	0.048112	0.00001	0.939921	0.00001	0.00001	0.00001	0.004968
Mandenkas	HGDP00915	0.011407	0.063191	0.00001	0.920685	0.00001	0.00001	0.00001	0.004677
Yorubas	HGDP00924	0.014256	0.026938	0.00001	0.958756	0.00001	0.00001	0.00001	0.00001
Yorubas	HGDP00925	0.003884	0.035739	0.00001	0.959534	0.00001	0.00001	0.00001	0.000803
Yorubas	HGDP00926	0.005091	0.034686	0.00001	0.95919	0.00001	0.00001	0.000993	0.00001
Yorubas	HGDP00927	0.01197	0.031723	0.00001	0.956258	0.00001	0.00001	0.00001	0.00001
Yorubas	HGDP00928	0.012242	0.032835	0.000015	0.954868	0.00001	0.00001	0.00001	0.00001
Yorubas	HGDP00929	0.015624	0.023541	0.00001	0.956156	0.00001	0.004639	0.00001	0.00001
Yorubas	HGDP00930	0.019931	0.022624	0.00001	0.955703	0.00001	0.00001	0.001702	0.00001
Yorubas	HGDP00931	0.020522	0.026873	0.00001	0.952555	0.00001	0.00001	0.00001	0.00001
Yorubas	HGDP00932	0.01089	0.025232	0.00001	0.960566	0.00001	0.003272	0.00001	0.00001
Yorubas	HGDP00933	0.008796	0.028278	0.00001	0.962876	0.00001	0.00001	0.00001	0.00001
Yorubas	HGDP00934	0.008076	0.036582	0.00001	0.950692	0.00001	0.004609	0.00001	0.00001
Mozabites	HGDP01253	0.014084	0.598389	0.187687	0.199799	0.00001	0.00001	0.00001	0.00001

Mozabites	HGDP01257	0.012783	0.606857	0.143045	0.23539	0.00001	0.00001	0.00001	0.001894
Mozabites	HGDP01261	0.018724	0.467879	0.150935	0.351645	0.00001	0.004629	0.00001	0.006167
Mozabites	HGDP01264	0.018698	0.582637	0.20351	0.195114	0.00001	0.00001	0.00001	0.00001
Mozabites	HGDP01267	0.024402	0.606782	0.169227	0.197398	0.00001	0.001183	0.00001	0.000989
Mozabites	HGDP01269	0.025651	0.614647	0.148311	0.211351	0.00001	0.00001	0.00001	0.00001
Mozabites	HGDP01274	0.005224	0.540611	0.172696	0.268255	0.00001	0.002106	0.00001	0.011088
Mozabites	HGDP01275	0.008988	0.600709	0.191126	0.19528	0.00001	0.000432	0.00001	0.003446
Mozabites	HGDP01278	0.009261	0.58322	0.208217	0.196823	0.00001	0.00001	0.00001	0.002448
Mozabites	HGDP01279	0.024841	0.598426	0.17712	0.199539	0.00001	0.00001	0.000043	0.00001
Bedouins	HGDP00608	0.02618	0.813663	0.00001	0.055511	0.096379	0.00001	0.00001	0.008237
Bedouins	HGDP00612	0.013595	0.957722	0.00001	0.028634	0.00001	0.00001	0.00001	0.00001
Bedouins	HGDP00616	0.024683	0.953759	0.00001	0.021508	0.00001	0.00001	0.00001	0.00001
Bedouins	HGDP00622	0.014973	0.62611	0.099529	0.08039	0.170153	0.007902	0.000933	0.00001
Bedouins	HGDP00624	0.028595	0.890504	0.00001	0.059701	0.021155	0.000015	0.00001	0.00001
Bedouins	HGDP00627	0.039935	0.825563	0.00001	0.121176	0.008913	0.004382	0.00001	0.00001
Bedouins	HGDP00631	0.020281	0.94738	0.00001	0.032287	0.00001	0.000011	0.00001	0.00001
Bedouins	HGDP00635	0.001302	0.579304	0.098875	0.102244	0.207094	0.000013	0.011156	0.00001
Bedouins	HGDP00638	0.017109	0.618591	0.098898	0.126329	0.134943	0.004109	0.00001	0.00001
Bedouins	HGDP00643	0.029424	0.940107	0.00001	0.02887	0.00001	0.00001	0.00001	0.001559
Bedouins	HGDP00648	0.024519	0.906644	0.00001	0.042186	0.012443	0.00001	0.00001	0.014178
Bedouins	HGDP00650	0.029909	0.918431	0.00001	0.038655	0.000786	0.00001	0.00001	0.012188
Palestinians	HGDP00676	0.011648	0.587547	0.111078	0.058423	0.225048	0.004385	0.00001	0.001861
Palestinians	HGDP00680	0.021467	0.58388	0.10341	0.093418	0.194908	0.002896	0.00001	0.00001
Palestinians	HGDP00684	0.010735	0.618382	0.100521	0.063752	0.193308	0.006135	0.005784	0.001384
Palestinians	HGDP00693	0.003654	0.595457	0.118906	0.061188	0.216772	0.00001	0.00001	0.004004
Palestinians	HGDP00698	0.01564	0.577343	0.138478	0.070254	0.198254	0.00001	0.00001	0.00001
Palestinians	HGDP00724	0.013309	0.585697	0.104626	0.068271	0.220994	0.007082	0.00001	0.00001
Palestinians	HGDP00732	0.006321	0.618482	0.117508	0.054881	0.200137	0.00265	0.00001	0.00001
Palestinians	HGDP00737	0.005264	0.615345	0.113533	0.060289	0.199536	0.006013	0.00001	0.00001
Palestinians	HGDP00741	0.007908	0.55795	0.136019	0.070906	0.219826	0.002046	0.005336	0.00001
Palestinians	HGDP00746	0.006909	0.626319	0.092214	0.071406	0.201829	0.000778	0.000534	0.00001
Jordanians	Jordan305	0.012442	0.597552	0.096458	0.0605	0.228319	0.00001	0.00471	0.00001
Jordanians	Jordan384	0.009335	0.561906	0.114527	0.079197	0.227432	0.006782	0.00081	0.00001
Jordanians	Jordan444	0.024332	0.374369	0.014529	0.493471	0.088979	0.002276	0.002034	0.00001
Jordanians	Jordan485	0.005955	0.567537	0.104249	0.092257	0.220598	0.00001	0.009384	0.00001
Jordanians	Jordan503	0.023241	0.488516	0.119041	0.055935	0.283767	0.00001	0.00001	0.02948
Jordanians	Jordan560	0.014512	0.59711	0.101766	0.052201	0.229197	0.005193	0.00001	0.00001
Jordanians	Jordan603	0.00001	0.552841	0.141142	0.066072	0.224439	0.004853	0.000423	0.01022
Jordanians	Jordan62	0.006257	0.57639	0.101783	0.089013	0.222745	0.003791	0.00001	0.00001
Syrians	syria1	0.00001	0.416061	0.206322	0.016993	0.330355	0.001221	0.012362	0.016676
Syrians	syria2	0.014389	0.551	0.154058	0.041742	0.238781	0.00001	0.00001	0.00001
Syrians	syria361	0.00001	0.504172	0.14355	0.00001	0.352228	0.00001	0.00001	0.00001
Syrians	syria464	0.029953	0.549131	0.092196	0.060109	0.249108	0.003477	0.00001	0.016016
Syrians	syria520	0.00001	0.552033	0.159163	0.080703	0.201226	0.00001	0.006845	0.00001
Syrians	syria7	0.037282	0.55803	0.062351	0.062999	0.273866	0.002912	0.002551	0.00001
Syrians	syria9	0.010273	0.559128	0.154829	0.01646	0.252077	0.007212	0.00001	0.00001
Druze	HGDP00560	0.00001	0.578299	0.131929	0.010811	0.27369	0.005242	0.00001	0.00001
Druze	HGDP00564	0.00001	0.583934	0.120752	0.011502	0.283772	0.00001	0.00001	0.00001
Druze	HGDP00569	0.00001	0.561855	0.144765	0.023133	0.265641	0.004576	0.00001	0.00001
Druze	HGDP00573	0.009419	0.551722	0.168816	0.026049	0.243964	0.00001	0.00001	0.00001
Druze	HGDP00576	0.004623	0.553686	0.13858	0.05539	0.245359	0.002342	0.00001	0.00001
Druze	HGDP00579	0.00001	0.581255	0.15083	0.013766	0.245764	0.008355	0.00001	0.00001
Druze	HGDP00583	0.00001	0.581458	0.123566	0.01195	0.278722	0.000095	0.004188	0.00001
Druze	HGDP00586	0.002552	0.585233	0.143723	0.025545	0.242166	0.00001	0.00001	0.000761
Druze	HGDP00598	0.00442	0.578515	0.150014	0.023904	0.237331	0.005797	0.00001	0.00001
Druze	HGDP00601	0.00001	0.576886	0.142247	0.019288	0.259257	0.002291	0.00001	0.00001

Iraqis	Iraq01Irk	0.033325	0.454257	0.099503	0.02096	0.381727	0.00001	0.00054	0.009679
Iraqis	Iraq03Irk	0.017599	0.527279	0.098431	0.041614	0.312101	0.00001	0.00001	0.002955
Iraqis	Iraq04Irk	0.011195	0.489395	0.103931	0.048322	0.322106	0.003299	0.016014	0.005738
Iraqis	Iraq162jdn	0.030709	0.539554	0.089052	0.076558	0.244912	0.002094	0.003359	0.013762
Iraqis	Iraq198jdn	0.009863	0.465842	0.122737	0.05809	0.328555	0.006039	0.008865	0.00001
Iraqis	Iraq289Tr	0.010064	0.537844	0.059871	0.055822	0.292716	0.0136	0.007443	0.022642
Iraqis	Iraq307jdn	0.019982	0.505665	0.084059	0.054034	0.315828	0.00001	0.00284	0.017582
Turks	tur110	0.002768	0.404852	0.209129	0.001772	0.300638	0.002855	0.002474	0.075511
Turks	tur124	0.00001	0.348271	0.222333	0.003405	0.344729	0.00001	0.000624	0.080619
Turks	tur154	0.00001	0.41089	0.221053	0.00001	0.305792	0.00001	0.005209	0.057026
Turks	tur182	0.010121	0.402393	0.128627	0.006017	0.439099	0.00001	0.013724	0.00001
Turks	tur2	0.011738	0.369004	0.262552	0.00001	0.28187	0.005307	0.00001	0.069509
Turks	tur20	0.020039	0.387339	0.218351	0.00171	0.291615	0.0046	0.00001	0.076336
Turks	tur236	0.00001	0.383464	0.217425	0.001844	0.360016	0.003716	0.002962	0.030564
Turks	tur262	0.002215	0.413344	0.226779	0.00001	0.304334	0.010098	0.00001	0.04321
Turks	tur306	0.00001	0.243555	0.285376	0.00001	0.387819	0.00001	0.00001	0.08321
Kurds	kurd1159	0.015	0.410963	0.11427	0.001327	0.44642	0.00001	0.008311	0.003699
Kurds	kurd1156	0.011499	0.458218	0.093446	0.00001	0.42578	0.005632	0.005002	0.000415
Kurds	kurd1160	0.018571	0.38964	0.158272	0.001991	0.426472	0.001602	0.003442	0.00001
Kurds	kurd1101	0.017664	0.417922	0.129004	0.005238	0.426138	0.00001	0.004014	0.00001
Kurds	kurd1198	0.002451	0.409704	0.147737	0.00001	0.429489	0.006105	0.00001	0.004494
Kurds	kurd1173	0.03064	0.415351	0.120797	0.002237	0.422662	0.006825	0.00001	0.001478
Iranians	iran1	0.069667	0.454866	0.045416	0.129685	0.293077	0.000604	0.002906	0.003779
Iranians	iran11	0.038111	0.377045	0.121532	0.0079	0.42298	0.008513	0.022179	0.001739
Iranians	iran12	0.05069	0.420424	0.084188	0.005675	0.407043	0.002852	0.00001	0.029118
Iranians	iran13	0.032825	0.535391	0.066977	0.076506	0.283086	0.00001	0.00001	0.005193
Iranians	iran14	0.05442	0.35906	0.123303	0.008189	0.424781	0.00001	0.00001	0.030226
Iranians	iran15	0.025768	0.440799	0.074366	0.020908	0.425296	0.002913	0.00001	0.00994
Iranians	iran16	0.058047	0.3903	0.094405	0.003258	0.434671	0.002553	0.016754	0.00001
Iranians	iran17	0.060666	0.368225	0.096762	0.0245	0.434848	0.004485	0.010504	0.00001
Iranians	iran18	0.033533	0.355366	0.121066	0.009315	0.451034	0.016117	0.013559	0.00001
Iranians	iran19	0.025669	0.387951	0.133249	0.018488	0.408727	0.003555	0.00001	0.022351
Iranians	iran2	0.027951	0.368523	0.121736	0.006448	0.433931	0.006856	0.00884	0.025715
Iranians	iran20	0.058823	0.384737	0.107859	0.012664	0.405814	0.00001	0.006376	0.023718
Iranians	iran3	0.04285	0.406953	0.08168	0.019901	0.440408	0.00001	0.00001	0.008189
Iranians	iran4	0.036161	0.405267	0.104249	0.00014	0.438805	0.003865	0.00001	0.011502
Iranians	iran5	0.030662	0.442036	0.101882	0.019139	0.400455	0.003745	0.00001	0.002072
Iranians	iran6	0.047713	0.483637	0.086115	0.051115	0.322838	0.004366	0.002099	0.002116
Iranians	iran7	0.084813	0.349852	0.087785	0.140436	0.328403	0.002738	0.005962	0.00001
Iranians	iran8	0.040817	0.36712	0.131352	0.005111	0.435682	0.00001	0.00001	0.019898
Iranians	iran9	0.077952	0.41727	0.068795	0.04077	0.389214	0.000102	0.00001	0.005886
French	HGDP00512	0.00001	0.200995	0.661151	0.00001	0.137803	0.00001	0.00001	0.00001
French	HGDP00516	0.00001	0.203575	0.621006	0.00001	0.17537	0.00001	0.00001	0.00001
French	HGDP00518	0.000011	0.1864	0.685781	0.00001	0.126774	0.001005	0.00001	0.00001
French	HGDP00521	0.002173	0.222248	0.63193	0.00001	0.14174	0.001879	0.00001	0.00001
French	HGDP00523	0.00001	0.167117	0.704829	0.00001	0.128004	0.00001	0.00001	0.00001
French	HGDP00525	0.00001	0.186749	0.651202	0.000783	0.15524	0.00001	0.004615	0.001392
French	HGDP00527	0.00001	0.158711	0.683008	0.00001	0.153354	0.004887	0.00001	0.00001
French	HGDP00528	0.012681	0.16656	0.668596	0.000022	0.149276	0.002845	0.00001	0.00001
French	HGDP00529	0.00001	0.198817	0.651282	0.00001	0.149851	0.00001	0.00001	0.00001
French	HGDP00533	0.00001	0.095378	0.700934	0.00001	0.203638	0.00001	0.00001	0.00001
French	HGDP00535	0.00001	0.124334	0.710588	0.00001	0.165028	0.00001	0.00001	0.00001
French	HGDP00537	0.00001	0.200681	0.662482	0.00001	0.135634	0.001163	0.00001	0.00001
Sardinians	HGDP00665	0.00001	0.459051	0.540889	0.00001	0.00001	0.00001	0.00001	0.00001
Sardinians	HGDP00669	0.00001	0.424187	0.57575	0.000012	0.00001	0.00001	0.00001	0.00001
Sardinians	HGDP00672	0.00001	0.437599	0.562341	0.00001	0.00001	0.00001	0.00001	0.00001

Sardinians	HGDP01062	0.00001	0.450931	0.549006	0.00001	0.00001	0.000013	0.00001	0.00001
Sardinians	HGDP01064	0.00001	0.4297	0.57024	0.00001	0.00001	0.00001	0.00001	0.00001
Sardinians	HGDP01067	0.00001	0.444725	0.555215	0.00001	0.00001	0.00001	0.00001	0.00001
Sardinians	HGDP01068	0.00001	0.452183	0.547757	0.00001	0.00001	0.00001	0.00001	0.00001
Sardinians	HGDP01071	0.00001	0.441653	0.558287	0.00001	0.00001	0.00001	0.00001	0.00001
Sardinians	HGDP01073	0.00001	0.429866	0.570071	0.000014	0.00001	0.00001	0.00001	0.00001
Sardinians	HGDP01075	0.00001	0.43771	0.562228	0.000012	0.00001	0.00001	0.00001	0.00001
Sardinians	HGDP01077	0.00001	0.433513	0.566425	0.000012	0.00001	0.00001	0.00001	0.00001
Sardinians	HGDP01079	0.00001	0.413592	0.586348	0.00001	0.00001	0.00001	0.00001	0.00001
North_Italians	HGDP01147	0.00001	0.28088	0.595843	0.00001	0.123227	0.00001	0.00001	0.00001
North_Italians	HGDP01151	0.00001	0.28456	0.598059	0.00001	0.117331	0.00001	0.00001	0.00001
North_Italians	HGDP01153	0.00001	0.307817	0.562681	0.00001	0.129452	0.00001	0.00001	0.00001
North_Italians	HGDP01156	0.00001	0.316464	0.547393	0.00001	0.132277	0.003826	0.00001	0.00001
North_Italians	HGDP01157	0.00001	0.285215	0.586063	0.00001	0.126554	0.002127	0.00001	0.00001
North_Italians	HGDP01171	0.001231	0.305379	0.556901	0.00001	0.13645	0.00001	0.00001	0.00001
North_Italians	HGDP01173	0.00001	0.317438	0.571771	0.00001	0.110741	0.00001	0.00001	0.00001
North_Italians	HGDP01177	0.00001	0.31389	0.582174	0.00001	0.103886	0.00001	0.00001	0.00001
Tuscans	HGDP01162	0.00001	0.341386	0.492216	0.000309	0.166048	0.00001	0.00001	0.00001
Tuscans	HGDP01163	0.00001	0.322521	0.502543	0.00001	0.174886	0.00001	0.00001	0.00001
Tuscans	HGDP01164	0.00001	0.346953	0.519542	0.00001	0.133455	0.00001	0.00001	0.00001
Tuscans	HGDP01166	0.00001	0.339099	0.515954	0.00001	0.144897	0.00001	0.00001	0.00001
Tuscans	HGDP01167	0.000011	0.362727	0.460014	0.00001	0.177091	0.00001	0.00001	0.000127
Tuscans	HGDP01168	0.00001	0.351536	0.515619	0.00001	0.132795	0.00001	0.00001	0.00001
Tuscans	HGDP01169	0.00001	0.363302	0.479256	0.00001	0.157391	0.00001	0.00001	0.00001
Russians	RussianVoron1(	0.00001	0.068842	0.751399	0.00001	0.160941	0.00001	0.00001	0.018778
Russians	russianVoron1(	0.004236	0.011088	0.777991	0.000018	0.17992	0.00001	0.00001	0.026727
Russians	RussianKursk15	0.005164	0.026399	0.749358	0.000011	0.203779	0.004149	0.00001	0.011129
Russians	RussianKursk18	0.00001	0.005394	0.761731	0.00001	0.213994	0.00001	0.00001	0.01884
Russians	RussianKursk9	0.004133	0.027822	0.757145	0.00001	0.182954	0.003282	0.009336	0.015318
Russians	russianKursk6	0.009281	0.008332	0.773276	0.00001	0.193611	0.00001	0.00001	0.015471
Russians	RussianOrjol34	0.00001	0.023484	0.754904	0.00001	0.194634	0.00001	0.00001	0.026937
Russians	russianOrjol1	0.015642	0.018703	0.755794	0.00001	0.168196	0.007844	0.00001	0.033801
Russians	russianOrjol56	0.00001	0.02602	0.730873	0.00001	0.237433	0.005634	0.00001	0.00001
Russians	russianOrjol78	0.00001	0.025725	0.775451	0.00001	0.181851	0.00001	0.00001	0.016933
Russians	russianOrjol89	0.000229	0.025172	0.758285	0.00001	0.201867	0.00001	0.00001	0.014417
Estonians	a39	0.00001	0.00001	0.810237	0.00001	0.158582	0.00001	0.00001	0.03113
Estonians	ee1	0.011958	0.00001	0.79671	0.00001	0.150416	0.000811	0.00001	0.040075
Estonians	ee105	0.00001	0.00001	0.815966	0.00001	0.172536	0.007021	0.001848	0.002598
Estonians	ee108	0.004497	0.00001	0.805618	0.00001	0.171394	0.00001	0.00001	0.01845
Estonians	ee111	0.000638	0.00001	0.818002	0.00001	0.157015	0.007351	0.00001	0.016965
Estonians	ee114	0.00001	0.00001	0.814429	0.00001	0.162016	0.00001	0.00001	0.023506
Estonians	ee136	0.00001	0.00001	0.813808	0.00001	0.150061	0.00001	0.00001	0.036081
Estonians	ee140	0.00001	0.00001	0.795915	0.00001	0.179254	0.000403	0.00001	0.024388
Estonians	ee147	0.013516	0.00001	0.843369	0.000012	0.136154	0.00001	0.00001	0.006918
Estonians	ee149	0.01023	0.00001	0.801737	0.00001	0.153351	0.007193	0.00001	0.027459
Estonians	ee50	0.005047	0.00001	0.805393	0.00001	0.158817	0.00001	0.00001	0.030703
Armenians	arm10	0.000833	0.491142	0.128105	0.00001	0.37988	0.00001	0.00001	0.00001
Armenians	arm11	0.00001	0.478089	0.150978	0.00001	0.370883	0.00001	0.00001	0.00001
Armenians	arm12	0.00001	0.478607	0.150446	0.00001	0.370897	0.00001	0.00001	0.00001
Armenians	arm13	0.00001	0.384712	0.256218	0.00001	0.35902	0.00001	0.00001	0.00001
Armenians	arm14	0.00001	0.25313	0.452817	0.00001	0.280119	0.009119	0.00001	0.004785
Armenians	arm17	0.00001	0.489038	0.155855	0.00001	0.353702	0.001365	0.00001	0.00001
Armenians	arm18	0.00001	0.493617	0.166701	0.00001	0.339632	0.00001	0.00001	0.00001
Armenians	arm19	0.00001	0.492429	0.13349	0.00001	0.374031	0.00001	0.00001	0.00001
Armenians	arm20	0.00001	0.476793	0.135749	0.00001	0.387408	0.00001	0.00001	0.00001
Armenians	arm21	0.00001	0.122409	0.625652	0.00001	0.247954	0.003944	0.00001	0.00001

Armenians	arm23	0.00001	0.496375	0.152304	0.00001	0.351271	0.00001	0.00001	0.00001
Armenians	arm26	0.00001	0.481651	0.134147	0.00001	0.384152	0.00001	0.00001	0.00001
Armenians	arm3	0.00001	0.499877	0.131545	0.00001	0.365502	0.002758	0.000288	0.00001
Armenians	arm4	0.00001	0.452611	0.142415	0.00001	0.404923	0.00001	0.00001	0.00001
Armenians	arm5	0.00001	0.486408	0.144041	0.00001	0.369501	0.00001	0.00001	0.00001
Armenians	arm6	0.00001	0.490447	0.138132	0.00001	0.371371	0.00001	0.00001	0.00001
Armenians	arm7	0.00001	0.247457	0.431207	0.00001	0.277546	0.00845	0.00001	0.03531
Armenians	arm8	0.00001	0.496772	0.150096	0.000013	0.353079	0.00001	0.00001	0.00001
Armenians	arm9	0.00001	0.456935	0.168523	0.00001	0.372851	0.00001	0.00001	0.001651
Georgians	mg20	0.00001	0.43968	0.142363	0.00001	0.417907	0.00001	0.00001	0.00001
Georgians	mg22	0.00001	0.42497	0.159977	0.00001	0.415003	0.00001	0.00001	0.00001
Georgians	mg23	0.00001	0.435306	0.146605	0.00001	0.417805	0.000243	0.00001	0.00001
Georgians	mg27	0.00001	0.428967	0.143003	0.00001	0.42798	0.00001	0.00001	0.00001
Georgians	mg31	0.00001	0.417145	0.173678	0.00001	0.409127	0.00001	0.00001	0.00001
Georgians	mg34	0.000014	0.430589	0.158314	0.000011	0.410612	0.00044	0.00001	0.00001
Georgians	mg40	0.00001	0.422239	0.13653	0.00001	0.441181	0.00001	0.00001	0.00001
Georgians	mg43	0.00001	0.384822	0.167536	0.00001	0.447592	0.00001	0.00001	0.00001
Georgians	mg47	0.00001	0.438862	0.158206	0.00001	0.402881	0.00001	0.00001	0.00001
Georgians	mg49	0.000012	0.441513	0.132479	0.00001	0.425956	0.00001	0.00001	0.00001
Georgians	mg5	0.00001	0.432878	0.154047	0.00001	0.413025	0.00001	0.00001	0.00001
Georgians	mg51	0.00001	0.360441	0.237711	0.00001	0.380054	0.000969	0.00001	0.020795
Georgians	mg54	0.00001	0.411465	0.150871	0.00001	0.437614	0.00001	0.00001	0.00001
Georgians	mg61	0.00001	0.320543	0.321684	0.00001	0.357722	0.00001	0.00001	0.00001
Georgians	mg62	0.00001	0.425532	0.13499	0.00001	0.439427	0.00001	0.00001	0.00001
Azeris	azerF4	0.029119	0.385714	0.157671	0.004092	0.374141	0.000556	0.002738	0.045967
Azeris	azerE3	0.019989	0.408867	0.144459	0.002363	0.383604	0.00001	0.007439	0.033269
Azeris	azerB64	0.016128	0.379839	0.140193	0.00338	0.396957	0.00001	0.016126	0.047368
Azeris	azerF111	0.014736	0.38198	0.153313	0.000662	0.37901	0.00001	0.00001	0.070278
Azeris	azerE70	0.013616	0.395392	0.115989	0.007809	0.407657	0.00001	0.017767	0.04176
Azeris	azerE6	0.010042	0.39288	0.17173	0.002379	0.354151	0.00001	0.002746	0.066061
Azeris	azerE85	0.023117	0.395942	0.154565	0.000263	0.366801	0.005621	0.013108	0.040582
Azeris	azerE89	0.019474	0.387869	0.147409	0.00001	0.377535	0.011289	0.02332	0.033094
Azeris	azerB38	0.017439	0.395856	0.13553	0.008087	0.387889	0.00001	0.009789	0.045399
Azeris	azerE1	0.018697	0.376426	0.15906	0.00553	0.399307	0.00001	0.00001	0.040961
Azeris	azerE92	0.002488	0.397771	0.124477	0.006126	0.400873	0.013416	0.009548	0.045302
Azeris	azerb72	0.029324	0.378874	0.152435	0.005926	0.381392	0.00001	0.014093	0.037946
Azeris	azerB59	0.02156	0.393246	0.137469	0.002898	0.363284	0.001907	0.019265	0.060371
Azeris	azerB61	0.021598	0.405753	0.151198	0.000635	0.372315	0.006262	0.007567	0.034672
Azeris	azerE82	0.016853	0.385303	0.152546	0.004315	0.371509	0.003859	0.017811	0.047804
Azeris	azerB8	0.016685	0.411068	0.143537	0.0005	0.372955	0.00073	0.018362	0.036161
Abkhasians	abh107	0.00001	0.402157	0.13466	0.00001	0.459425	0.00001	0.003717	0.00001
Abkhasians	abh122	0.00001	0.396408	0.186968	0.00001	0.409594	0.00001	0.005024	0.001976
Abkhasians	abh135	0.00001	0.392994	0.188091	0.00001	0.415779	0.00001	0.000499	0.002607
Abkhasians	abh154	0.00001	0.454051	0.125731	0.000012	0.420167	0.00001	0.00001	0.00001
Abkhasians	abh41	0.00001	0.359311	0.179687	0.00001	0.456592	0.00001	0.00001	0.00437
Abkhasians	abh53	0.00001	0.3844	0.181433	0.00001	0.41182	0.00001	0.00001	0.022307
Abkhasians	abh71	0.00001	0.384396	0.173611	0.00001	0.433587	0.000755	0.00001	0.007621
Balkars	bal102	0.000012	0.279018	0.26849	0.00001	0.387292	0.000111	0.009562	0.055505
Balkars	bal115	0.00001	0.314182	0.24522	0.00001	0.382575	0.00001	0.00001	0.057982
Balkars	bal14	0.00001	0.289466	0.248376	0.00001	0.383582	0.004801	0.003073	0.070682
Balkars	bal149	0.00001	0.184816	0.474285	0.00001	0.290024	0.000239	0.00001	0.050607
Balkars	bal26	0.00001	0.303062	0.250606	0.00001	0.374487	0.00001	0.00001	0.071805
Balkars	bal32	0.00001	0.316517	0.238054	0.00001	0.394644	0.00001	0.014327	0.036428
Balkars	bal45	0.00001	0.299276	0.247114	0.00001	0.404847	0.00001	0.012602	0.03613
Balkars	bal7	0.00001	0.284782	0.256205	0.00001	0.379828	0.004598	0.00001	0.074557
Chechens	ch101	0.00001	0.199443	0.421031	0.00001	0.358748	0.000759	0.014673	0.005325

Chechens	ch126	0.00001	0.252096	0.264725	0.00001	0.456051	0.00001	0.010158	0.01694
Chechens	ch150	0.00001	0.279146	0.252702	0.00001	0.451539	0.00001	0.00001	0.016573
Chechens	ch174	0.00001	0.251504	0.287135	0.00001	0.43132	0.004511	0.00001	0.0255
Chechens	ch193	0.00001	0.287977	0.251318	0.00001	0.433495	0.00001	0.00001	0.02717
Chechens	ch3	0.00001	0.272182	0.28904	0.00001	0.415386	0.00001	0.00001	0.023353
Chechens	ch34	0.00001	0.25929	0.257353	0.00001	0.457058	0.009491	0.006599	0.01019
Chechens	ch60	0.00001	0.219114	0.367397	0.00001	0.394353	0.00001	0.001038	0.018068
Chechens	ch86	0.00001	0.245871	0.279462	0.000079	0.43868	0.010445	0.00001	0.025442
Lezgins	lez13	0.00001	0.233341	0.279649	0.00001	0.471298	0.00001	0.00001	0.015673
Lezgins	lez17	0.00001	0.267194	0.264437	0.00001	0.462901	0.00001	0.00001	0.005428
Lezgins	lez3	0.00001	0.195201	0.28225	0.00001	0.502412	0.00001	0.00001	0.020097
Lezgins	lez33	0.001287	0.241957	0.275928	0.000412	0.468924	0.00001	0.00001	0.011473
Lezgins	lez37	0.00001	0.247294	0.256802	0.00001	0.480814	0.004999	0.008327	0.001745
Lezgins	lez42	0.00001	0.243829	0.259296	0.00001	0.492719	0.00001	0.004117	0.00001
Lezgins	lez49	0.00001	0.24612	0.269776	0.00001	0.471026	0.00001	0.00175	0.011298
Lezgins	lez7	0.00001	0.274413	0.230058	0.00001	0.472698	0.00017	0.00001	0.022632
Kumyks	kumyks1	0.00001	0.24332	0.271932	0.00001	0.373075	0.00001	0.00001	0.111633
Kumyks	kumyks108	0.00001	0.294421	0.201841	0.00001	0.441778	0.006197	0.023199	0.032543
Kumyks	kumyks111	0.00001	0.314712	0.236459	0.00001	0.399838	0.001508	0.00001	0.047453
Kumyks	kumyks4	0.00001	0.270879	0.284631	0.00001	0.381668	0.00001	0.008524	0.054267
Kumyks	kumyks6	0.005967	0.258471	0.27232	0.00001	0.389453	0.00001	0.00001	0.073759
Kumyks	kumyks8	0.00001	0.279182	0.240807	0.00001	0.409097	0.001618	0.00001	0.069267
Kumyks	Kumyk22	0.00001	0.314616	0.215031	0.00001	0.411313	0.00001	0.00001	0.059
Nogais	nogay10	0.00001	0.175562	0.26519	0.00001	0.278518	0.004058	0.02098	0.255672
Nogais	nogay12	0.00001	0.225169	0.275994	0.00001	0.312035	0.001083	0.000362	0.185337
Nogais	nogay13	0.00001	0.192423	0.251974	0.00001	0.299823	0.00001	0.004164	0.251587
Nogais	nogay15	0.000883	0.148237	0.275148	0.00001	0.233143	0.007295	0.029044	0.306239
Nogais	nogay2	0.00001	0.220389	0.247403	0.00001	0.309087	0.00001	0.001576	0.221515
Nogais	nogay3	0.00001	0.179263	0.311698	0.00001	0.306255	0.011764	0.00001	0.190991
Nogais	nogay4	0.00001	0.192952	0.268309	0.00001	0.301971	0.005883	0.011249	0.219617
Nogais	nogay6	0.00001	0.184134	0.38188	0.00001	0.28909	0.00001	0.012859	0.132008
Nogais	nogay7	0.00001	0.178951	0.353974	0.00001	0.309102	0.00001	0.00001	0.157934
Nogais	nogay8	0.00001	0.191094	0.297525	0.00001	0.327806	0.00001	0.00001	0.183535
Turkmens	turkm1820	0.076952	0.265497	0.155498	0.00456	0.386773	0.00001	0.000065	0.110645
Turkmens	turkm7529	0.060519	0.18915	0.19099	0.008237	0.349791	0.009268	0.00001	0.192036
Turkmens	turkmE31	0.076374	0.285813	0.135376	0.00001	0.383457	0.002427	0.004952	0.11159
Turkmens	turkmen2	0.077018	0.166353	0.194293	0.003953	0.303292	0.003109	0.035494	0.216489
Turkmens	turkmu26	0.049445	0.267994	0.162257	0.00001	0.380034	0.006871	0.026502	0.106887
Tajiks	tad838	0.09587	0.161533	0.213877	0.00001	0.434504	0.00001	0.00001	0.094186
Tajiks	tadjik10	0.088613	0.139649	0.237239	0.00001	0.420522	0.006094	0.000568	0.107305
Tajiks	tadjik12	0.090316	0.159313	0.143906	0.001896	0.401495	0.00001	0.003488	0.199577
Tajiks	tadjik14	0.102157	0.154003	0.207146	0.002235	0.431035	0.002111	0.013383	0.08793
Tajiks	tadjik2	0.072103	0.133507	0.224113	0.005561	0.439506	0.00001	0.003146	0.122054
Tajiks	tadjik3	0.087083	0.143693	0.214295	0.00001	0.443144	0.00001	0.008221	0.103545
Tajiks	tadjik5	0.08597	0.142363	0.216106	0.002157	0.390779	0.005072	0.007097	0.150457
Tajiks	tadjik6	0.079128	0.133467	0.198244	0.002945	0.399079	0.009398	0.048417	0.129323
Tajiks	tadjik8	0.066298	0.125506	0.21576	0.005892	0.440366	0.004261	0.021412	0.120506
Tajiks	tadjik9	0.09834	0.140559	0.213458	0.001135	0.413687	0.00001	0.017257	0.115554
Uzbeks	usb1	0.042937	0.103479	0.197817	0.00001	0.227306	0.001371	0.022706	0.404374
Uzbeks	usb2	0.053431	0.094558	0.193374	0.002456	0.207367	0.00001	0.00001	0.448794
Uzbeks	usb25	0.04894	0.100794	0.17535	0.000117	0.21661	0.001847	0.028158	0.428183
Uzbeks	usb35	0.044797	0.099933	0.201679	0.004883	0.245277	0.00001	0.046586	0.356834
Uzbeks	usb64	0.085838	0.113876	0.195437	0.00001	0.349131	0.005694	0.040319	0.209694
Uzbeks	usb78	0.052391	0.133059	0.224607	0.008604	0.294775	0.002439	0.038904	0.245221
Uzbeks	usb83	0.038296	0.146855	0.231242	0.00001	0.346866	0.003116	0.027635	0.205979
Uzbeks	uzbek80	0.05284	0.129029	0.20038	0.006373	0.287601	0.000023	0.022272	0.301481

Uzbeks	uzbek55	0.085114	0.178274	0.157766	0.001389	0.340816	0.00001	0.026024	0.210608
Kyrgyzians	kirgiz21	0.032161	0.040767	0.12902	0.00001	0.136283	0.003266	0.03331	0.625183
Kyrgyzians	kirgiz24	0.020478	0.029519	0.148649	0.00001	0.152147	0.00001	0.017797	0.631391
Kyrgyzians	kirgiz28	0.025529	0.00001	0.159875	0.00001	0.154844	0.006466	0.066409	0.586858
Kyrgyzians	kirgiz31	0.018336	0.047734	0.150662	0.006542	0.168127	0.00628	0.052201	0.550118
Kyrgyzians	kirgiz35	0.025004	0.026239	0.139708	0.000182	0.177011	0.008617	0.031075	0.592166
Hazara	HGDP00100	0.072108	0.098632	0.143898	0.00001	0.284147	0.00001	0.033044	0.368151
Hazara	HGDP00104	0.098929	0.088863	0.134729	0.006835	0.32275	0.00001	0.01356	0.334324
Hazara	HGDP00109	0.062482	0.078427	0.099021	0.000228	0.264239	0.008269	0.046407	0.440928
Hazara	HGDP00112	0.06309	0.072874	0.100701	0.001745	0.218437	0.00001	0.050152	0.492991
Hazara	HGDP00118	0.082103	0.067061	0.12178	0.005031	0.262495	0.004991	0.027449	0.429089
Hazara	HGDP00121	0.078971	0.060799	0.116713	0.00001	0.245136	0.005289	0.076164	0.416918
Hazara	HGDP00124	0.085872	0.046351	0.148517	0.000498	0.255571	0.00534	0.060031	0.397821
Hazara	HGDP00127	0.067953	0.070655	0.129015	0.003582	0.222161	0.001258	0.045473	0.459902
Hazara	HGDP00129	0.075305	0.189186	0.130864	0.00001	0.333188	0.009208	0.052148	0.21009
Kalash	HGDP00267	0.06889	0.00001	0.00001	0.00001	0.897789	0.008468	0.024812	0.00001
Kalash	HGDP00277	0.09457	0.00001	0.00001	0.000437	0.860544	0.007616	0.036804	0.00001
Kalash	HGDP00281	0.086	0.00001	0.00001	0.00001	0.863716	0.01184	0.038404	0.00001
Kalash	HGDP00288	0.085641	0.00001	0.00001	0.00001	0.862782	0.022243	0.029295	0.00001
Kalash	HGDP00298	0.103896	0.00001	0.017736	0.00001	0.826578	0.016559	0.035202	0.00001
Kalash	HGDP00309	0.083874	0.00001	0.00001	0.00001	0.858479	0.018201	0.039406	0.00001
Kalash	HGDP00313	0.109452	0.00001	0.00001	0.00001	0.850882	0.011678	0.027949	0.00001
Kalash	HGDP00323	0.06571	0.00001	0.00001	0.00001	0.884397	0.016756	0.033097	0.00001
Kalash	HGDP00328	0.06788	0.00001	0.00001	0.00001	0.883429	0.010662	0.037989	0.00001
Pathan	HGDP00216	0.28599	0.106471	0.104726	0.00001	0.475103	0.003367	0.003333	0.020999
Pathan	HGDP00222	0.294024	0.087242	0.108817	0.00001	0.487401	0.006203	0.007323	0.00898
Pathan	HGDP00228	0.292468	0.089412	0.128539	0.00441	0.461462	0.000442	0.00001	0.023257
Pathan	HGDP00232	0.333113	0.088623	0.070122	0.00001	0.505066	0.00001	0.00001	0.003045
Pathan	HGDP00237	0.380139	0.073337	0.092009	0.006294	0.420439	0.006604	0.00001	0.021167
Pathan	HGDP00241	0.264012	0.117721	0.12434	0.00001	0.48015	0.003584	0.00001	0.010173
Pathan	HGDP00244	0.239116	0.104166	0.104841	0.00931	0.500136	0.000934	0.00001	0.041487
Pathan	HGDP00254	0.243792	0.118262	0.12186	0.001783	0.495055	0.00001	0.00164	0.017598
Pathan	HGDP00258	0.319016	0.093905	0.081653	0.002426	0.468941	0.003206	0.00001	0.030843
Pathan	HGDP00262	0.243053	0.130664	0.120205	0.00001	0.474451	0.00001	0.00001	0.031596
Pathan	HGDP00264	0.29447	0.101946	0.105436	0.003467	0.464842	0.001347	0.002461	0.02603
Burusho	HGDP00338	0.233579	0.065544	0.10486	0.005007	0.464852	0.011525	0.023932	0.090701
Burusho	HGDP00346	0.2627	0.049944	0.091861	0.00001	0.467513	0.007849	0.035631	0.084493
Burusho	HGDP00356	0.250085	0.048582	0.103344	0.00001	0.479431	0.009451	0.018613	0.090485
Burusho	HGDP00364	0.255943	0.065683	0.094597	0.000071	0.471104	0.010049	0.024985	0.077568
Burusho	HGDP00372	0.25316	0.04462	0.098908	0.00001	0.471312	0.002979	0.02249	0.10652
Burusho	HGDP00388	0.284036	0.061039	0.084552	0.00368	0.469179	0.00065	0.030635	0.066228
Burusho	HGDP00397	0.263801	0.028684	0.113571	0.002561	0.472969	0.001908	0.016155	0.10035
Burusho	HGDP00412	0.28664	0.057963	0.108599	0.00001	0.435334	0.013387	0.002821	0.095247
Burusho	HGDP00423	0.249394	0.064533	0.088045	0.006511	0.472264	0.002831	0.041543	0.074879
Burusho	HGDP00433	0.270834	0.04531	0.108684	0.001862	0.477084	0.002526	0.00001	0.09369
Burusho	HGDP00444	0.271688	0.041382	0.097921	0.00001	0.483096	0.00001	0.021689	0.084204
Balochi	HGDP00054	0.162002	0.231011	0.03134	0.000798	0.572958	0.001872	0.00001	0.00001
Balochi	HGDP00057	0.151932	0.224235	0.035994	0.15701	0.427184	0.00001	0.001087	0.002548
Balochi	HGDP00060	0.164575	0.239543	0.00001	0.118116	0.471733	0.006003	0.00001	0.00001
Balochi	HGDP00066	0.173659	0.221985	0.01652	0.009182	0.571623	0.006595	0.000426	0.00001
Balochi	HGDP00070	0.464602	0.040942	0.05547	0.00001	0.394296	0.002718	0.024637	0.017324
Balochi	HGDP00076	0.149434	0.231635	0.00001	0.097243	0.511987	0.002538	0.00001	0.007142
Balochi	HGDP00080	0.178892	0.241368	0.00001	0.01578	0.563919	0.00001	0.00001	0.00001
Balochi	HGDP00086	0.163111	0.215805	0.003791	0.008703	0.60856	0.00001	0.00001	0.00001
Balochi	HGDP00090	0.247245	0.151936	0.023809	0.021562	0.555418	0.00001	0.00001	0.00001
Balochi	HGDP00094	0.233172	0.190397	0.035382	0.013203	0.524532	0.002472	0.00001	0.000831

Balochi	HGDP00098	0.212499	0.166634	0.034339	0.039136	0.541742	0.00001	0.00001	0.00563
Brahui	HGDP00005	0.180465	0.209467	0.001098	0.010757	0.578351	0.005578	0.00001	0.014274
Brahui	HGDP00007	0.172019	0.202566	0.009982	0.011003	0.597156	0.00001	0.007254	0.00001
Brahui	HGDP00011	0.174312	0.216335	0.00001	0.007021	0.593004	0.00001	0.009299	0.00001
Brahui	HGDP00015	0.17478	0.230935	0.00001	0.007719	0.586109	0.000427	0.00001	0.00001
Brahui	HGDP00019	0.161743	0.205368	0.047553	0.012258	0.533077	0.005116	0.00001	0.034876
Brahui	HGDP00023	0.164388	0.243844	0.00001	0.010176	0.580673	0.000888	0.00001	0.00001
Brahui	HGDP00027	0.201471	0.193655	0.007512	0.026907	0.558765	0.007353	0.004326	0.00001
Brahui	HGDP00033	0.163594	0.226972	0.015656	0.008877	0.579109	0.005773	0.00001	0.00001
Brahui	HGDP00039	0.25249	0.172778	0.017948	0.030273	0.506854	0.012531	0.007116	0.00001
Brahui	HGDP00045	0.169046	0.214359	0.00001	0.005773	0.603295	0.001993	0.00001	0.005513
Makrani	HGDP00130	0.123227	0.155551	0.007067	0.374256	0.31557	0.002902	0.021416	0.00001
Makrani	HGDP00135	0.163848	0.234703	0.01484	0.008944	0.571384	0.006262	0.00001	0.00001
Makrani	HGDP00139	0.140972	0.218259	0.014187	0.128206	0.497631	0.000726	0.00001	0.00001
Makrani	HGDP00141	0.147009	0.250688	0.011086	0.049885	0.539845	0.001467	0.00001	0.00001
Makrani	HGDP00145	0.162791	0.237988	0.025976	0.050375	0.521603	0.00001	0.001247	0.00001
Makrani	HGDP00149	0.287046	0.136816	0.032361	0.070564	0.459782	0.00001	0.013411	0.00001
Makrani	HGDP00151	0.152337	0.235717	0.00001	0.053521	0.545949	0.010083	0.002373	0.00001
Makrani	HGDP00153	0.162849	0.266833	0.00001	0.073121	0.497157	0.00001	0.00001	0.00001
Makrani	HGDP00158	0.14076	0.223615	0.00001	0.012254	0.617569	0.004473	0.001309	0.00001
Makrani	HGDP00161	0.164463	0.229323	0.00001	0.044937	0.561237	0.00001	0.00001	0.00001
Sindhi	HGDP00165	0.3404	0.110224	0.023867	0.033322	0.475995	0.00144	0.00001	0.014742
Sindhi	HGDP00169	0.39249	0.088189	0.044209	0.004374	0.468961	0.00001	0.00001	0.001758
Sindhi	HGDP00177	0.328294	0.097679	0.027473	0.079173	0.455794	0.00361	0.00001	0.007968
Sindhi	HGDP00179	0.333025	0.120648	0.01265	0.002625	0.527849	0.003184	0.00001	0.00001
Sindhi	HGDP00185	0.297749	0.103262	0.089683	0.005955	0.490233	0.003707	0.00001	0.009402
Sindhi	HGDP00191	0.321616	0.086661	0.099655	0.00001	0.47976	0.00001	0.00336	0.008928
Sindhi	HGDP00195	0.389673	0.094921	0.031019	0.002614	0.471749	0.006875	0.00001	0.003139
Sindhi	HGDP00199	0.216414	0.19292	0.003207	0.009785	0.573394	0.004261	0.00001	0.00001
Sindhi	HGDP00205	0.317168	0.11881	0.059731	0.00001	0.495055	0.00001	0.00001	0.009206
Sindhi	HGDP00210	0.340943	0.103993	0.050345	0.005308	0.48442	0.000016	0.00001	0.014967
Parsi_Pakistan	EGYPTLC55443	0.149364	0.352628	0.00001	0.008457	0.458886	0.01451	0.004434	0.004536
Parsi_Pakistan	EGYPTLC55443	0.183324	0.330774	0.00001	0.00001	0.471761	0.011794	0.002308	0.00001
Parsi_Pakistan	EGYPTLC55443	0.117964	0.393942	0.00001	0.000524	0.466913	0.012464	0.006449	0.00001
Parsi_Pakistan	EGYPTLC55443	0.131912	0.370549	0.00001	0.00001	0.481524	0.007624	0.006776	0.00001
Parsi_Pakistan	EGYPTLC55443	0.138649	0.388048	0.00001	0.002291	0.463252	0.00001	0.00772	0.00001
Parsi_Pakistan	EGYPTLC55443	0.156548	0.362942	0.00001	0.00366	0.464499	0.009542	0.00278	0.00001
Parsi_Pakistan	EGYPTLC55443	0.146749	0.350255	0.00001	0.010432	0.48089	0.00001	0.004647	0.004326
Parsi_Pakistan	EGYPTLC55443	0.127407	0.394476	0.00001	0.007543	0.457253	0.005407	0.00001	0.006864
Parsi_Pakistan	EGYPTLC55444	0.120514	0.3896	0.00001	0.00001	0.474217	0.009938	0.00569	0.00001
Parsi_Pakistan	EGYPTLC55443	0.100209	0.414547	0.016874	0.000783	0.462554	0.001056	0.00001	0.003958
Parsi_Pakistan	EGYPTLC55443	0.088719	0.374816	0.021775	0.006277	0.497141	0.00001	0.010469	0.00001
Parsi_Pakistan	EGYPTLC55444	0.124851	0.402279	0.00001	0.00001	0.460462	0.00715	0.000018	0.00001
Parsi_Pakistan	EGYPTLC55443	0.128278	0.366305	0.00001	0.00581	0.491603	0.003789	0.00001	0.00001
Parsi_Pakistan	EGYPTLC55443	0.102216	0.387034	0.00001	0.00001	0.49579	0.012779	0.002142	0.00001
Parsi_Pakistan	EGYPTLC55444	0.13776	0.349756	0.00001	0.008202	0.484463	0.006146	0.013136	0.00001
Parsi_Pakistan	EGYPTLC55443	0.126114	0.378019	0.00001	0.006111	0.476866	0.000165	0.012695	0.00001
Parsi_Pakistan	EGYPTLC55443	0.145583	0.384534	0.00001	0.00001	0.458538	0.011047	0.000258	0.00001
Parsi_Pakistan	EGYPTLC55444	0.116487	0.388814	0.00001	0.00001	0.480652	0.0135	0.00001	0.00001
Parsi_Pakistan	EGYPTLC55443	0.111272	0.421084	0.00001	0.001061	0.461715	0.004828	0.00001	0.00001
Parsi_Pakistan	EGYPTLC55443	0.039169	0.361555	0.122811	0.004276	0.464718	0.00001	0.003989	0.003462
Parsi_Pakistan	EGYPTLC55444	0.112853	0.358543	0.00001	0.003924	0.504465	0.00001	0.015006	0.00001
Parsi_Pakistan	EGYPTLC55443	0.149479	0.365494	0.00001	0.00001	0.475618	0.009359	0.00001	0.00001
Parsi_Pakistan	EGYPTLC55443	0.226411	0.321365	0.00001	0.005571	0.440578	0.005341	0.00001	0.00001
Parsi_Pakistan	EGYPTLC55444	0.162141	0.361621	0.00001	0.005599	0.46488	0.00001	0.005718	0.00001
Parsis_India	ParsiZ177	0.141042	0.358572	0.00001	0.000634	0.487918	0.00001	0.010456	0.001357

Parsis_India	ParsiZ178	0.163396	0.37516	0.00001	0.0024	0.449516	0.002087	0.00001	0.00742
Parsis_India	ParsiZ179	0.138015	0.363841	0.00001	0.000598	0.479344	0.006405	0.011777	0.00001
Parsis_India	ParsiZ180	0.146034	0.371266	0.00001	0.000809	0.462879	0.005248	0.013744	0.00001
Parsis_India	ParsiZ181	0.139822	0.352102	0.00001	0.001042	0.497646	0.009358	0.00001	0.00001
Parsis_India	ParsiZ182	0.150798	0.353217	0.00001	0.00001	0.482468	0.008079	0.005408	0.00001
Parsis_India	ParsiZ183	0.132423	0.375796	0.00001	0.00001	0.487206	0.001081	0.003464	0.00001
Parsis_India	ParsiZ185	0.126391	0.362882	0.00001	0.00001	0.491908	0.006333	0.012456	0.00001
Parsis_India	ParsiZ186	0.165333	0.324081	0.00001	0.00001	0.497269	0.006498	0.006789	0.00001
Parsis_India	Parsi26	0.097444	0.363815	0.00001	0.00001	0.51064	0.013326	0.013266	0.001489
Parsis_India	Parsi10	0.151065	0.344287	0.00001	0.00001	0.491132	0.007233	0.006121	0.000143
Parsis_India	Parsi7	0.13417	0.345011	0.00433	0.00001	0.495416	0.012739	0.008185	0.000138
Parsis_India	Parsi8	0.174891	0.336101	0.00001	0.00001	0.476079	0.009848	0.003051	0.00001
Parsis_India	Parsi34	0.13567	0.33896	0.00001	0.002155	0.502801	0.007588	0.012807	0.00001
Parsis_India	Parsi2	0.161537	0.348668	0.00001	0.003693	0.479452	0.002376	0.004255	0.00001
Parsis_India	Parsi4	0.138278	0.347374	0.021214	0.00001	0.466743	0.00301	0.018302	0.00507
Parsis_India	Parsi11	0.142762	0.348574	0.00001	0.00001	0.493802	0.002742	0.012091	0.00001
Parsis_India	Parsi13	0.149519	0.345891	0.00001	0.0012	0.49198	0.01138	0.00001	0.00001
Parsis_India	Parsi24	0.249493	0.286577	0.00001	0.00001	0.440814	0.007965	0.013112	0.002019
Gujaratis	NA20849	0.592049	0.02937	0.000013	0.00001	0.378529	0.00001	0.00001	0.00001
Gujaratis	NA20866	0.503879	0.024929	0.061766	0.003806	0.397356	0.00001	0.008245	0.00001
Gujaratis	NA21086	0.579946	0.010775	0.025708	0.00001	0.367372	0.005366	0.008528	0.002294
Gujaratis	NA21125	0.588963	0.00585	0.017529	0.00001	0.3826	0.002843	0.00001	0.002195
Gujaratis	NA21142	0.582996	0.011688	0.006549	0.00001	0.395398	0.000458	0.00001	0.002891
Gujaratis	NA21098	0.573771	0.022751	0.00001	0.001421	0.393351	0.00001	0.008676	0.00001
Gujaratis	NA21109	0.585285	0.034184	0.000817	0.00001	0.379674	0.00001	0.00001	0.00001
Gujaratis	NA20900	0.445422	0.024907	0.111335	0.00001	0.400956	0.00001	0.00001	0.017349
Gujaratis	NA21102	0.586962	0.020484	0.00001	0.00001	0.392504	0.00001	0.00001	0.00001
Gujaratis	NA20850	0.398806	0.036606	0.130337	0.001048	0.410127	0.002737	0.00001	0.020329
Gujaratis	NA20858	0.316769	0.061732	0.101514	0.005585	0.495992	0.002062	0.016337	0.00001
Gujaratis	NA20897	0.655206	0.033678	0.001428	0.000067	0.280321	0.010821	0.001211	0.017268
Gujaratis	NA20904	0.366772	0.045651	0.135101	0.005483	0.429766	0.00481	0.00001	0.012408
Gujaratis	NA21116	0.536159	0.024082	0.027079	0.00001	0.388664	0.002349	0.00001	0.021646
Gujaratis	NA21144	0.577581	0.020458	0.00001	0.00001	0.39129	0.00001	0.008079	0.002562
Gujaratis	NA20890	0.437517	0.026235	0.104814	0.005415	0.402468	0.005372	0.00001	0.018168
Gujaratis	NA21113	0.59278	0.034225	0.00001	0.002205	0.369924	0.00001	0.000836	0.00001
Gujaratis	NA21118	0.603144	0.019546	0.02129	0.00001	0.35598	0.00001	0.00001	0.00001
Gujaratis	NA21097	0.543442	0.027086	0.03181	0.00001	0.387854	0.00001	0.00001	0.009778
Gujaratis	NA20896	0.608707	0.012399	0.003525	0.00001	0.366261	0.004026	0.00001	0.005061
Gujaratis	NA20895	0.49279	0.042336	0.046393	0.000551	0.410254	0.000208	0.007458	0.00001
Gujaratis	NA20869	0.593339	0.019319	0.013004	0.00001	0.374298	0.00001	0.00001	0.00001
Gujaratis	NA20879	0.483222	0.045527	0.040525	0.001891	0.3977	0.00001	0.001546	0.029579
Gujaratis	NA20861	0.598085	0.008038	0.00001	0.00001	0.392478	0.000562	0.00001	0.000807
Gujaratis	NA20876	0.558145	0.014106	0.009498	0.00001	0.40835	0.002489	0.00001	0.007393
Gujaratis	NA20885	0.447458	0.026187	0.089353	0.00258	0.413045	0.00673	0.014636	0.00001
Gujaratis	NA20859	0.583603	0.031053	0.00025	0.00001	0.378428	0.00001	0.00001	0.006636
Gujaratis	NA20863	0.573515	0.00627	0.022392	0.007464	0.377297	0.00001	0.00001	0.013042
Gujaratis	NA21093	0.55779	0.01711	0.005101	0.028442	0.380326	0.00001	0.00001	0.01121
Gujaratis	NA21124	0.573564	0.016263	0.002638	0.00001	0.393916	0.00001	0.00001	0.013589
Gujaratis	NA21131	0.56795	0.027988	0.015376	0.000998	0.379656	0.00001	0.00001	0.008012
Gujaratis	NA21132	0.566922	0.014346	0.00001	0.012169	0.395881	0.00001	0.00001	0.010652
Brahmins_UP	177e	0.421076	0.032177	0.127323	0.00001	0.409566	0.00001	0.00001	0.009829
Brahmins_UP	BR008	0.44823	0.043515	0.101192	0.000066	0.386133	0.001753	0.00001	0.019102
Brahmins_UP	BR052	0.431799	0.010921	0.133328	0.00001	0.390957	0.006273	0.00001	0.026702
Brahmins_UP	BR084	0.44617	0.006533	0.151511	0.00214	0.378565	0.00001	0.001321	0.01375
Brahmins_UP	BRG001	0.470652	0.008801	0.116891	0.00001	0.379699	0.005458	0.00001	0.018478
Brahmins_UP	DEL007	0.595095	0.00001	0.055984	0.00001	0.330376	0.00001	0.00001	0.018505

Brahmins_UP	D238	0.496755	0.000876	0.131854	0.003659	0.355557	0.002508	0.00001	0.008781
Rajasthan	A105	0.463945	0.040906	0.061984	0.00001	0.416487	0.00001	0.00001	0.016648
Rajasthan	D442	0.570676	0.029475	0.02734	0.002165	0.351801	0.003512	0.00001	0.015021
Kshatriya	195	0.507577	0.033282	0.050794	0.001137	0.36948	0.005747	0.00001	0.031972
Kshatriya	198	0.485298	0.023115	0.082865	0.000202	0.390729	0.00001	0.013439	0.004341
Kshatriya	201	0.490344	0.043784	0.067082	0.00001	0.38051	0.00001	0.00001	0.018249
Kshatriya	199e	0.489448	0.00001	0.127899	0.001648	0.36457	0.00001	0.001169	0.015246
Kshatriya	204e	0.505807	0.005432	0.08824	0.00062	0.364787	0.003005	0.011941	0.020168
Kshatriya	208e	0.46835	0.00001	0.098882	0.005842	0.408266	0.001891	0.013015	0.003743
Kshatriya	209e	0.470119	0.026333	0.08103	0.00001	0.399703	0.002363	0.019264	0.001178
Harijan	A253	0.775675	0.00001	0.00001	0.00001	0.0906	0.013183	0.120501	0.00001
Harijan	A259	0.694701	0.00001	0.018489	0.00001	0.257757	0.002562	0.00001	0.02646
Harijan	A260	0.723175	0.000565	0.004422	0.00001	0.251966	0.00001	0.001158	0.018694
Harijan	A261	0.696306	0.00001	0.00001	0.00001	0.27452	0.006268	0.010909	0.011967
Harijan	A266	0.698536	0.00001	0.016839	0.00001	0.247054	0.001923	0.00001	0.035618
Harijan	A268	0.748476	0.005645	0.00001	0.00001	0.221155	0.001834	0.02286	0.00001
Harijan	evo_40	0.590337	0.00001	0.092354	0.00001	0.29832	0.00001	0.00001	0.018949
Harijan	evo_41	0.714932	0.004987	0.018607	0.00001	0.223189	0.014398	0.00001	0.023867
Harijan	evo_42	0.701505	0.001562	0.001844	0.00001	0.267021	0.00203	0.005213	0.020814
Harijan	evo_43	0.718941	0.008036	0.00001	0.00001	0.240963	0.00001	0.00001	0.03202
Dharkars	DH001	0.678621	0.00001	0.041814	0.00001	0.24173	0.00001	0.029701	0.008104
Dharkars	HA023	0.589389	0.00001	0.046362	0.00001	0.349879	0.00001	0.00001	0.01433
Dharkars	HA029	0.554438	0.016356	0.071988	0.004514	0.341249	0.00001	0.00001	0.011436
Dharkars	HA037	0.60157	0.010862	0.038974	0.00001	0.328006	0.00001	0.020558	0.00001
Dharkars	HA038	0.569678	0.00001	0.063247	0.00001	0.323242	0.01138	0.007727	0.024705
Dharkars	HA039	0.597462	0.00001	0.053621	0.00001	0.325425	0.00001	0.00001	0.023452
Dharkars	HA040	0.587891	0.001052	0.061988	0.00001	0.332077	0.00001	0.00001	0.016962
Dharkars	HA041	0.59223	0.00001	0.049996	0.00001	0.340666	0.00001	0.00001	0.017068
Dharkars	HA042	0.572939	0.00001	0.055299	0.00001	0.353192	0.003387	0.00001	0.015152
Dharkars	HA045	0.568532	0.000443	0.094931	0.00001	0.316437	0.002976	0.00567	0.011
Dharkars	HA048	0.579083	0.016053	0.021988	0.00001	0.361124	0.00001	0.009955	0.011777
Dusadh	A386	0.666486	0.00001	0.017312	0.00001	0.279875	0.005831	0.019264	0.011212
Dusadh	A387	0.661328	0.013508	0.000515	0.00001	0.288091	0.000589	0.018807	0.017152
Dusadh	A392	0.660312	0.00001	0.015202	0.00001	0.295563	0.007207	0.00001	0.021687
Dusadh	A394	0.64734	0.00001	0.01189	0.00001	0.30676	0.01295	0.00001	0.02103
Dusadh	A396	0.630981	0.038169	0.00001	0.004485	0.29139	0.001111	0.014532	0.019323
Dusadh	A397	0.659367	0.032447	0.00001	0.000608	0.283	0.003889	0.00001	0.020669
Dusadh	D494	0.600004	0.012417	0.035284	0.00001	0.312458	0.006294	0.018095	0.015438
Kanjars	A141	0.62146	0.00001	0.060949	0.00001	0.306983	0.00001	0.010567	0.00001
Kanjars	A143	0.582537	0.003805	0.037144	0.00001	0.355986	0.001535	0.00001	0.018974
Kanjars	evo_33	0.565838	0.00001	0.041074	0.00001	0.365918	0.010678	0.003722	0.01275
Kanjars	evo_34	0.569209	0.00001	0.061612	0.002718	0.338758	0.00001	0.00001	0.027673
Kanjars	evo_35	0.590044	0.00001	0.058466	0.00001	0.325478	0.00001	0.00001	0.025973
Kanjars	evo_36	0.631234	0.00001	0.054594	0.00001	0.302898	0.00001	0.011234	0.00001
Kanjars	evo_37	0.614242	0.00001	0.039826	0.00001	0.33657	0.003366	0.00001	0.005967
Kanjars	evo_38	0.611505	0.00001	0.041653	0.00001	0.316005	0.00107	0.006925	0.022822
Kol	evo_32	0.722213	0.00001	0.004516	0.00001	0.247676	0.00001	0.00001	0.025555
Kol	288	0.569958	0.013055	0.034691	0.00001	0.363842	0.004689	0.00001	0.013746
Kol	290	0.676037	0.00001	0.011543	0.00001	0.278356	0.006791	0.008881	0.018371
Kol	298	0.551607	0.020743	0.025705	0.00001	0.358326	0.00001	0.034713	0.008885
Kol	306	0.705548	0.00001	0.012972	0.00001	0.273493	0.00124	0.00001	0.006718
Kol	314	0.603913	0.008657	0.05069	0.000012	0.319339	0.000444	0.016935	0.00001
Kol	319	0.596795	0.012836	0.040029	0.00001	0.330272	0.00001	0.011557	0.008493
Kol	323	0.695731	0.00001	0.01177	0.00001	0.239478	0.005283	0.029875	0.017844
Kol	332	0.673223	0.00001	0.026455	0.00001	0.28023	0.002131	0.001235	0.016705
Kol	296e	0.611957	0.00001	0.039661	0.000335	0.32799	0.00001	0.008626	0.011411

Kol	308e	0.727139	0.002505	0.017571	0.00001	0.23408	0.00001	0.00001	0.018674
Kol	309e	0.558592	0.032126	0.012699	0.00001	0.366541	0.001876	0.00001	0.028147
Kol	310e	0.728815	0.00001	0.00001	0.00001	0.243625	0.005069	0.009041	0.013421
Kol	328e	0.701164	0.00001	0.016162	0.00001	0.258784	0.003109	0.020751	0.00001
Kol	330e	0.710623	0.00001	0.00001	0.00001	0.271586	0.00244	0.00001	0.015311
Kol	335e	0.373338	0.00001	0.00001	0.00001	0.185547	0.015351	0.155565	0.270169
UP_Low_Caste	evo_10	0.577135	0.011686	0.01719	0.00001	0.365729	0.000648	0.016402	0.011201
UP_Low_Caste	Z217	0.703944	0.00001	0.00001	0.00001	0.249636	0.005908	0.002216	0.038267
UP_Low_Caste	Z218	0.633831	0.01806	0.037219	0.00001	0.280922	0.000862	0.00001	0.029086
UP_Low_Caste	Z220	0.570267	0.00001	0.084854	0.00001	0.316589	0.00001	0.022243	0.006018
UP_Low_Caste	Z224	0.556933	0.033019	0.010813	0.00001	0.377469	0.005984	0.00001	0.015762
UP_Low_Caste	Z225	0.662176	0.021347	0.00001	0.000332	0.281784	0.003378	0.00001	0.030963
Tharus	D254	0.600137	0.00001	0.03972	0.00001	0.33272	0.004651	0.00001	0.022742
Tharus	D260	0.551714	0.013836	0.047594	0.003759	0.341354	0.001634	0.024649	0.015461
Transitional	D201	0.758514	0.00001	0.00001	0.00001	0.00001	0.020514	0.220922	0.00001
Transitional	ORI23	0.751372	0.00001	0.00001	0.00001	0.00001	0.023434	0.225144	0.00001
Gond	G53	0.822351	0.00001	0.00001	0.00001	0.001333	0.02268	0.153597	0.00001
Gond	GONC1	0.476746	0.037331	0.07917	0.000447	0.387668	0.005931	0.00001	0.012698
Gond	GONC5	0.760558	0.00001	0.00001	0.00001	0.127343	0.007742	0.104318	0.00001
Gond	GONC8	0.664987	0.00001	0.017577	0.00001	0.18522	0.016805	0.115381	0.00001
Chenchus	CHEND85	0.65942	0.023774	0.003503	0.003496	0.24533	0.002656	0.03796	0.023862
Chenchus	CHEND90	0.688581	0.00139	0.00001	0.00001	0.234949	0.009973	0.029929	0.035157
Chenchus	CHEND95	0.65329	0.01689	0.006144	0.00001	0.227505	0.008479	0.033241	0.05444
Chenchus	CHEND96	0.667667	0.021812	0.00001	0.00001	0.266938	0.004671	0.00001	0.038882
Velmas	VELZ260	0.578232	0.026315	0.00001	0.00001	0.393338	0.001566	0.00001	0.000519
Velmas	VELZ264	0.641766	0.025118	0.00001	0.00001	0.300528	0.005828	0.014906	0.011834
Velmas	VELZ265	0.600067	0.021779	0.00001	0.00001	0.370009	0.00001	0.008105	0.00001
Velmas	VELZ267	0.606586	0.035693	0.00001	0.000221	0.341348	0.001855	0.014277	0.00001
Velmas	VELZ271	0.61867	0.031693	0.00001	0.00001	0.339384	0.003861	0.00001	0.006362
Velmas	VELZ272	0.592477	0.035306	0.00001	0.000235	0.358204	0.008236	0.005522	0.00001
Velmas	VELZ274	0.622557	0.013009	0.00001	0.00001	0.346	0.00001	0.008125	0.010278
Velmas	VELZ275	0.599726	0.030413	0.00001	0.00001	0.353513	0.004666	0.00001	0.011652
Velmas	VELZ277	0.597396	0.027334	0.00001	0.00001	0.356994	0.007682	0.009956	0.000618
Velmas	VELZ281	0.628126	0.029824	0.00001	0.00001	0.334289	0.00001	0.003539	0.004192
Hakkipikki	HLKP241	0.755938	0.016608	0.00001	0.00001	0.184105	0.009396	0.00001	0.033923
Hakkipikki	HLKP243	0.747064	0.031819	0.00001	0.00001	0.173962	0.007101	0.007639	0.032396
Hakkipikki	HLKP245	0.761852	0.004243	0.00001	0.00001	0.208714	0.001091	0.00001	0.02407
Hakkipikki	HLKP252	0.696946	0.018519	0.000726	0.005872	0.249239	0.005464	0.002062	0.021172
North_Kannadi	KNTK385	0.79509	0.031975	0.00001	0.00001	0.153885	0.00001	0.00001	0.019011
North_Kannadi	KNTK386	0.802691	0.028167	0.00001	0.00001	0.146033	0.003281	0.00001	0.019799
North_Kannadi	KNTK388	0.795537	0.03065	0.00001	0.000123	0.14333	0.000648	0.00001	0.029692
North_Kannadi	KNTK390	0.80887	0.02987	0.00001	0.00001	0.147779	0.001588	0.00001	0.011862
North_Kannadi	KNTK392	0.732096	0.000424	0.00001	0.00001	0.211721	0.011511	0.015466	0.028762
North_Kannadi	KNTK394	0.779634	0.018024	0.00001	0.00001	0.174683	0.007724	0.010994	0.008919
North_Kannadi	KNTK397	0.80759	0.00001	0.00001	0.00001	0.144662	0.00622	0.025734	0.015765
North_Kannadi	KNTK400	0.676012	0.019192	0.000612	0.005599	0.276658	0.004599	0.001933	0.015395
North_Kannadi	KNTK408	0.581729	0.021646	0.036505	0.001236	0.340538	0.001662	0.00001	0.016674
Malayan	A382	0.839438	0.00001	0.00001	0.0083	0.067171	0.02483	0.035252	0.024989
Malayan	MLYA383	0.793717	0.004975	0.00001	0.000911	0.100669	0.018243	0.046349	0.035125
Paniya	D36	0.758071	0.00001	0.00001	0.002175	0.191531	0.014245	0.033949	0.00001
Paniya	PNYD1	0.877639	0.00001	0.00001	0.00001	0.00001	0.034699	0.049927	0.037695
Paniya	PNYD3	0.89643	0.00001	0.00001	0.00001	0.00001	0.022006	0.037717	0.043807
Paniya	PNYD9	0.885742	0.00001	0.00001	0.001252	0.00001	0.036289	0.016955	0.059732
Sakilli	SAKD60	0.713	0.014769	0.00001	0.002538	0.244816	0.008184	0.016673	0.00001
Sakilli	SAKD64	0.73016	0.002619	0.00001	0.000735	0.231618	0.012764	0.013142	0.008952
Sakilli	SAKD72	0.716287	0.016775	0.00001	0.004626	0.238428	0.00001	0.00001	0.023854

Sakilli	SAKD75	0.732827	0.006983	0.00001	0.002091	0.226419	0.012192	0.012713	0.006766
Kurumba	KUR1	0.622347	0.016258	0.00001	0.00001	0.333877	0.005124	0.001809	0.020565
Kurumba	KUR2	0.624606	0.024424	0.00001	0.00001	0.335008	0.007885	0.00001	0.008047
Kurumba	KUR3	0.690805	0.029906	0.00001	0.00001	0.258956	0.006081	0.005082	0.009151
Kurumba	KUR4	0.694958	0.026472	0.001441	0.00001	0.25822	0.007518	0.000755	0.010626
TN_Low_Caste	TN18	0.647349	0.016814	0.00001	0.00001	0.300772	0.015855	0.01918	0.00001
TN_Low_Caste	TN26	0.602734	0.04649	0.00001	0.00001	0.335606	0.003362	0.011777	0.00001
Piramalai_Kalla	PK6415	0.666082	0.008516	0.00001	0.00001	0.309708	0.006911	0.00001	0.008753
Piramalai_Kalla	PK6422	0.659749	0.011788	0.00001	0.00001	0.300431	0.010687	0.00001	0.017315
Piramalai_Kalla	PK6442	0.650905	0.007267	0.00001	0.00001	0.308642	0.014539	0.017739	0.000886
Piramalai_Kalla	PK6458	0.649489	0.01317	0.00001	0.000861	0.303041	0.00001	0.02946	0.00396
Piramalai_Kalla	PK6459	0.676766	0.016899	0.00001	0.00001	0.281112	0.010457	0.014736	0.00001
Piramalai_Kalla	PK6468	0.70184	0.00001	0.00001	0.003568	0.273481	0.0056	0.004623	0.010868
Piramalai_Kalla	PK6490	0.6623	0.022301	0.00001	0.003083	0.276838	0.007041	0.00001	0.028418
Piramalai_Kalla	PK6491	0.6734	0.016505	0.00001	0.005781	0.279444	0.011324	0.00001	0.013526
Pulliyar	PULD151	0.908866	0.039648	0.00001	0.00001	0.00001	0.010411	0.00001	0.041036
Pulliyar	PULD160	0.907904	0.022082	0.020973	0.00001	0.00001	0.004084	0.00001	0.044927
Pulliyar	PULD162	0.894151	0.059225	0.00001	0.00001	0.00001	0.009117	0.00001	0.037467
Pulliyar	PULD171	0.914044	0.040511	0.00001	0.00001	0.00001	0.009047	0.00001	0.036358
Pulliyar	PULD172	0.909104	0.047365	0.00001	0.00001	0.00001	0.006571	0.00001	0.03692
North Munda	226	0.727463	0.00001	0.000584	0.00001	0.109795	0.014858	0.147271	0.00001
North Munda	ASUR350	0.76364	0.00001	0.00001	0.00001	0.00001	0.016012	0.220297	0.00001
North Munda	480	0.716793	0.00001	0.00001	0.00001	0.00001	0.019552	0.263606	0.00001
North Munda	HO434	0.723471	0.00001	0.00001	0.00001	0.00001	0.023278	0.2532	0.00001
North Munda	HO438	0.705766	0.00001	0.00001	0.002999	0.003838	0.029709	0.257657	0.00001
North Munda	HO446	0.731308	0.00001	0.00001	0.00001	0.00001	0.028913	0.239729	0.00001
North Munda	HO470	0.739382	0.00001	0.00001	0.00001	0.00001	0.013765	0.246803	0.00001
North Munda	Mawasi1	0.736601	0.00001	0.00001	0.00001	0.021777	0.019692	0.22189	0.00001
North Munda	G22	0.774919	0.00001	0.00001	0.00001	0.018723	0.021397	0.184921	0.00001
South Munda	ORI34	0.650292	0.00001	0.00001	0.00001	0.00001	0.023851	0.325807	0.00001
South Munda	ORI35	0.640507	0.00001	0.00001	0.00001	0.00001	0.023533	0.33591	0.00001
South Munda	ORI36	0.651227	0.00001	0.00001	0.00001	0.00001	0.012655	0.336067	0.00001
South Munda	ORI37	0.653517	0.00001	0.00001	0.00001	0.00001	0.013175	0.333258	0.00001
South Munda	ORI88	0.661081	0.00001	0.00001	0.00001	0.00001	0.033409	0.30546	0.00001
South Munda	A41	0.63835	0.00001	0.00001	0.00001	0.00001	0.021129	0.340471	0.00001
South Munda	JUANA48	0.645411	0.00001	0.00001	0.00001	0.00001	0.028806	0.325733	0.00001
South Munda	KH1	0.701119	0.00001	0.00001	0.00001	0.00001	0.030142	0.26869	0.00001
South Munda	KH15	0.711004	0.00001	0.00001	0.001444	0.00001	0.019693	0.267819	0.00001
South Munda	ORI93	0.694994	0.00001	0.00001	0.00001	0.00001	0.018691	0.286265	0.00001
South Munda	SAVOR105	0.729484	0.00001	0.00001	0.00001	0.00001	0.027086	0.24338	0.00001
Khasi	KHL4	0.276738	0.00001	0.006889	0.00001	0.050643	0.008578	0.483917	0.173215
Khasi	KHP3	0.312234	0.00537	0.027779	0.001075	0.05203	0.007865	0.465936	0.127712
Khasi	KHP4	0.351693	0.00001	0.029585	0.002076	0.089689	0.010665	0.404234	0.112048
Garo	GA1	0.184997	0.00001	0.00001	0.00001	0.00001	0.012767	0.522927	0.279269
Garo	GA13	0.176632	0.00001	0.00001	0.00001	0.00001	0.007101	0.553943	0.262284
Garo	GA23	0.198309	0.00001	0.004229	0.00643	0.00001	0.010937	0.533991	0.246083
Garo	GA24	0.182834	0.00001	0.00001	0.00001	0.00001	0.010392	0.547355	0.259379
Naga	NAG129	0.00001	0.00001	0.00001	0.00001	0.00001	0.003611	0.474229	0.52211
Naga	NAG131	0.00001	0.00001	0.00001	0.00001	0.00001	0.006474	0.427407	0.56607
Naga	NAG133	0.00001	0.00001	0.00001	0.000013	0.00001	0.010634	0.440492	0.548821
Naga	NAG134	0.006555	0.00001	0.00001	0.00001	0.00001	0.00001	0.417765	0.57563
Burmese	bumaBR50	0.166751	0.000012	0.00001	0.00001	0.047877	0.001836	0.5435	0.240005
Burmese	bumaBR55	0.130712	0.000068	0.00001	0.000308	0.012833	0.010149	0.544032	0.301888
Burmese	bumaBR64	0.158397	0.00001	0.00001	0.001936	0.043115	0.008471	0.528836	0.259225
Burmese	bumaBR69	0.177945	0.004878	0.00001	0.00001	0.041493	0.009898	0.519288	0.246478
Burmese	bumaBR78	0.175466	0.00001	0.00001	0.003812	0.042872	0.007902	0.527628	0.2423

Burmese	bumaBR81	0.223087	0.009556	0.006143	0.00001	0.056033	0.006554	0.522524	0.176094
Burmese	bumaBR83	0.091329	0.00001	0.000012	0.00001	0.02248	0.008283	0.588607	0.289268
Burmese	bumaBR84	0.085849	0.00001	0.00001	0.00001	0.00001	0.007699	0.541868	0.364543
Burmese	bumaBR91	0.30015	0.024119	0.062732	0.00001	0.220395	0.006719	0.274484	0.111392
Burmese	bumaBR54	0.161322	0.002986	0.00637	0.005878	0.023169	0.013429	0.539453	0.247393
Burmese	bumaBR56	0.171724	0.001267	0.008839	0.001264	0.030396	0.002696	0.527788	0.256026
Burmese	bumaBR62	0.178474	0.001667	0.007622	0.000468	0.034326	0.009509	0.520385	0.247549
Burmese	bumaBR68	0.170915	0.010445	0.010198	0.002408	0.025364	0.008964	0.496337	0.275369
Burmese	bumaBR98	0.08531	0.00001	0.000011	0.00001	0.00001	0.011475	0.572849	0.330324
Burmese	bumaBR110	0.180995	0.008414	0.00082	0.00001	0.044423	0.010552	0.514355	0.24043
Cambodians	HGDP00711	0.14252	0.002856	0.003717	0.002845	0.018376	0.015102	0.814574	0.00001
Cambodians	HGDP00712	0.024879	0.00001	0.00001	0.00001	0.00001	0.006732	0.775417	0.192931
Cambodians	HGDP00713	0.161524	0.00001	0.009439	0.00001	0.012875	0.013572	0.80256	0.00001
Cambodians	HGDP00714	0.086744	0.012175	0.003703	0.00001	0.008031	0.0148	0.822814	0.051722
Cambodians	HGDP00715	0.149683	0.003966	0.030615	0.00001	0.014558	0.016074	0.785085	0.00001
Cambodians	HGDP00716	0.101727	0.001298	0.00001	0.00001	0.00001	0.011663	0.823322	0.06196
Cambodians	HGDP00717	0.131819	0.00001	0.016724	0.003036	0.003133	0.018026	0.827242	0.00001
Cambodians	HGDP00719	0.123461	0.001245	0.006791	0.00001	0.008569	0.010487	0.807351	0.042087
Cambodians	HGDP00720	0.134035	0.009315	0.00001	0.00001	0.028566	0.020754	0.8073	0.00001
Cambodians	HGDP00721	0.154135	0.013347	0.00001	0.00001	0.017345	0.025942	0.789201	0.00001
Dai	HGDP01307	0.00001	0.00001	0.00001	0.00001	0.00001	0.006064	0.963065	0.030821
Dai	HGDP01308	0.00001	0.00001	0.00001	0.00001	0.00001	0.00001	0.974995	0.024945
Dai	HGDP01309	0.00001	0.00001	0.00001	0.00001	0.00001	0.000948	0.998992	0.00001
Dai	HGDP01310	0.00001	0.00001	0.00001	0.00001	0.00001	0.00074	0.9992	0.00001
Dai	HGDP01311	0.00001	0.00001	0.00001	0.00001	0.00001	0.006607	0.982338	0.011005
Dai	HGDP01312	0.00001	0.00001	0.00001	0.00001	0.00001	0.00001	0.99993	0.00001
Dai	HGDP01313	0.00001	0.00001	0.00001	0.00001	0.00001	0.002914	0.973497	0.023539
Dai	HGDP01314	0.00001	0.00001	0.00001	0.00001	0.00001	0.003109	0.979622	0.017219
Dai	HGDP01315	0.000015	0.00001	0.00001	0.00001	0.00001	0.010761	0.980246	0.008939
Dai	HGDP01316	0.00001	0.00001	0.00001	0.00001	0.00001	0.001748	0.985421	0.012782
Lahu	HGDP01317	0.000013	0.00001	0.00001	0.00001	0.00001	0.000071	0.93019	0.069686
Lahu	HGDP01318	0.00001	0.00001	0.00001	0.00001	0.00001	0.00001	0.99993	0.00001
Lahu	HGDP01319	0.009419	0.00001	0.00001	0.00001	0.00001	0.009504	0.896283	0.084755
Lahu	HGDP01320	0.002314	0.00001	0.00001	0.00001	0.00001	0.002359	0.995277	0.00001
Lahu	HGDP01321	0.001421	0.00001	0.00001	0.00001	0.00001	0.003426	0.995102	0.00001
Lahu	HGDP01322	0.00001	0.00001	0.00001	0.00001	0.00001	0.00001	0.99993	0.00001
Lahu	HGDP01323	0.006671	0.00001	0.00001	0.00001	0.00001	0.00281	0.923353	0.067126
Lahu	HGDP01326	0.00001	0.00001	0.00001	0.00001	0.00001	0.009821	0.884106	0.106023
Miaozu	HGDP01189	0.00001	0.000011	0.00001	0.00001	0.00001	0.00001	0.744563	0.255376
Miaozu	HGDP01190	0.00001	0.00001	0.00001	0.000013	0.00001	0.00001	0.724569	0.275368
Miaozu	HGDP01191	0.00001	0.00001	0.00001	0.00001	0.00001	0.00001	0.743976	0.255964
Miaozu	HGDP01192	0.00001	0.00001	0.00001	0.00001	0.00001	0.00001	0.757932	0.242008
Miaozu	HGDP01193	0.00001	0.00001	0.00001	0.00001	0.00001	0.00001	0.757513	0.242427
Miaozu	HGDP01194	0.00001	0.00001	0.00001	0.00001	0.00001	0.00001	0.765457	0.234483
Miaozu	HGDP01195	0.000018	0.00001	0.00001	0.00001	0.000013	0.00001	0.735946	0.263984
Miaozu	HGDP01196	0.000012	0.00001	0.00001	0.00001	0.00001	0.00001	0.729092	0.270846
Miaozu	HGDP01197	0.00001	0.000015	0.00001	0.00001	0.00001	0.00001	0.735014	0.264921
Miaozu	HGDP01198	0.000012	0.00001	0.00001	0.00001	0.00001	0.00001	0.754028	0.24591
Naxi	HGDP01337	0.008364	0.00001	0.00001	0.00001	0.00001	0.00001	0.549785	0.4418
Naxi	HGDP01338	0.00001	0.00001	0.00001	0.00001	0.00001	0.003058	0.53818	0.458712
Naxi	HGDP01339	0.00001	0.00001	0.00001	0.00001	0.00001	0.00001	0.523888	0.476052
Naxi	HGDP01340	0.001251	0.00001	0.00001	0.00001	0.00001	0.0076	0.500858	0.490252
Naxi	HGDP01341	0.000986	0.00001	0.00001	0.00001	0.00001	0.001598	0.536653	0.460723
Naxi	HGDP01342	0.00001	0.00001	0.00001	0.00001	0.00001	0.00001	0.520817	0.479123
Naxi	HGDP01345	0.00001	0.00001	0.00001	0.00001	0.00001	0.00001	0.512707	0.487233
Naxi	HGDP01346	0.01324	0.00001	0.00001	0.00001	0.00001	0.010151	0.547111	0.429459

She	HGDP01327	0.00001	0.00001	0.00001	0.00001	0.00001	0.00001	0.813998	0.185942
She	HGDP01328	0.000017	0.00001	0.00001	0.00001	0.00001	0.00001	0.722733	0.2772
She	HGDP01329	0.00001	0.00001	0.00001	0.00001	0.00001	0.00001	0.741019	0.258921
She	HGDP01330	0.00001	0.00001	0.000013	0.00001	0.00001	0.00001	0.801641	0.198296
She	HGDP01331	0.00001	0.000018	0.00001	0.00001	0.00001	0.00001	0.761344	0.238588
She	HGDP01332	0.00001	0.00001	0.00001	0.00001	0.00001	0.00001	0.766141	0.233799
She	HGDP01333	0.00001	0.000013	0.00001	0.000017	0.00001	0.00001	0.762391	0.23754
She	HGDP01334	0.00001	0.00001	0.00001	0.00001	0.00001	0.00001	0.764276	0.235664
She	HGDP01335	0.00001	0.00001	0.00001	0.00001	0.00001	0.00001	0.796013	0.203927
She	HGDP01336	0.000013	0.00001	0.00001	0.00001	0.00001	0.00001	0.762675	0.237261
Yizu	HGDP01179	0.00001	0.00001	0.00001	0.00001	0.00001	0.001036	0.559835	0.439079
Yizu	HGDP01180	0.000016	0.00001	0.00001	0.00001	0.00001	0.001821	0.521989	0.476133
Yizu	HGDP01181	0.000012	0.00001	0.00001	0.00001	0.00001	0.009693	0.529125	0.461131
Yizu	HGDP01182	0.001148	0.00001	0.00001	0.00001	0.00001	0.005873	0.419049	0.57389
Yizu	HGDP01183	0.014504	0.00001	0.00001	0.00001	0.00001	0.00667	0.508386	0.470401
Yizu	HGDP01184	0.005125	0.00001	0.00001	0.00001	0.00001	0.00001	0.538178	0.456647
Yizu	HGDP01185	0.00001	0.00001	0.00001	0.00001	0.00001	0.000052	0.568856	0.431043
Yizu	HGDP01186	0.00001	0.00001	0.00001	0.00001	0.00001	0.007694	0.583076	0.40918
Yizu	HGDP01187	0.00001	0.00001	0.00001	0.00001	0.00001	0.00001	0.63202	0.36792
Yizu	HGDP01188	0.00001	0.000011	0.00001	0.00001	0.00001	0.007872	0.551676	0.440401
Tujia	HGDP01095	0.00001	0.00001	0.00001	0.00001	0.00001	0.00001	0.707614	0.292326
Tujia	HGDP01096	0.00001	0.00001	0.000016	0.00001	0.00001	0.00001	0.656448	0.343485
Tujia	HGDP01097	0.000013	0.00001	0.00001	0.00001	0.00001	0.00001	0.697835	0.302101
Tujia	HGDP01098	0.00001	0.00001	0.00001	0.00001	0.00001	0.00001	0.698795	0.301145
Tujia	HGDP01099	0.00001	0.00001	0.00001	0.00001	0.00001	0.00001	0.605	0.39494
Tujia	HGDP01100	0.00001	0.00001	0.00001	0.000011	0.00001	0.00001	0.626806	0.373133
Tujia	HGDP01101	0.00001	0.00001	0.00001	0.00001	0.00001	0.00001	0.689773	0.310167
Tujia	HGDP01102	0.00001	0.00001	0.00001	0.00001	0.00001	0.00001	0.636992	0.362948
Tujia	HGDP01103	0.00001	0.00001	0.00001	0.00001	0.00001	0.00001	0.727144	0.272796
Tujia	HGDP01104	0.00001	0.00001	0.00001	0.00001	0.00001	0.00001	0.611763	0.388177
Han	HGDP00774	0.00001	0.00001	0.000011	0.00001	0.00001	0.00001	0.746151	0.253788
Han	HGDP00775	0.00001	0.00001	0.00001	0.00001	0.00001	0.00001	0.581732	0.418208
Han	HGDP00776	0.00001	0.00001	0.00001	0.00001	0.00001	0.00001	0.812501	0.187439
Han	HGDP00777	0.00001	0.00001	0.00001	0.00001	0.00001	0.00001	0.489855	0.510085
Han	HGDP00778	0.000015	0.00001	0.000017	0.00001	0.00001	0.00001	0.696219	0.30371
Han	HGDP00779	0.00001	0.00001	0.000014	0.000011	0.00001	0.00001	0.583191	0.416744
Han	HGDP00780	0.00001	0.00001	0.00001	0.00001	0.00001	0.00001	0.731775	0.268165
Han	HGDP00781	0.00001	0.00001	0.00001	0.00001	0.00001	0.00001	0.825786	0.174154
Han	HGDP00782	0.00001	0.00001	0.00001	0.000014	0.00001	0.00001	0.596716	0.40322
Han	HGDP00783	0.00001	0.00001	0.00001	0.00001	0.00001	0.00001	0.533959	0.465981
Han	HGDP00784	0.00001	0.000014	0.00001	0.00001	0.00001	0.00001	0.710947	0.288989
Han	HGDP00785	0.000014	0.00001	0.00001	0.000011	0.00001	0.00001	0.830373	0.169562
Han	HGDP00786	0.00001	0.00001	0.000011	0.000013	0.00001	0.00001	0.680487	0.31945
Han	HGDP00811	0.00001	0.00001	0.00001	0.00001	0.00001	0.00001	0.69127	0.30867
Han	HGDP00812	0.000012	0.00001	0.00001	0.00001	0.00001	0.00001	0.833826	0.166112
Han	HGDP00813	0.00001	0.00001	0.00001	0.00001	0.00001	0.00001	0.718745	0.281195
Han	HGDP00814	0.00001	0.00001	0.00001	0.00001	0.00001	0.00001	0.551364	0.448576
Han	HGDP00815	0.00001	0.00001	0.00001	0.00001	0.000011	0.00001	0.591173	0.408765
Han	HGDP00817	0.000012	0.00001	0.00001	0.00001	0.00001	0.00001	0.811469	0.188469
Han	HGDP00818	0.00001	0.00001	0.00001	0.00001	0.00001	0.00001	0.656631	0.343309
Han	HGDP00819	0.00001	0.00001	0.00001	0.00001	0.00001	0.00001	0.807311	0.192629
Han	HGDP00820	0.000011	0.00001	0.00001	0.00001	0.00001	0.00001	0.788761	0.211178
Tu	HGDP01347	0.016924	0.00001	0.009396	0.001159	0.042062	0.004471	0.38655	0.539427
Tu	HGDP01348	0.017054	0.010275	0.036133	0.002932	0.020201	0.00001	0.386352	0.527044
Tu	HGDP01349	0.004475	0.010168	0.00001	0.00001	0.026259	0.001304	0.400956	0.556819
Tu	HGDP01350	0.010067	0.017222	0.017015	0.00001	0.046491	0.004794	0.386418	0.517983

Tu	HGDP01351	0.013483	0.006999	0.01785	0.00001	0.036282	0.000447	0.407686	0.517242
Tu	HGDP01352	0.009257	0.005282	0.012181	0.00001	0.030196	0.00001	0.377079	0.565986
Tu	HGDP01353	0.00581	0.003768	0.043693	0.00001	0.034642	0.001699	0.40779	0.502588
Tu	HGDP01354	0.00001	0.000937	0.008981	0.00001	0.033017	0.00001	0.414208	0.542826
Tu	HGDP01355	0.012978	0.00001	0.018248	0.001249	0.031774	0.00001	0.413956	0.521775
Tu	HGDP01356	0.003944	0.004071	0.020314	0.001939	0.041846	0.00962	0.396626	0.521642
Xibo	HGDP01243	0.00001	0.00772	0.152647	0.00001	0.061357	0.004541	0.217749	0.555967
Xibo	HGDP01244	0.00001	0.00001	0.005813	0.00001	0.028409	0.00001	0.257194	0.708544
Xibo	HGDP01245	0.00001	0.00001	0.00001	0.00001	0.018606	0.00001	0.258447	0.722897
Xibo	HGDP01246	0.00001	0.000017	0.00001	0.00001	0.008395	0.00001	0.274233	0.717315
Xibo	HGDP01247	0.00001	0.016493	0.066926	0.00001	0.025528	0.00001	0.31273	0.578292
Xibo	HGDP01248	0.00001	0.00001	0.033207	0.00001	0.029428	0.001172	0.258656	0.677507
Xibo	HGDP01249	0.00001	0.00001	0.00001	0.00001	0.016053	0.00001	0.314822	0.669075
Xibo	HGDP01250	0.00001	0.016905	0.000013	0.00001	0.024144	0.00001	0.268047	0.690861
Xibo	HGDP01251	0.00001	0.00001	0.00001	0.00001	0.014397	0.006286	0.311178	0.668099
Daur	HGDP01213	0.00001	0.00001	0.00001	0.00001	0.00001	0.00001	0.08384	0.9161
Daur	HGDP01214	0.00001	0.007828	0.00001	0.00001	0.036094	0.00001	0.175859	0.780179
Daur	HGDP01215	0.00001	0.00001	0.003568	0.00001	0.030005	0.002566	0.159739	0.804092
Daur	HGDP01216	0.009348	0.00001	0.00001	0.00001	0.005306	0.008867	0.115745	0.860704
Daur	HGDP01217	0.00001	0.008273	0.00001	0.00001	0.021852	0.00001	0.135985	0.83385
Daur	HGDP01218	0.00001	0.000815	0.00001	0.00001	0.012607	0.004938	0.104484	0.877125
Daur	HGDP01220	0.00001	0.00001	0.00001	0.00001	0.011165	0.00001	0.106955	0.881831
Daur	HGDP01221	0.00001	0.00001	0.00001	0.00001	0.00001	0.00001	0.117458	0.882482
Daur	HGDP01222	0.00001	0.000117	0.00001	0.00001	0.00001	0.00001	0.115291	0.884542
Hezhen	HGDP01234	0.00001	0.00001	0.00001	0.00001	0.00001	0.00001	0.00001	0.99993
Hezhen	HGDP01235	0.00001	0.00001	0.00001	0.00001	0.00001	0.00001	0.218099	0.781841
Hezhen	HGDP01236	0.00001	0.00001	0.00001	0.00001	0.00001	0.00001	0.25425	0.74569
Hezhen	HGDP01237	0.00001	0.00001	0.00001	0.00001	0.00001	0.00001	0.00001	0.99993
Hezhen	HGDP01238	0.00001	0.00001	0.00001	0.00001	0.024714	0.00001	0.217638	0.757597
Hezhen	HGDP01239	0.00001	0.00001	0.00001	0.00001	0.001993	0.004731	0.259837	0.733398
Hezhen	HGDP01240	0.00001	0.00001	0.00001	0.00001	0.00001	0.00001	0.00001	0.99993
Hezhen	HGDP01241	0.00001	0.00001	0.00001	0.00001	0.00001	0.000917	0.198398	0.800635
Hezhen	HGDP01242	0.00001	0.00001	0.00001	0.00001	0.00001	0.001169	0.109314	0.889466
Oroqens	HGDP01203	0.00001	0.00001	0.00001	0.00001	0.00001	0.00001	0.00001	0.99993
Oroqens	HGDP01204	0.00001	0.00001	0.000011	0.00001	0.00001	0.00001	0.156323	0.843615
Oroqens	HGDP01205	0.00001	0.00001	0.011182	0.00001	0.004888	0.001385	0.041194	0.941321
Oroqens	HGDP01206	0.00001	0.00001	0.00001	0.00001	0.00001	0.00001	0.00001	0.99993
Oroqens	HGDP01207	0.00001	0.00001	0.009715	0.00001	0.00001	0.00001	0.047096	0.943139
Oroqens	HGDP01208	0.00001	0.00001	0.000011	0.00001	0.00001	0.00001	0.122126	0.877812
Oroqens	HGDP01209	0.00001	0.00001	0.00001	0.00001	0.00001	0.007072	0.00001	0.992868
Oroqens	HGDP01211	0.00001	0.00001	0.00001	0.00001	0.00001	0.00001	0.00001	0.99993
Oroqens	HGDP01212	0.00874	0.00001	0.032104	0.00001	0.00001	0.00001	0.165982	0.793134
Mongola	HGDP01223	0.00001	0.00263	0.013282	0.00001	0.023336	0.00001	0.274855	0.685868
Mongola	HGDP01224	0.00001	0.00001	0.00001	0.00001	0.029353	0.00001	0.32572	0.644877
Mongola	HGDP01225	0.00001	0.001953	0.00001	0.00001	0.023994	0.00001	0.35427	0.619743
Mongola	HGDP01226	0.008782	0.00001	0.06196	0.00001	0.055021	0.007054	0.00001	0.867153
Mongola	HGDP01227	0.00001	0.00001	0.042438	0.00001	0.015451	0.00001	0.334053	0.608018
Mongola	HGDP01228	0.00001	0.00001	0.030745	0.00001	0.01401	0.00001	0.322944	0.632261
Mongola	HGDP01229	0.017937	0.00001	0.064473	0.00001	0.051073	0.00103	0.018382	0.847084
Mongola	HGDP01230	0.004991	0.00001	0.042744	0.00001	0.059199	0.005422	0.027725	0.859899
Mongola	HGDP01231	0.00001	0.00001	0.017602	0.00001	0.00001	0.00001	0.299208	0.68314
Mongola	HGDP01232	0.00001	0.009634	0.00001	0.002974	0.031795	0.00001	0.364144	0.591423
Japanese	HGDP00747	0.00001	0.00001	0.00001	0.00001	0.00001	0.00001	0.381885	0.618055
Japanese	HGDP00748	0.000017	0.00001	0.00001	0.00001	0.00001	0.00001	0.386492	0.613441
Japanese	HGDP00749	0.00001	0.00001	0.00001	0.00001	0.00001	0.00001	0.376456	0.623484
Japanese	HGDP00750	0.00001	0.00001	0.00001	0.00001	0.00001	0.00001	0.388308	0.611632

Japanese	HGDP00751	0.00001	0.00001	0.00001	0.00001	0.00001	0.009298	0.400219	0.590432
Japanese	HGDP00752	0.00001	0.00001	0.00001	0.00001	0.00001	0.00001	0.395129	0.604811
Japanese	HGDP00753	0.00001	0.00001	0.00001	0.00001	0.00001	0.00001	0.392108	0.607832
Japanese	HGDP00755	0.00001	0.00001	0.00001	0.00001	0.00001	0.00001	0.395442	0.604498
Japanese	HGDP00756	0.00001	0.00001	0.00001	0.00001	0.00001	0.009585	0.363875	0.62649
Japanese	HGDP00757	0.000019	0.00001	0.00001	0.00001	0.00001	0.004218	0.384155	0.611569
Japanese	HGDP00758	0.00001	0.00001	0.00001	0.00001	0.00001	0.00001	0.378838	0.621102
Melanesians	HGDP00491	0.00001	0.00001	0.00001	0.00001	0.00001	0.767337	0.232603	0.00001
Melanesians	HGDP00655	0.00001	0.00001	0.00001	0.00001	0.00001	0.764076	0.235864	0.00001
Melanesians	HGDP00656	0.00001	0.00001	0.00001	0.00001	0.00001	0.761723	0.238217	0.00001
Melanesians	HGDP00661	0.00001	0.00001	0.00001	0.00001	0.00001	0.764482	0.235458	0.00001
Melanesians	HGDP00662	0.00001	0.00001	0.00001	0.00001	0.00001	0.741695	0.258245	0.00001
Melanesians	HGDP00664	0.00001	0.00001	0.00001	0.000011	0.00001	0.765556	0.234383	0.00001
Melanesians	HGDP00787	0.00001	0.000011	0.00001	0.00001	0.00001	0.787364	0.212575	0.00001
Melanesians	HGDP00788	0.00001	0.00001	0.00001	0.00001	0.00001	0.785382	0.214558	0.00001
Melanesians	HGDP00979	0.00001	0.00001	0.00001	0.00001	0.00001	0.768425	0.231515	0.00001
Melanesians	HGDP01027	0.00001	0.00001	0.00001	0.00001	0.00001	0.763654	0.236286	0.00001
Papuans	HGDP00540	0.00001	0.00001	0.00001	0.00001	0.00001	0.99993	0.00001	0.00001
Papuans	HGDP00541	0.00001	0.00001	0.00001	0.00001	0.00001	0.99993	0.00001	0.00001
Papuans	HGDP00542	0.000018	0.00001	0.00001	0.00001	0.00001	0.999922	0.00001	0.00001
Papuans	HGDP00543	0.00001	0.00001	0.00001	0.00001	0.00001	0.99993	0.00001	0.00001
Papuans	HGDP00544	0.00001	0.00001	0.00001	0.00001	0.00001	0.792685	0.207255	0.00001
Papuans	HGDP00545	0.00001	0.00001	0.00001	0.00001	0.00001	0.99993	0.00001	0.00001
Papuans	HGDP00546	0.00001	0.00001	0.00001	0.00001	0.00001	0.99993	0.00001	0.00001
Papuans	HGDP00547	0.00001	0.00001	0.00001	0.00001	0.00001	0.99993	0.00001	0.00001
Papuans	HGDP00548	0.00001	0.00001	0.00001	0.00001	0.00001	0.99993	0.00001	0.00001
Papuans	HGDP00549	0.00001	0.00001	0.00001	0.00001	0.00001	0.99993	0.00001	0.00001
Papuans	HGDP00550	0.000018	0.00001	0.00001	0.00001	0.00001	0.999922	0.00001	0.00001
Papuans	HGDP00551	0.00001	0.00001	0.00001	0.00001	0.00001	0.99993	0.00001	0.00001
Papuans	HGDP00552	0.00001	0.00001	0.00001	0.00001	0.00001	0.99993	0.00001	0.00001
Papuans	HGDP00553	0.000014	0.00001	0.00001	0.00001	0.00001	0.999926	0.00001	0.00001
Papuans	HGDP00554	0.00001	0.00001	0.00001	0.00001	0.00001	0.99993	0.00001	0.00001
Papuans	HGDP00555	0.00001	0.00001	0.00001	0.00001	0.00001	0.99993	0.00001	0.00001
Papuans	HGDP00556	0.000017	0.00001	0.00001	0.00001	0.00001	0.999923	0.00001	0.00001

Table S5. Formal test of admixture in the form of ƒ3 (Iran_Neolithic,X;Y); where X represent Arabian and Middle Eastern populations and Y represent Iranian and Parsi populations

Source 1	Source 2	Target	ƒ3 value	SE	Z score	SNPs
Iran_Neolithic (I1290)	Mozabites	Iranians	-0.00664	0.000628	-10.561	208373
Iran_Neolithic (I1290)	Bedouins	Iranians	-0.00585	0.000531	-11.022	208373
Iran_Neolithic (I1290)	Palestinians	Iranians	-0.00454	0.000539	-8.426	208373
Iran_Neolithic (I1290)	Jordanians	Iranians	-0.00409	0.000566	-7.219	208373
Iran_Neolithic (I1290)	Syrians	Iranians	-0.00375	0.000593	-6.321	208373
Iran_Neolithic (I1290)	Druze	Iranians	-0.00435	0.000548	-7.925	208373
Iran_Neolithic (I1290)	Iraqis	Iranians	-0.00123	0.000669	-1.836	208373
Iran_Neolithic (I1290)	Turks	Iranians	-0.00247	0.000573	-4.313	208373
Iran_Neolithic (I1290)	Kurds	Iranians	0	0.000656	0	208373
Iran_Neolithic (I1290)	Mozabites	Parsi_Pakistan	0.005291	0.000697	7.591	208373
Iran_Neolithic (I1290)	Bedouins	Parsi_Pakistan	0.005776	0.000592	9.749	208373
Iran_Neolithic (I1290)	Palestinians	Parsi_Pakistan	0.00652	0.000624	10.451	208373
Iran_Neolithic (I1290)	Jordanians	Parsi_Pakistan	0.00698	0.000654	10.68	208373
Iran_Neolithic (I1290)	Syrians	Parsi_Pakistan	0.007219	0.000674	10.717	208373
Iran_Neolithic (I1290)	Druze	Parsi_Pakistan	0.006671	0.000642	10.388	208373
Iran_Neolithic (I1290)	Iraqis	Parsi_Pakistan	0.009533	0.000731	13.043	208373
Iran_Neolithic (I1290)	Turks	Parsi_Pakistan	0.007783	0.000597	13.037	208373
Iran_Neolithic (I1290)	Kurds	Parsi_Pakistan	0.010431	0.000732	14.255	208373
Iran_Neolithic (I1290)	Mozabites	Parsi_India	0.006813	0.000753	9.046	208373
Iran_Neolithic (I1290)	Bedouins	Parsi_India	0.00742	0.000653	11.367	208373
Iran_Neolithic (I1290)	Palestinians	Parsi_India	0.008332	0.000669	12.448	208373
Iran_Neolithic (I1290)	Jordanians	Parsi_India	0.008871	0.000728	12.179	208373
Iran_Neolithic (I1290)	Syrians	Parsi_India	0.008648	0.000715	12.101	208373
Iran_Neolithic (I1290)	Druze	Parsi_India	0.008541	0.000698	12.243	208373
Iran_Neolithic (I1290)	Iraqis	Parsi_India	0.010926	0.000763	14.315	208373
Iran_Neolithic (I1290)	Turks	Parsi_India	0.009246	0.00066	14.019	208373
Iran_Neolithic (I1290)	Kurds	Parsi_India	0.012033	0.000821	14.656	208373

Table S6. Computed *D* Statistic results showing geneflow between Neolithic Iranian and other modern populations

Gp1	Gp2	Gp3	*D* value	Z score	SNP
Iran_Neolithic (I1290)	Bedouins	Iranians	-0.0313	-19.28	208373
Iran_Neolithic (I1290)	Palestinians	Iranians	-0.0238	-14.987	208373
Iran_Neolithic (I1290)	Jordanians	Iranians	-0.0429	-24.983	208373
Iran_Neolithic (I1290)	Syrians	Iranians	-0.0112	-6.541	208373
Iran_Neolithic (I1290)	Druze	Iranians	-0.0052	-3.397	208373
Iran_Neolithic (I1290)	Iraqis	Iranians	-0.0065	-3.416	208373
Iran_Neolithic (I1290)	Turks	Iranians	0.0051	3.15	208373
Iran_Neolithic (I1290)	Kurds	Iranians	0.0135	7.34	208373
Iran_Neolithic (I1290)	Azeris	Iranians	0.0049	3.793	208373
Iran_Neolithic (I1290)	French	Iranians	-0.0038	-2.442	208373
Iran_Neolithic (I1290)	Sardinians	Iranians	-0.0104	-6.16	208373
Iran_Neolithic (I1290)	North_Italians	Iranians	-0.0019	-1.093	208373
Iran_Neolithic (I1290)	Tuscans	Iranians	-0.003	-1.748	208373
Iran_Neolithic (I1290)	Russians	Iranians	-0.0036	-2.229	208373
Iran_Neolithic (I1290)	Estonians	Iranians	-0.0047	-2.869	208373
Iran_Neolithic (I1290)	Armenians	Iranians	0.0073	5.602	208373
Iran_Neolithic (I1290)	Georgians	Iranians	0.0108	7.786	208373
Iran_Neolithic (I1290)	Abkhasians	Iranians	0.0119	7.002	208373
Iran_Neolithic (I1290)	Balkars	Iranians	0.0037	2.395	208373
Iran_Neolithic (I1290)	Chechens	Iranians	0.0092	5.815	208373
Iran_Neolithic (I1290)	Lezgins	Iranians	0.011	6.536	208373
Iran_Neolithic (I1290)	Kumyks	Iranians	0.004	2.519	208373
Iran_Neolithic (I1290)	Nogais	Iranians	-0.0038	-2.277	208373
Iran_Neolithic (I1290)	Turkmens	Iranians	-0.0044	-2.322	208373
Iran_Neolithic (I1290)	Tajiks	Iranians	0.0013	0.815	208373
Iran_Neolithic (I1290)	Uzbeks	Iranians	-0.0141	-8.384	208373
Iran_Neolithic (I1290)	Kyrgyzians	Iranians	-0.028	-12.069	208366
Iran_Neolithic (I1290)	Hazara	Iranians	-0.0161	-8.615	208373
Iran_Neolithic (I1290)	Kalash	Iranians	0.0059	2.862	208373
Iran_Neolithic (I1290)	Pathan	Iranians	0.0038	2.409	208373
Iran_Neolithic (I1290)	Burusho	Iranians	-0.0032	-1.833	208373
Iran_Neolithic (I1290)	Balochi	Iranians	-0.0041	-2.686	208373
Iran_Neolithic (I1290)	Brahui	Iranians	0.0079	4.742	208373
Iran_Neolithic (I1290)	Makrani	Iranians	-0.0169	-10.003	208373
Iran_Neolithic (I1290)	Sindhi	Iranians	-0.0002	-0.141	208373
Iran_Neolithic (I1290)	Gujaratis	Iranians	-0.008	-5.075	208373
Iran_Neolithic (I1290)	Brahmins_UP	Iranians	-0.0067	-3.581	208373
Iran_Neolithic (I1290)	Meena	Iranians	-0.0053	-1.384	208373
Iran_Neolithic (I1290)	Meghawal	Iranians	-0.0163	-4.363	208373
Iran_Neolithic (I1290)	Parsi_India	Iranians	0.005	3.2	208373
Iran_Neolithic (I1290)	Parsi_Pakistan	Iranians	0.0058	4.083	208373
Iran_Neolithic (I1290)	Kshatriya	Iranians	-0.0089	-4.704	208373
Iran_Neolithic (I1290)	Harijan	Iranians	-0.0218	-10.608	208373
Iran_Neolithic (I1290)	Dharkars	Iranians	-0.0157	-8.115	208373
Iran_Neolithic (I1290)	Dusadh	Iranians	-0.0182	-8.986	208373
Iran_Neolithic (I1290)	Kanjars	Iranians	-0.0155	-7.921	208373
Iran_Neolithic (I1290)	Kol	Iranians	-0.0189	-10.538	208373
Iran_Neolithic (I1290)	Kurmi	Iranians	-0.0156	-3.927	208373
Iran_Neolithic (I1290)	UP_Low_Caste	Iranians	-0.0171	-7.483	208373
Iran_Neolithic (I1290)	Tharus	Iranians	-0.0147	-5.391	208373
Iran_Neolithic (I1290)	Bhunjia	Iranians	-0.0458	-11.631	208373
Iran_Neolithic (I1290)	Dhurwa	Iranians	-0.0464	-11.944	208373
Iran_Neolithic (I1290)	Gond	Iranians	-0.0259	-10.64	208373
Iran_Neolithic (I1290)	Chenchus	Iranians	-0.0201	-7.905	208373
Iran_Neolithic (I1290)	Velmas	Iranians	-0.0094	-4.993	208373
Iran_Neolithic (I1290)	Halakipikki	Iranians	-0.0234	-9.426	208373
Iran_Neolithic (I1290)	North_Kannadi	Iranians	-0.0243	-11.209	208373
Iran_Neolithic (I1290)	Malayan	Iranians	-0.0359	-11.597	208367
Iran_Neolithic (I1290)	Paniya	Iranians	-0.0367	-13.606	208373
Iran_Neolithic (I1290)	Sakilli	Iranians	-0.0233	-9.184	208373
Iran_Neolithic (I1290)	Kurumba	Iranians	-0.0143	-5.771	208373

Iran_Neolithic (I1290)	TN_Low_Caste	Iranians	-0.0173	-5.788	208373
Iran_Neolithic (I1290)	Piramalai_Kallars	Iranians	-0.0176	-8.485	208373
Iran_Neolithic (I1290)	Pulliyar	Iranians	-0.0319	-11.412	208373
Iran_Neolithic (I1290)	Asur	Iranians	-0.0409	-12.908	208373
Iran_Neolithic (I1290)	Ho	Iranians	-0.0444	-17.507	208373
Iran_Neolithic (I1290)	Mawasi	Iranians	-0.0397	-10.613	208373
Iran_Neolithic (I1290)	Santhal	Iranians	-0.0391	-10.128	208373
Iran_Neolithic (I1290)	Bonda	Iranians	-0.0484	-16.966	208373
Iran_Neolithic (I1290)	Gadaba	Iranians	-0.0496	-12.505	208373
Iran_Neolithic (I1290)	Juang	Iranians	-0.0474	-15.336	208373
Iran_Neolithic (I1290)	Kharia	Iranians	-0.0445	-13.781	208373
Iran_Neolithic (I1290)	Savara	Iranians	-0.0461	-14.937	208373
Iran_Neolithic (I1290)	Khasi	Iranians	-0.0434	-15.516	208373
Iran_Neolithic (I1290)	Garo	Iranians	-0.0478	-16.381	208373
Iran_Neolithic (I1290)	Naga	Iranians	-0.0519	-17.167	208373
Iran_Neolithic (I1290)	Burmese	Iranians	-0.0454	-18.837	208373
Iran_Neolithic (I1290)	Cambodians	Iranians	-0.0473	-18.33	208373
Iran_Neolithic (I1290)*	Parsis	Iranians	0.0012	0.698	62212
Iran_Neolithic (I1290,I1945)	Parsi_India	Iranians	0.0038	1.628	27472
Iran_Neolithic (I1290,I1945)	Parsi_Pakistan	Iranians	0.0037	1.785	27472
Iran_Late_Neolithic (I1671)	Parsi_India	Iranians	0.0084	5.597	128901
Iran_Late_Neolithic (I1671)	Parsi_Pakistan	Iranians	0.0074	5.022	128901
Iran_HotuIIb (1)*	Parsis	Iranians	0.0071	1.862	10340
Iran_Late_Neolithic (I1671)*	Parsis	Iranians	0.0073	3.448	36424
Iran_Chalcolithic (5)*	Parsis	Iranians	-1.00E-04	-0.106	72217
Levant_Neolithic (15)*	Parsis	Iranians	-0.0021	-1.42	60847

D= (Gp1,Yoruba;Gp2,Gp3)

D*= (Gp1,Khomani;Gp2,Gp3)

Table S7. The MALDER test showing the single admixture event of Parsis

Target	Reference 1	Reference 2	Amplitude	Amplitude_SD	Zscore	Time	Time_SD
Parsis	Papuans	Sindhi	2.92E-05	2.73E-06	10.7056	54.5601	8.56415
Parsis	Papuans	Nogais	6.46E-05	6.05E-06	10.6719	54.5601	8.56415
Parsis	Papuans	Tajiks	4.97E-05	4.75E-06	10.459	54.5601	8.56415
Parsis	Iranians	Pathan	1.55E-05	1.54E-06	10.0968	54.5601	8.56415
Parsis	Brahui	Sindhi	4.75E-06	4.71E-07	10.085	54.5601	8.56415
Parsis	Kol	Tajiks	3.07E-05	3.04E-06	10.0742	54.5601	8.56415
Parsis	Papuans	Palestinians	1.09E-04	1.08E-05	10.0268	54.5601	8.56415
Parsis	Azeris	Pathan	1.38E-05	1.38E-06	10.0076	54.5601	8.56415
Parsis	Papuans	Mozabites	1.12E-04	1.12E-05	9.99697	54.5601	8.56415
Parsis	Chamar	Mozabites	9.17E-05	9.23E-06	9.94499	54.5601	8.56415
Parsis	Gujaratis	Nogais	2.57E-05	2.59E-06	9.94165	54.5601	8.56415
Parsis	Chamar	Tajiks	3.85E-05	3.87E-06	9.92904	54.5601	8.56415
Parsis	Papuans	Brahui	5.23E-05	5.28E-06	9.8956	54.5601	8.56415
Parsis	Papuans	French	1.02E-04	1.04E-05	9.89379	54.5601	8.56415
Parsis	Papuans	Bedouins	1.15E-04	1.16E-05	9.85462	54.5601	8.56415
Parsis	Balochi	Chamar	4.19E-05	4.25E-06	9.83822	54.5601	8.56415
Parsis	Papuans	Azeris	9.44E-05	9.65E-06	9.78823	54.5601	8.56415
Parsis	Kol	Mozabites	8.27E-05	8.45E-06	9.78634	54.5601	8.56415
Parsis	Armenians	Papuans	1.09E-04	1.11E-05	9.78495	54.5601	8.56415
Parsis	Papuans	Russians	9.06E-05	9.26E-06	9.77966	54.5601	8.56415
Parsis	Kol	Nogais	4.25E-05	4.35E-06	9.76967	54.5601	8.56415
Parsis	Gujaratis	Tajiks	1.89E-05	1.93E-06	9.76721	54.5601	8.56415
Parsis	Miaozu	Mozabites	1.13E-04	1.16E-05	9.7636	54.5601	8.56415
Parsis	Iranians	Chamar	7.67E-05	7.86E-06	9.7583	54.5601	8.56415
Parsis	Melanesians	Nogais	6.47E-05	6.63E-06	9.7569	54.5601	8.56415
Parsis	Papuans	Druze	1.19E-04	1.22E-05	9.75641	54.5601	8.56415
Parsis	Kol	Balochi	3.12E-05	3.20E-06	9.7487	54.5601	8.56415
Parsis	Pathan	Palestinians	2.59E-05	2.66E-06	9.73726	54.5601	8.56415
Parsis	Papuans	Georgians	1.05E-04	1.09E-05	9.70695	54.5601	8.56415
Parsis	Brahui	Chamar	4.19E-05	4.32E-06	9.70186	54.5601	8.56415
Parsis	Azeris	Chamar	7.31E-05	7.54E-06	9.69854	54.5601	8.56415
Parsis	Kol	Brahui	3.21E-05	3.31E-06	9.6897	54.5601	8.56415
Parsis	Papuans	Estonians	9.08E-05	9.37E-06	9.68825	54.5601	8.56415
Parsis	Gujaratis	Iranians	4.42E-05	4.57E-06	9.6803	54.5601	8.56415
Parsis	Nogais	Velmas	3.54E-05	3.66E-06	9.67333	54.5601	8.56415
Parsis	Chamar	Palestinians	9.26E-05	9.58E-06	9.6655	54.5601	8.56415
Parsis	Iranians	Kol	6.34E-05	6.58E-06	9.63904	54.5601	8.56415
Parsis	Georgians	Chamar	8.80E-05	9.15E-06	9.62075	54.5601	8.56415
Parsis	Chamar	Nogais	5.17E-05	5.37E-06	9.61893	54.5601	8.56415
Parsis	Melanesians	Palestinians	1.02E-04	1.07E-05	9.61272	54.5601	8.56415
Parsis	Iranians	Papuans	9.33E-05	9.71E-06	9.60544	54.5601	8.56415
Parsis	Bedouins	Chamar	9.89E-05	1.03E-05	9.60273	54.5601	8.56415
Parsis	Gujaratis	Palestinians	5.98E-05	6.23E-06	9.60026	54.5601	8.56415
Parsis	Gujaratis	Azeris	4.23E-05	4.41E-06	9.59357	54.5601	8.56415
Parsis	Papuans	Pathan	4.02E-05	4.20E-06	9.5893	54.5601	8.56415
Parsis	Kol	Palestinians	8.11E-05	8.46E-06	9.58475	54.5601	8.56415
Parsis	Kol	Druze	8.81E-05	9.20E-06	9.58001	54.5601	8.56415
Parsis	Gujaratis	Mozabites	6.01E-05	6.27E-06	9.57595	54.5601	8.56415
Parsis	Tajiks	Velmas	2.58E-05	2.69E-06	9.5682	54.5601	8.56415
Parsis	Armenians	Kol	7.88E-05	8.24E-06	9.56658	54.5601	8.56415
Parsis	Pathan	Mozabites	3.02E-05	3.16E-06	9.55132	54.5601	8.56415
Parsis	Makrani	Mozabites	2.46E-05	2.58E-06	9.54803	54.5601	8.56415
Parsis	Papuans	Bantus	6.75E-05	7.07E-06	9.54584	54.5601	8.56415
Parsis	Kol	Bedouins	8.85E-05	9.29E-06	9.5345	54.5601	8.56415
Parsis	Iranians	Velmas	5.53E-05	5.80E-06	9.53259	54.5601	8.56415
Parsis	Kol	Georgians	7.72E-05	8.10E-06	9.5318	54.5601	8.56415
Parsis	Burmese	Mozabites	1.11E-04	1.16E-05	9.52432	54.5601	8.56415
Parsis	Dai	Mozabites	1.25E-04	1.31E-05	9.52214	54.5601	8.56415
Parsis	Han	Mozabites	1.19E-04	1.25E-05	9.51815	54.5601	8.56415
Parsis	Armenians	Chamar	9.10E-05	9.57E-06	9.5096	54.5601	8.56415
Parsis	Chamar	Druze	1.01E-04	1.06E-05	9.50372	54.5601	8.56415
Parsis	Azeris	Kol	6.27E-05	6.60E-06	9.50116	54.5601	8.56415
Parsis	French	Chamar	9.30E-05	9.79E-06	9.49765	54.5601	8.56415
Parsis	Mozabites	She	1.22E-04	1.28E-05	9.49027	54.5601	8.56415
Parsis	Miaozu	Nogais	6.96E-05	7.34E-06	9.4787	54.5601	8.56415
Parsis	Papuans	Mandenkas	6.37E-05	6.73E-06	9.47788	54.5601	8.56415
Parsis	Gujaratis	Bedouins	6.55E-05	6.92E-06	9.46749	54.5601	8.56415
Parsis	Papuans	Sardinians	1.24E-04	1.31E-05	9.45705	54.5601	8.56415
Parsis	Chamar	Sindhi	2.19E-05	2.31E-06	9.45317	54.5601	8.56415
Parsis	Mongola	Mozabites	9.87E-05	1.04E-05	9.44975	54.5601	8.56415
Parsis	Estonians	Chamar	8.10E-05	8.58E-06	9.44943	54.5601	8.56415
Parsis	Azeris	Velmas	5.34E-05	5.65E-06	9.44934	54.5601	8.56415
Parsis	Russians	Chamar	7.69E-05	8.14E-06	9.43675	54.5601	8.56415
Parsis	Melanesians	Mozabites	1.01E-04	1.08E-05	9.43144	54.5601	8.56415
Parsis	Iranians	Sindhi	2.20E-05	2.33E-06	9.43045	54.5601	8.56415
Parsis	Bedouins	Melanesians	1.09E-04	1.15E-05	9.43002	54.5601	8.56415
Parsis	Druze	Melanesians	1.15E-04	1.22E-05	9.42475	54.5601	8.56415
Parsis	Japanese	Mozabites	1.19E-04	1.26E-05	9.42401	54.5601	8.56415
Parsis	Gujaratis	Armenians	5.65E-05	6.00E-06	9.4211	54.5601	8.56415
Parsis	Kol	French	8.05E-05	8.54E-06	9.4209	54.5601	8.56415
Parsis	Armenians	Melanesians	1.06E-04	1.13E-05	9.41572	54.5601	8.56415
Parsis	French	Melanesians	9.84E-05	1.05E-05	9.41457	54.5601	8.56415
Parsis	Mozabites	Tujia	1.19E-04	1.26E-05	9.41435	54.5601	8.56415
Parsis	Gujaratis	Druze	6.49E-05	6.90E-06	9.41084	54.5601	8.56415
Parsis	Mozabites	Tu	1.04E-04	1.10E-05	9.40089	54.5601	8.56415
Parsis	Burmese	Nogais	6.96E-05	7.41E-06	9.39201	54.5601	8.56415
Parsis	Sardinians	Chamar	1.13E-04	1.21E-05	9.38908	54.5601	8.56415
Parsis	Kol	Russians	6.66E-05	7.09E-06	9.38516	54.5601	8.56415
Parsis	Palestinians	Velmas	7.14E-05	7.62E-06	9.37435	54.5601	8.56415
Parsis	Kol	Estonians	7.02E-05	7.49E-06	9.37019	54.5601	8.56415
Parsis	Burmese	Tajiks	5.05E-05	5.39E-06	9.36689	54.5601	8.56415
Parsis	Palestinians	Sindhi	3.40E-05	3.63E-06	9.36415	54.5601	8.56415
Parsis	Mozabites	Velmas	7.50E-05	8.01E-06	9.36303	54.5601	8.56415

Parsis	Papuans	Balochi	5.41E-05	5.78E-06	9.36079	54.5601	8.56415
Parsis	Dai	Tajiks	5.92E-05	6.33E-06	9.35925	54.5601	8.56415
Parsis	Gujaratis	Georgians	5.46E-05	5.85E-06	9.34888	54.5601	8.56415
Parsis	Papuans	Makrani	5.30E-05	5.67E-06	9.34746	54.5601	8.56415
Parsis	Mozabites	Yizu	1.21E-04	1.29E-05	9.33341	54.5601	8.56415
Parsis	Pathan	Chamar	2.95E-05	3.16E-06	9.32688	54.5601	8.56415
Parsis	Georgians	Melanesians	1.03E-04	1.10E-05	9.31761	54.5601	8.56415
Parsis	Cambodians	Mozabites	1.12E-04	1.21E-05	9.30989	54.5601	8.56415
Parsis	Bedouins	Pathan	3.04E-05	3.26E-06	9.30521	54.5601	8.56415
Parsis	Bedouins	Sindhi	3.84E-05	4.13E-06	9.30096	54.5601	8.56415
Parsis	Bedouins	Miaozu	1.22E-04	1.31E-05	9.29777	54.5601	8.56415
Parsis	Iranians	Melanesians	8.79E-05	9.46E-06	9.29586	54.5601	8.56415
Parsis	Mozabites	Sindhi	3.94E-05	4.24E-06	9.29056	54.5601	8.56415
Parsis	Cambodians	Nogais	7.13E-05	7.69E-06	9.28209	54.5601	8.56415
Parsis	Nogais	Yizu	7.37E-05	7.94E-06	9.28097	54.5601	8.56415
Parsis	Nogais	Tujia	7.22E-05	7.78E-06	9.27859	54.5601	8.56415
Parsis	Bedouins	Dai	1.32E-04	1.42E-05	9.27456	54.5601	8.56415
Parsis	Balochi	Mozabites	2.82E-05	3.04E-06	9.26991	54.5601	8.56415
Parsis	Miaozu	Palestinians	1.14E-04	1.23E-05	9.26905	54.5601	8.56415
Parsis	Armenians	Velmas	6.84E-05	7.38E-06	9.26801	54.5601	8.56415
Parsis	Han	Nogais	7.41E-05	8.00E-06	9.2668	54.5601	8.56415
Parsis	Bedouins	Velmas	7.89E-05	8.52E-06	9.2644	54.5601	8.56415
Parsis	Mongola	Nogais	5.88E-05	6.35E-06	9.26111	54.5601	8.56415
Parsis	Nogais	Tu	6.26E-05	6.76E-06	9.25839	54.5601	8.56415
Parsis	Dai	Nogais	7.99E-05	8.63E-06	9.25682	54.5601	8.56415
Parsis	Burusho	Palestinians	3.55E-05	3.84E-06	9.25538	54.5601	8.56415
Parsis	Brahui	Velmas	2.77E-05	2.99E-06	9.25487	54.5601	8.56415
Parsis	Gujaratis	French	5.95E-05	6.43E-06	9.25157	54.5601	8.56415
Parsis	Gujaratis	Balochi	1.97E-05	2.13E-06	9.24842	54.5601	8.56415
Parsis	Mongola	Tajiks	4.30E-05	4.66E-06	9.24295	54.5601	8.56415
Parsis	Makrani	Palestinians	1.73E-05	1.87E-06	9.24291	54.5601	8.56415
Parsis	Azeris	Dai	1.05E-04	1.14E-05	9.23499	54.5601	8.56415
Parsis	Gujaratis	Brahui	1.93E-05	2.09E-06	9.23477	54.5601	8.56415
Parsis	Chamar	Makrani	4.42E-05	4.79E-06	9.23409	54.5601	8.56415
Parsis	Azeris	Miaozu	9.60E-05	1.04E-05	9.23212	54.5601	8.56415
Parsis	Gujaratis	Estonians	4.97E-05	5.39E-06	9.22868	54.5601	8.56415
Parsis	Azeris	Melanesians	8.80E-05	9.54E-06	9.22599	54.5601	8.56415
Parsis	Kol	Sardinians	1.00E-04	1.09E-05	9.2243	54.5601	8.56415
Parsis	Bedouins	She	1.30E-04	1.41E-05	9.21876	54.5601	8.56415
Parsis	Han	Bedouins	1.28E-04	1.38E-05	9.21836	54.5601	8.56415
Parsis	Bedouins	Mongola	1.07E-04	1.17E-05	9.21466	54.5601	8.56415
Parsis	Han	Tajiks	5.56E-05	6.04E-06	9.20748	54.5601	8.56415
Parsis	Azeris	Burmese	9.15E-05	9.94E-06	9.20739	54.5601	8.56415
Parsis	Papuans	Mbuti_Pygmies	6.62E-05	7.19E-06	9.206	54.5601	8.56415
Parsis	Gujaratis	Pathan	1.07E-05	1.16E-06	9.20431	54.5601	8.56415
Parsis	Brahui	She	6.83E-05	7.42E-06	9.1992	54.5601	8.56415
Parsis	Nogais	She	7.38E-05	8.03E-06	9.19381	54.5601	8.56415
Parsis	Russians	Melanesians	8.59E-05	9.34E-06	9.19216	54.5601	8.56415
Parsis	Burmese	Palestinians	1.11E-04	1.21E-05	9.19206	54.5601	8.56415
Parsis	Palestinians	She	1.22E-04	1.33E-05	9.19177	54.5601	8.56415
Parsis	Iranians	Miaozu	9.80E-05	1.07E-05	9.18838	54.5601	8.56415
Parsis	Armenians	Burusho	3.37E-05	3.66E-06	9.18632	54.5601	8.56415
Parsis	Armenians	Sindhi	3.11E-05	3.38E-06	9.18568	54.5601	8.56415
Parsis	Armenians	Pathan	2.35E-05	2.56E-06	9.17793	54.5601	8.56415
Parsis	Bedouins	Tujia	1.26E-04	1.38E-05	9.17685	54.5601	8.56415
Parsis	She	Tajiks	5.66E-05	6.17E-06	9.17243	54.5601	8.56415
Parsis	Burmese	Bedouins	1.19E-04	1.30E-05	9.17124	54.5601	8.56415
Parsis	Estonians	Melanesians	8.73E-05	9.52E-06	9.16752	54.5601	8.56415
Parsis	Han	Azeris	9.98E-05	1.09E-05	9.16464	54.5601	8.56415
Parsis	Azeris	Sindhi	2.15E-05	2.35E-06	9.16458	54.5601	8.56415
Parsis	Azeris	She	1.02E-04	1.11E-05	9.1601	54.5601	8.56415
Parsis	Bedouins	Japanese	1.25E-04	1.36E-05	9.15707	54.5601	8.56415
Parsis	French	Dai	1.22E-04	1.33E-05	9.15393	54.5601	8.56415
Parsis	Burusho	Mozabites	4.29E-05	4.68E-06	9.15332	54.5601	8.56415
Parsis	Azeris	Mongola	8.06E-05	8.81E-06	9.15145	54.5601	8.56415
Parsis	Dai	Palestinians	1.23E-04	1.34E-05	9.15038	54.5601	8.56415
Parsis	Druze	Velmas	7.71E-05	8.43E-06	9.15036	54.5601	8.56415
Parsis	Han	Iranians	1.03E-04	1.13E-05	9.1388	54.5601	8.56415
Parsis	Iranians	Dai	1.07E-04	1.17E-05	9.13859	54.5601	8.56415
Parsis	Bedouins	Cambodians	1.21E-04	1.32E-05	9.1366	54.5601	8.56415
Parsis	Bedouins	Yizu	1.26E-04	1.38E-05	9.13576	54.5601	8.56415
Parsis	French	Miaozu	1.11E-04	1.22E-05	9.13563	54.5601	8.56415
Parsis	Miaozu	Tajiks	5.26E-05	5.76E-06	9.13371	54.5601	8.56415
Parsis	Han	Palestinians	1.19E-04	1.31E-05	9.12572	54.5601	8.56415
Parsis	Japanese	Nogais	7.20E-05	7.89E-06	9.12279	54.5601	8.56415
Parsis	Iranians	She	1.07E-04	1.17E-05	9.12095	54.5601	8.56415
Parsis	Azeris	Burusho	2.40E-05	2.63E-06	9.12008	54.5601	8.56415
Parsis	Palestinians	Tujia	1.18E-04	1.30E-05	9.11362	54.5601	8.56415
Parsis	Brahui	Dai	6.98E-05	7.66E-06	9.11235	54.5601	8.56415
Parsis	Iranians	Makrani	8.03E-06	8.81E-07	9.107	54.5601	8.56415
Parsis	Druze	Miaozu	1.25E-04	1.37E-05	9.1022	54.5601	8.56415
Parsis	Iranians	Tujia	1.02E-04	1.13E-05	9.0965	54.5601	8.56415
Parsis	Gujaratis	Russians	4.65E-05	5.11E-06	9.09643	54.5601	8.56415
Parsis	Burmese	Estonians	9.68E-05	1.06E-05	9.09592	54.5601	8.56415
Parsis	Han	French	1.17E-04	1.29E-05	9.09563	54.5601	8.56415
Parsis	Palestinians	Yizu	1.18E-04	1.30E-05	9.09201	54.5601	8.56415
Parsis	Azeris	Makrani	1.00E-05	1.10E-06	9.09161	54.5601	8.56415
Parsis	Burmese	French	1.10E-04	1.21E-05	9.08902	54.5601	8.56415
Parsis	Tajiks	Yizu	5.59E-05	6.15E-06	9.08867	54.5601	8.56415
Parsis	Burusho	Chamar	2.04E-05	2.25E-06	9.08654	54.5601	8.56415
Parsis	Georgians	Dai	1.23E-04	1.35E-05	9.08483	54.5601	8.56415
Parsis	Balochi	Dai	6.93E-05	7.63E-06	9.08455	54.5601	8.56415
Parsis	Azeris	Balochi	1.06E-05	1.17E-06	9.08384	54.5601	8.56415
Parsis	Kol	Pathan	2.25E-05	2.48E-06	9.0827	54.5601	8.56415
Parsis	Bedouins	Tu	1.09E-04	1.20E-05	9.07939	54.5601	8.56415

Parsis	Iranians	Burusho	2.27E-05	2.50E-06	9.07743	54.5601	8.56415
Parsis	Papuans	Yorubas	6.38E-05	7.03E-06	9.07722	54.5601	8.56415
Parsis	Makrani	Sindhi	6.16E-06	6.79E-07	9.07364	54.5601	8.56415
Parsis	Azeris	Tujia	9.96E-05	1.10E-05	9.07207	54.5601	8.56415
Parsis	Mongola	Palestinians	1.00E-04	1.11E-05	9.06907	54.5601	8.56415
Parsis	French	She	1.18E-04	1.30E-05	9.06143	54.5601	8.56415
Parsis	Tajiks	Tu	4.53E-05	5.00E-06	9.05915	54.5601	8.56415
Parsis	Sardinians	Miaozu	1.33E-04	1.46E-05	9.05826	54.5601	8.56415
Parsis	Burmese	Druze	1.23E-04	1.35E-05	9.05565	54.5601	8.56415
Parsis	French	Pathan	2.51E-05	2.77E-06	9.05468	54.5601	8.56415
Parsis	Iranians	Yizu	1.03E-04	1.14E-05	9.0543	54.5601	8.56415
Parsis	Georgians	Miaozu	1.12E-04	1.24E-05	9.0529	54.5601	8.56415
Parsis	French	Mongola	9.70E-05	1.07E-05	9.0516	54.5601	8.56415
Parsis	Druze	Sindhi	3.68E-05	4.07E-06	9.04913	54.5601	8.56415
Parsis	Dai	Druze	1.35E-04	1.50E-05	9.04893	54.5601	8.56415
Parsis	French	Tujia	1.16E-04	1.28E-05	9.04826	54.5601	8.56415
Parsis	Han	Estonians	1.03E-04	1.14E-05	9.04676	54.5601	8.56415
Parsis	Iranians	Burmese	9.52E-05	1.05E-05	9.04623	54.5601	8.56415
Parsis	Estonians	Dai	1.08E-04	1.19E-05	9.04442	54.5601	8.56415
Parsis	Cambodians	Palestinians	1.14E-04	1.26E-05	9.04374	54.5601	8.56415
Parsis	Han	Georgians	1.18E-04	1.30E-05	9.04205	54.5601	8.56415
Parsis	Brahui	Miaozu	6.11E-05	6.76E-06	9.03998	54.5601	8.56415
Parsis	Azeris	Tu	8.50E-05	9.40E-06	9.03758	54.5601	8.56415
Parsis	Estonians	Miaozu	9.76E-05	1.08E-05	9.03728	54.5601	8.56415
Parsis	Estonians	Velmas	6.22E-05	6.88E-06	9.03694	54.5601	8.56415
Parsis	Armenians	Miaozu	1.15E-04	1.28E-05	9.03655	54.5601	8.56415
Parsis	Balochi	She	6.83E-05	7.56E-06	9.03496	54.5601	8.56415
Parsis	Japanese	Palestinians	1.17E-04	1.30E-05	9.03452	54.5601	8.56415
Parsis	Iranians	Cambodians	9.80E-05	1.08E-05	9.03304	54.5601	8.56415
Parsis	Armenians	Dai	1.25E-04	1.39E-05	9.03142	54.5601	8.56415
Parsis	Burmese	Russians	9.32E-05	1.03E-05	9.02981	54.5601	8.56415
Parsis	French	Velmas	7.12E-05	7.88E-06	9.02895	54.5601	8.56415
Parsis	Armenians	She	1.21E-04	1.35E-05	9.02272	54.5601	8.56415
Parsis	French	Japanese	1.14E-04	1.26E-05	9.02167	54.5601	8.56415
Parsis	Georgians	Cambodians	1.11E-04	1.23E-05	9.02069	54.5601	8.56415
Parsis	Sardinians	Melanesians	1.20E-04	1.33E-05	9.01831	54.5601	8.56415
Parsis	Palestinians	Tu	1.03E-04	1.14E-05	9.01763	54.5601	8.56415
Parsis	Druze	She	1.34E-04	1.48E-05	9.01724	54.5601	8.56415
Parsis	Han	Druze	1.31E-04	1.45E-05	9.01169	54.5601	8.56415
Parsis	French	Yizu	1.16E-04	1.29E-05	9.0106	54.5601	8.56415
Parsis	Georgians	She	1.18E-04	1.31E-05	9.00902	54.5601	8.56415
Parsis	Azeris	Cambodians	9.49E-05	1.05E-05	9.00806	54.5601	8.56415
Parsis	Estonians	Yizu	1.03E-04	1.15E-05	9.00697	54.5601	8.56415
Parsis	French	Tu	9.87E-05	1.10E-05	9.00326	54.5601	8.56415
Parsis	She	Sindhi	4.39E-05	4.88E-06	8.99975	54.5601	8.56415
Parsis	Estonians	Tujia	1.02E-04	1.13E-05	8.99921	54.5601	8.56415
Parsis	Balochi	Miaozu	6.10E-05	6.78E-06	8.99831	54.5601	8.56415
Parsis	Iranians	Mongola	8.59E-05	9.55E-06	8.99828	54.5601	8.56415
Parsis	Georgians	Yizu	1.17E-04	1.30E-05	8.99497	54.5601	8.56415
Parsis	Iranians	Japanese	1.00E-04	1.11E-05	8.99483	54.5601	8.56415
Parsis	Estonians	She	1.04E-04	1.16E-05	8.993	54.5601	8.56415
Parsis	Georgians	Pathan	2.17E-05	2.42E-06	8.99125	54.5601	8.56415
Parsis	Armenians	Burmese	1.13E-04	1.25E-05	8.99092	54.5601	8.56415
Parsis	Han	Russians	9.93E-05	1.10E-05	8.99046	54.5601	8.56415
Parsis	Estonians	Mongola	8.42E-05	9.37E-06	8.9897	54.5601	8.56415
Parsis	Mbuti_Pygmies	Mozabites	3.12E-05	3.47E-06	8.9892	54.5601	8.56415
Parsis	Sardinians	Dai	1.43E-04	1.60E-05	8.98773	54.5601	8.56415
Parsis	Burmese	Georgians	1.09E-04	1.21E-05	8.98594	54.5601	8.56415
Parsis	Russians	Dai	1.05E-04	1.17E-05	8.98533	54.5601	8.56415
Parsis	Azeris	Japanese	9.65E-05	1.07E-05	8.98449	54.5601	8.56415
Parsis	Cambodians	Druze	1.25E-04	1.39E-05	8.98341	54.5601	8.56415
Parsis	Han	Sardinians	1.38E-04	1.54E-05	8.98123	54.5601	8.56415
Parsis	Pathan	Druze	2.79E-05	3.11E-06	8.98069	54.5601	8.56415
Parsis	Han	Armenians	1.20E-04	1.34E-05	8.98064	54.5601	8.56415
Parsis	Japanese	Tajiks	5.36E-05	5.97E-06	8.97837	54.5601	8.56415
Parsis	Han	Brahui	6.60E-05	7.35E-06	8.97579	54.5601	8.56415
Parsis	Georgians	Mongola	9.85E-05	1.10E-05	8.97185	54.5601	8.56415
Parsis	Georgians	Sindhi	2.90E-05	3.24E-06	8.96919	54.5601	8.56415
Parsis	Dai	Sindhi	4.63E-05	5.17E-06	8.96698	54.5601	8.56415
Parsis	Kol	Makrani	3.30E-05	3.68E-06	8.96643	54.5601	8.56415
Parsis	Balochi	Palestinians	1.97E-05	2.19E-06	8.96616	54.5601	8.56415
Parsis	French	Cambodians	1.13E-04	1.26E-05	8.96466	54.5601	8.56415
Parsis	Burmese	Sardinians	1.31E-04	1.46E-05	8.96347	54.5601	8.56415
Parsis	Bedouins	Burusho	4.11E-05	4.59E-06	8.96321	54.5601	8.56415
Parsis	Burusho	Druze	4.07E-05	4.54E-06	8.95653	54.5601	8.56415
Parsis	Sardinians	Tujia	1.37E-04	1.52E-05	8.9554	54.5601	8.56415
Parsis	Armenians	Cambodians	1.14E-04	1.27E-05	8.95534	54.5601	8.56415
Parsis	Bedouins	Makrani	2.16E-05	2.42E-06	8.95497	54.5601	8.56415
Parsis	Japanese	Druze	1.26E-04	1.41E-05	8.95265	54.5601	8.56415
Parsis	Russians	Miaozu	9.45E-05	1.06E-05	8.95243	54.5601	8.56415
Parsis	Russians	Velmas	5.88E-05	6.57E-06	8.95128	54.5601	8.56415
Parsis	Gujaratis	Sardinians	7.82E-05	8.74E-06	8.9508	54.5601	8.56415
Parsis	Georgians	Japanese	1.13E-04	1.26E-05	8.95064	54.5601	8.56415
Parsis	Druze	Yizu	1.32E-04	1.47E-05	8.95041	54.5601	8.56415
Parsis	Sardinians	She	1.40E-04	1.56E-05	8.94691	54.5601	8.56415
Parsis	Armenians	Yizu	1.20E-04	1.34E-05	8.93955	54.5601	8.56415
Parsis	Estonians	Japanese	1.01E-04	1.12E-05	8.93603	54.5601	8.56415
Parsis	Georgians	Tujia	1.16E-04	1.29E-05	8.93602	54.5601	8.56415
Parsis	Azeris	Yizu	1.01E-04	1.13E-05	8.93496	54.5601	8.56415
Parsis	Iranians	Brahui	9.30E-06	1.04E-06	8.93211	54.5601	8.56415
Parsis	Druze	Tujia	1.30E-04	1.45E-05	8.93138	54.5601	8.56415
Parsis	French	Burusho	3.41E-05	3.81E-06	8.93048	54.5601	8.56415
Parsis	Georgians	Tu	1.00E-04	1.12E-05	8.92895	54.5601	8.56415
Parsis	Russians	Tujia	9.76E-05	1.09E-05	8.92722	54.5601	8.56415
Parsis	Estonians	Tu	8.81E-05	9.87E-06	8.92625	54.5601	8.56415

Parsis	Russians	Tu	8.48E-05	9.50E-06	8.92585	54.5601	8.56415
Parsis	Georgians	Velmas	6.89E-05	7.72E-06	8.92479	54.5601	8.56415
Parsis	Han	Balochi	6.59E-05	7.38E-06	8.92177	54.5601	8.56415
Parsis	Cambodians	Tajiks	5.15E-05	5.78E-06	8.91842	54.5601	8.56415
Parsis	Sardinians	Japanese	1.35E-04	1.52E-05	8.91696	54.5601	8.56415
Parsis	Armenians	Tujia	1.18E-04	1.32E-05	8.91653	54.5601	8.56415
Parsis	Iranians	Tu	8.77E-05	9.83E-06	8.91604	54.5601	8.56415
Parsis	Russians	She	1.00E-04	1.12E-05	8.91492	54.5601	8.56415
Parsis	Burmese	Balochi	5.78E-05	6.48E-06	8.91175	54.5601	8.56415
Parsis	Druze	Mongola	1.10E-04	1.23E-05	8.90777	54.5601	8.56415
Parsis	Russians	Mongola	8.13E-05	9.13E-06	8.90286	54.5601	8.56415
Parsis	Estonians	Cambodians	9.84E-05	1.11E-05	8.90152	54.5601	8.56415
Parsis	Russians	Yizu	9.93E-05	1.12E-05	8.90039	54.5601	8.56415
Parsis	Sardinians	Yizu	1.38E-04	1.55E-05	8.89856	54.5601	8.56415
Parsis	Sardinians	Tu	1.20E-04	1.35E-05	8.89214	54.5601	8.56415
Parsis	Armenians	Mongola	9.96E-05	1.12E-05	8.88814	54.5601	8.56415
Parsis	Georgians	Burusho	3.21E-05	3.61E-06	8.8878	54.5601	8.56415
Parsis	Dai	Makrani	6.78E-05	7.63E-06	8.88329	54.5601	8.56415
Parsis	Sardinians	Cambodians	1.34E-04	1.50E-05	8.88137	54.5601	8.56415
Parsis	Balochi	Velmas	2.79E-05	3.14E-06	8.86646	54.5601	8.56415
Parsis	Armenians	Tu	1.04E-04	1.18E-05	8.86563	54.5601	8.56415
Parsis	Melanesians	Tajiks	4.82E-05	5.43E-06	8.86474	54.5601	8.56415
Parsis	Makrani	She	6.78E-05	7.64E-06	8.86436	54.5601	8.56415
Parsis	Russians	Cambodians	9.60E-05	1.08E-05	8.86428	54.5601	8.56415
Parsis	Tajiks	Tujia	5.54E-05	6.25E-06	8.86394	54.5601	8.56415
Parsis	Brahui	Tujia	6.47E-05	7.31E-06	8.85489	54.5601	8.56415
Parsis	Armenians	Japanese	1.17E-04	1.32E-05	8.85025	54.5601	8.56415
Parsis	Bedouins	Balochi	2.33E-05	2.64E-06	8.84502	54.5601	8.56415
Parsis	Brahui	Cambodians	6.23E-05	7.05E-06	8.83844	54.5601	8.56415
Parsis	Sardinians	Velmas	8.99E-05	1.02E-05	8.83799	54.5601	8.56415
Parsis	Japanese	Russians	9.65E-05	1.09E-05	8.83763	54.5601	8.56415
Parsis	Druze	Tu	1.14E-04	1.29E-05	8.82962	54.5601	8.56415
Parsis	Burmese	Brahui	5.91E-05	6.70E-06	8.82497	54.5601	8.56415
Parsis	Burusho	Estonians	2.88E-05	3.27E-06	8.82196	54.5601	8.56415
Parsis	Yorubas	Miaozu	6.44E-05	7.31E-06	8.82063	54.5601	8.56415
Parsis	Burusho	Russians	2.67E-05	3.03E-06	8.81766	54.5601	8.56415
Parsis	Mbuti_Pygmies	Miaozu	6.04E-05	6.85E-06	8.81631	54.5601	8.56415
Parsis	Sardinians	Mongola	1.16E-04	1.32E-05	8.81313	54.5601	8.56415
Parsis	Brahui	Mozabites	2.44E-05	2.77E-06	8.80896	54.5601	8.56415
Parsis	Balochi	Tujia	6.54E-05	7.43E-06	8.7941	54.5601	8.56415
Parsis	Brahui	Mongola	5.28E-05	6.01E-06	8.79182	54.5601	8.56415
Parsis	Gujaratis	Makrani	2.12E-05	2.41E-06	8.78807	54.5601	8.56415
Parsis	Bantus	Miaozu	6.50E-05	7.40E-06	8.78415	54.5601	8.56415
Parsis	Mandenkas	Miaozu	6.08E-05	6.92E-06	8.78077	54.5601	8.56415
Parsis	Iranians	Palestinians	3.99E-06	4.54E-07	8.77899	54.5601	8.56415
Parsis	Pathan	Dai	5.19E-05	5.92E-06	8.76659	54.5601	8.56415
Parsis	Sardinians	Pathan	3.96E-05	4.52E-06	8.76171	54.5601	8.56415
Parsis	Brahui	Yizu	6.40E-05	7.31E-06	8.75543	54.5601	8.56415
Parsis	Yorubas	She	7.20E-05	8.22E-06	8.75309	54.5601	8.56415
Parsis	Balochi	Mongola	5.27E-05	6.02E-06	8.75303	54.5601	8.56415
Parsis	Japanese	Brahui	6.33E-05	7.24E-06	8.7496	54.5601	8.56415
Parsis	Makrani	Miaozu	6.03E-05	6.90E-06	8.7472	54.5601	8.56415
Parsis	Brahui	Palestinians	1.86E-05	2.13E-06	8.73433	54.5601	8.56415
Parsis	Bantus	She	7.10E-05	8.13E-06	8.73218	54.5601	8.56415
Parsis	Balochi	Cambodians	6.14E-05	7.04E-06	8.73113	54.5601	8.56415
Parsis	Brahui	Melanesians	4.99E-05	5.71E-06	8.72941	54.5601	8.56415
Parsis	Han	Sindhi	4.18E-05	4.79E-06	8.72711	54.5601	8.56415
Parsis	Balochi	Yizu	6.49E-05	7.44E-06	8.72021	54.5601	8.56415
Parsis	Estonians	Pathan	2.03E-05	2.33E-06	8.71335	54.5601	8.56415
Parsis	Chamar	Mbuti_Pygmies	5.04E-05	5.79E-06	8.70706	54.5601	8.56415
Parsis	Han	Makrani	6.51E-05	7.48E-06	8.70308	54.5601	8.56415
Parsis	Mbuti_Pygmies	She	6.88E-05	7.90E-06	8.70296	54.5601	8.56415
Parsis	Mandenkas	Chamar	4.62E-05	5.31E-06	8.69527	54.5601	8.56415
Parsis	Mandenkas	She	6.62E-05	7.62E-06	8.69409	54.5601	8.56415
Parsis	Dai	Mbuti_Pygmies	7.14E-05	8.22E-06	8.68532	54.5601	8.56415
Parsis	Bedouins	Brahui	2.19E-05	2.52E-06	8.67844	54.5601	8.56415
Parsis	Pathan	She	5.12E-05	5.91E-06	8.66557	54.5601	8.56415
Parsis	Han	Mandenkas	6.46E-05	7.45E-06	8.66038	54.5601	8.56415
Parsis	Azeris	Palestinians	3.60E-06	4.16E-07	8.65821	54.5601	8.56415
Parsis	Makrani	Velmas	2.74E-05	3.17E-06	8.65407	54.5601	8.56415
Parsis	Brahui	Tu	5.33E-05	6.16E-06	8.65054	54.5601	8.56415
Parsis	Burmese	Makrani	5.82E-05	6.72E-06	8.64726	54.5601	8.56415
Parsis	Han	Bantus	6.92E-05	8.00E-06	8.64689	54.5601	8.56415
Parsis	Bantus	Dai	7.38E-05	8.54E-06	8.64072	54.5601	8.56415
Parsis	Burmese	Mandenkas	5.65E-05	6.54E-06	8.63838	54.5601	8.56415
Parsis	Burusho	She	3.56E-05	4.13E-06	8.62185	54.5601	8.56415
Parsis	Pathan	Velmas	1.71E-05	1.98E-06	8.61704	54.5601	8.56415
Parsis	Cambodians	Makrani	6.19E-05	7.19E-06	8.61289	54.5601	8.56415
Parsis	Pathan	Brahui	2.79E-06	3.24E-07	8.61188	54.5601	8.56415
Parsis	Mandenkas	Dai	6.88E-05	8.00E-06	8.60029	54.5601	8.56415
Parsis	French	Sindhi	3.35E-05	3.89E-06	8.59628	54.5601	8.56415
Parsis	Bantus	Chamar	5.13E-05	5.98E-06	8.58714	54.5601	8.56415
Parsis	Han	Yorubas	6.93E-05	8.08E-06	8.57817	54.5601	8.56415
Parsis	Pathan	Melanesians	3.80E-05	4.44E-06	8.5622	54.5601	8.56415
Parsis	Burmese	Sindhi	3.45E-05	4.03E-06	8.55869	54.5601	8.56415
Parsis	Burusho	Dai	3.72E-05	4.35E-06	8.55777	54.5601	8.56415
Parsis	Yorubas	Dai	7.31E-05	8.55E-06	8.55722	54.5601	8.56415
Parsis	Han	Pathan	4.93E-05	5.76E-06	8.55588	54.5601	8.56415
Parsis	Makrani	Yizu	6.40E-05	7.48E-06	8.5537	54.5601	8.56415
Parsis	Azeris	Bedouins	7.13E-06	8.33E-07	8.55278	54.5601	8.56415
Parsis	Armenians	Makrani	1.53E-05	1.79E-06	8.55048	54.5601	8.56415
Parsis	Druze	Makrani	1.95E-05	2.28E-06	8.54798	54.5601	8.56415
Parsis	Makrani	Tujia	6.44E-05	7.54E-06	8.54029	54.5601	8.56415
Parsis	Balochi	Japanese	6.25E-05	7.32E-06	8.5399	54.5601	8.56415
Parsis	Bantus	Yizu	6.96E-05	8.15E-06	8.53841	54.5601	8.56415

Parsis	Japanese	Makrani	6.32E-05	7.41E-06	8.53631	54.5601	8.56415
Parsis	Burmese	Bantus	6.16E-05	7.22E-06	8.53464	54.5601	8.56415
Parsis	Mandenkas	Tujia	6.52E-05	7.64E-06	8.53325	54.5601	8.56415
Parsis	Balochi	Melanesians	5.36E-05	6.29E-06	8.52822	54.5601	8.56415
Parsis	Mozabites	Tajiks	2.74E-05	3.21E-06	8.52669	54.5601	8.56415
Parsis	Han	Mbuti_Pygmies	6.73E-05	7.89E-06	8.52558	54.5601	8.56415
Parsis	Gujaratis	Burusho	7.74E-06	9.08E-07	8.51694	54.5601	8.56415
Parsis	Kol	Sindhi	1.49E-05	1.75E-06	8.51616	54.5601	8.56415
Parsis	Mozabites	Palestinians	5.93E-06	6.96E-07	8.51597	54.5601	8.56415
Parsis	Japanese	Mandenkas	6.70E-05	7.87E-06	8.51332	54.5601	8.56415
Parsis	Burmese	Pathan	4.26E-05	5.00E-06	8.51122	54.5601	8.56415
Parsis	Azeris	Brahui	9.78E-06	1.15E-06	8.50594	54.5601	8.56415
Parsis	Burmese	Yorubas	6.15E-05	7.23E-06	8.50586	54.5601	8.56415
Parsis	Makrani	Mongola	5.29E-05	6.23E-06	8.50234	54.5601	8.56415
Parsis	Balochi	Tu	5.41E-05	6.36E-06	8.49752	54.5601	8.56415
Parsis	Sardinians	Burusho	5.09E-05	6.00E-06	8.49174	54.5601	8.56415
Parsis	Pathan	Miaozu	4.52E-05	5.32E-06	8.49025	54.5601	8.56415
Parsis	Bantus	Tu	6.16E-05	7.25E-06	8.48534	54.5601	8.56415
Parsis	Japanese	Bantus	7.27E-05	8.57E-06	8.48156	54.5601	8.56415
Parsis	Kol	Mbuti_Pygmies	4.31E-05	5.08E-06	8.47234	54.5601	8.56415
Parsis	Bantus	Tujia	7.00E-05	8.27E-06	8.46951	54.5601	8.56415
Parsis	Yorubas	Chamar	5.04E-05	5.95E-06	8.46858	54.5601	8.56415
Parsis	Kol	Burusho	1.31E-05	1.55E-06	8.46368	54.5601	8.56415
Parsis	Yorubas	Yizu	6.92E-05	8.17E-06	8.46227	54.5601	8.56415
Parsis	Mandenkas	Yizu	6.49E-05	7.67E-06	8.46168	54.5601	8.56415
Parsis	Iranians	Balochi	9.90E-06	1.17E-06	8.45059	54.5601	8.56415
Parsis	Pathan	Russians	1.76E-05	2.08E-06	8.44492	54.5601	8.56415
Parsis	Palestinians	Tajiks	1.94E-05	2.29E-06	8.44075	54.5601	8.56415
Parsis	Armenians	Brahui	1.42E-05	1.68E-06	8.43647	54.5601	8.56415
Parsis	Estonians	Sindhi	2.89E-05	3.42E-06	8.43236	54.5601	8.56415
Parsis	Makrani	Melanesians	5.20E-05	6.17E-06	8.42391	54.5601	8.56415
Parsis	Cambodians	Mbuti_Pygmies	6.41E-05	7.62E-06	8.41702	54.5601	8.56415
Parsis	Cambodians	Sindhi	3.96E-05	4.71E-06	8.41619	54.5601	8.56415
Parsis	Sardinians	Sindhi	4.95E-05	5.89E-06	8.41115	54.5601	8.56415
Parsis	Bedouins	Tajiks	2.43E-05	2.89E-06	8.41089	54.5601	8.56415
Parsis	Japanese	Yorubas	7.34E-05	8.72E-06	8.40977	54.5601	8.56415
Parsis	Mandenkas	Mozabites	2.50E-05	2.98E-06	8.38016	54.5601	8.56415
Parsis	Mbuti_Pygmies	Yizu	6.54E-05	7.81E-06	8.37458	54.5601	8.56415
Parsis	Yorubas	Cambodians	6.68E-05	7.97E-06	8.37409	54.5601	8.56415
Parsis	Burmese	Mbuti_Pygmies	5.75E-05	6.87E-06	8.37064	54.5601	8.56415
Parsis	Bantus	Mongola	5.38E-05	6.43E-06	8.36416	54.5601	8.56415
Parsis	Pathan	Cambodians	4.64E-05	5.55E-06	8.36412	54.5601	8.56415
Parsis	Mbuti_Pygmies	Mongola	5.38E-05	6.43E-06	8.36275	54.5601	8.56415
Parsis	Makrani	Tu	5.31E-05	6.35E-06	8.3595	54.5601	8.56415
Parsis	Mbuti_Pygmies	Tujia	6.70E-05	8.01E-06	8.35668	54.5601	8.56415
Parsis	Mandenkas	Tu	5.80E-05	6.95E-06	8.34766	54.5601	8.56415
Parsis	Russians	Sindhi	2.60E-05	3.13E-06	8.33301	54.5601	8.56415
Parsis	Mbuti_Pygmies	Velmas	3.79E-05	4.55E-06	8.33111	54.5601	8.56415
Parsis	Mandenkas	Cambodians	6.20E-05	7.44E-06	8.33015	54.5601	8.56415
Parsis	Iranians	Azeris	2.79E-06	3.36E-07	8.32247	54.5601	8.56415
Parsis	Sindhi	Tu	3.22E-05	3.87E-06	8.31967	54.5601	8.56415
Parsis	Bantus	Cambodians	6.61E-05	7.95E-06	8.30705	54.5601	8.56415
Parsis	Yorubas	Tu	6.25E-05	7.54E-06	8.29634	54.5601	8.56415
Parsis	Burusho	Miaozu	3.17E-05	3.82E-06	8.29361	54.5601	8.56415
Parsis	Bantus	Mozabites	2.76E-05	3.32E-06	8.29265	54.5601	8.56415
Parsis	Azeris	Tajiks	9.59E-06	1.16E-06	8.28628	54.5601	8.56415
Parsis	Pathan	Yizu	4.78E-05	5.78E-06	8.27831	54.5601	8.56415
Parsis	Kol	Mandenkas	3.87E-05	4.67E-06	8.27636	54.5601	8.56415
Parsis	Kol	Bantus	4.31E-05	5.21E-06	8.26428	54.5601	8.56415
Parsis	Japanese	Mbuti_Pygmies	7.02E-05	8.50E-06	8.26065	54.5601	8.56415
Parsis	Bantus	Velmas	3.92E-05	4.75E-06	8.25133	54.5601	8.56415
Parsis	Burmese	Burusho	3.00E-05	3.65E-06	8.23101	54.5601	8.56415
Parsis	Mbuti_Pygmies	Tu	5.61E-05	6.82E-06	8.22555	54.5601	8.56415
Parsis	Georgians	Makrani	1.54E-05	1.87E-06	8.21986	54.5601	8.56415
Parsis	Druze	Tajiks	2.25E-05	2.75E-06	8.20457	54.5601	8.56415
Parsis	Pathan	Makrani	4.37E-06	5.33E-07	8.20207	54.5601	8.56415
Parsis	Japanese	Pathan	4.77E-05	5.82E-06	8.19947	54.5601	8.56415
Parsis	French	Tajiks	1.91E-05	2.34E-06	8.17637	54.5601	8.56415
Parsis	Iranians	Tajiks	1.24E-05	1.52E-06	8.14212	54.5601	8.56415
Parsis	Pathan	Tu	3.84E-05	4.72E-06	8.14034	54.5601	8.56415
Parsis	Brahui	Druze	1.89E-05	2.32E-06	8.13145	54.5601	8.56415
Parsis	French	Balochi	2.08E-05	2.56E-06	8.12981	54.5601	8.56415
Parsis	Iranians	Mozabites	1.06E-05	1.30E-06	8.12443	54.5601	8.56415
Parsis	Burusho	Cambodians	3.25E-05	4.01E-06	8.11808	54.5601	8.56415
Parsis	Kol	Yorubas	4.33E-05	5.34E-06	8.10976	54.5601	8.56415
Parsis	Gujaratis	Mbuti_Pygmies	3.32E-05	4.10E-06	8.09519	54.5601	8.56415
Parsis	Burusho	Japanese	3.25E-05	4.02E-06	8.09084	54.5601	8.56415
Parsis	Sardinians	Balochi	3.20E-05	3.96E-06	8.08653	54.5601	8.56415
Parsis	Armenians	Tajiks	1.57E-05	1.95E-06	8.07017	54.5601	8.56415
Parsis	Burusho	Tujia	3.22E-05	3.99E-06	8.06695	54.5601	8.56415
Parsis	Sardinians	Makrani	2.94E-05	3.65E-06	8.04504	54.5601	8.56415
Parsis	Estonians	Tajiks	1.61E-05	2.01E-06	8.03223	54.5601	8.56415
Parsis	Bedouins	Mbuti_Pygmies	4.31E-05	5.37E-06	8.02207	54.5601	8.56415
Parsis	Yorubas	Mozabites	2.81E-05	3.50E-06	8.02176	54.5601	8.56415
Parsis	Iranians	Mbuti_Pygmies	3.95E-05	4.93E-06	8.00807	54.5601	8.56415
Parsis	Georgians	Tajiks	1.47E-05	1.84E-06	7.98162	54.5601	8.56415
Parsis	Gujaratis	Bantus	3.19E-05	4.02E-06	7.94066	54.5601	8.56415
Parsis	Mbuti_Pygmies	Palestinians	4.20E-05	5.32E-06	7.89955	54.5601	8.56415
Parsis	Mbuti_Pygmies	Sindhi	3.17E-05	4.02E-06	7.89416	54.5601	8.56415
Parsis	Sardinians	Tajiks	3.08E-05	3.91E-06	7.87559	54.5601	8.56415
Parsis	Russians	Tajiks	1.38E-05	1.75E-06	7.87171	54.5601	8.56415
Parsis	French	Makrani	1.99E-05	2.53E-06	7.86684	54.5601	8.56415
Parsis	Georgians	Brahui	1.53E-05	1.95E-06	7.83471	54.5601	8.56415
Parsis	Mbuti_Pygmies	Melanesians	5.48E-05	6.99E-06	7.83384	54.5601	8.56415
Parsis	Azeris	Mbuti_Pygmies	3.87E-05	4.95E-06	7.81707	54.5601	8.56415

Parsis	Burusho	Mongola	2.49E-05	3.20E-06	7.78427	54.5601	8.56415
Parsis	Brahui	Mbuti_Pygmies	3.04E-05	3.92E-06	7.75806	54.5601	8.56415
Parsis	Makrani	Mbuti_Pygmies	3.18E-05	4.10E-06	7.7493	54.5601	8.56415
Parsis	Sardinians	Mbuti_Pygmies	5.75E-05	7.43E-06	7.74177	54.5601	8.56415
Parsis	Iranians	Mandenkas	3.13E-05	4.04E-06	7.74075	54.5601	8.56415
Parsis	Sardinians	Brahui	3.08E-05	3.99E-06	7.7231	54.5601	8.56415
Parsis	Armenians	Mbuti_Pygmies	5.12E-05	6.65E-06	7.70586	54.5601	8.56415
Parsis	Bedouins	Mandenkas	3.72E-05	4.83E-06	7.70287	54.5601	8.56415
Parsis	Bedouins	Bantus	4.09E-05	5.34E-06	7.65476	54.5601	8.56415
Parsis	Druze	Mbuti_Pygmies	5.31E-05	6.95E-06	7.64522	54.5601	8.56415
Parsis	Iranians	Bantus	3.59E-05	4.70E-06	7.64293	54.5601	8.56415
Parsis	Balochi	Mbuti_Pygmies	3.40E-05	4.45E-06	7.63182	54.5601	8.56415
Parsis	Bantus	Palestinians	3.93E-05	5.16E-06	7.6107	54.5601	8.56415
Parsis	Iranians	Bedouins	6.18E-06	8.13E-07	7.60453	54.5601	8.56415
Parsis	Mandenkas	Palestinians	3.50E-05	4.61E-06	7.5887	54.5601	8.56415
Parsis	Balochi	Estonians	1.79E-05	2.36E-06	7.58465	54.5601	8.56415
Parsis	Sardinians	Mandenkas	5.14E-05	6.78E-06	7.5774	54.5601	8.56415
Parsis	Estonians	Makrani	1.78E-05	2.35E-06	7.57593	54.5601	8.56415
Parsis	Mbuti_Pygmies	Tajiks	3.27E-05	4.33E-06	7.55224	54.5601	8.56415
Parsis	Azeris	Mozabites	9.10E-06	1.21E-06	7.54594	54.5601	8.56415
Parsis	Bantus	Sindhi	3.02E-05	4.00E-06	7.54483	54.5601	8.56415
Parsis	Bedouins	Yorubas	4.03E-05	5.35E-06	7.54332	54.5601	8.56415
Parsis	Iranians	Yorubas	3.51E-05	4.68E-06	7.49658	54.5601	8.56415
Parsis	Mandenkas	Druze	4.39E-05	5.85E-06	7.49633	54.5601	8.56415
Parsis	Sardinians	Bantus	5.66E-05	7.56E-06	7.48595	54.5601	8.56415
Parsis	Azeris	Sardinians	1.04E-05	1.39E-06	7.46893	54.5601	8.56415
Parsis	Bantus	Makrani	2.95E-05	3.96E-06	7.44263	54.5601	8.56415
Parsis	Russians	Makrani	1.65E-05	2.22E-06	7.43898	54.5601	8.56415
Parsis	Bantus	Druze	4.86E-05	6.54E-06	7.43533	54.5601	8.56415
Parsis	French	Brahui	1.96E-05	2.64E-06	7.43107	54.5601	8.56415
Parsis	Balochi	Russians	1.62E-05	2.18E-06	7.42816	54.5601	8.56415
Parsis	Bantus	Brahui	2.89E-05	3.91E-06	7.39312	54.5601	8.56415
Parsis	Sardinians	Yorubas	5.64E-05	7.63E-06	7.38956	54.5601	8.56415
Parsis	French	Mbuti_Pygmies	5.27E-05	7.14E-06	7.37852	54.5601	8.56415
Parsis	Armenians	Mandenkas	4.10E-05	5.56E-06	7.37505	54.5601	8.56415
Parsis	Armenians	French	5.41E-06	7.35E-07	7.36047	54.5601	8.56415
Parsis	Russians	Palestinians	1.03E-05	1.41E-06	7.3506	54.5601	8.56415
Parsis	Armenians	Bantus	4.72E-05	6.47E-06	7.29582	54.5601	8.56415
Parsis	Azeris	Bantus	3.60E-05	4.96E-06	7.27252	54.5601	8.56415
Parsis	Kol	Dai	1.38E-05	1.90E-06	7.25155	54.5601	8.56415
Parsis	Armenians	Russians	7.62E-06	1.05E-06	7.23748	54.5601	8.56415
Parsis	Balochi	Bantus	3.13E-05	4.32E-06	7.23537	54.5601	8.56415
Parsis	Georgians	Mandenkas	4.05E-05	5.60E-06	7.22058	54.5601	8.56415
Parsis	Armenians	Estonians	8.97E-06	1.24E-06	7.21762	54.5601	8.56415
Parsis	Estonians	Mbuti_Pygmies	4.80E-05	6.65E-06	7.21605	54.5601	8.56415
Parsis	Iranians	Sardinians	1.16E-05	1.61E-06	7.20559	54.5601	8.56415
Parsis	Bedouins	Russians	1.25E-05	1.74E-06	7.20448	54.5601	8.56415
Parsis	Georgians	Bantus	4.77E-05	6.63E-06	7.19616	54.5601	8.56415
Parsis	Russians	Mbuti_Pygmies	4.73E-05	6.58E-06	7.18754	54.5601	8.56415
Parsis	Estonians	Brahui	1.69E-05	2.35E-06	7.17915	54.5601	8.56415
Parsis	Armenians	Yorubas	4.62E-05	6.44E-06	7.1765	54.5601	8.56415
Parsis	French	Mandenkas	4.56E-05	6.40E-06	7.12475	54.5601	8.56415
Parsis	Sardinians	Nogais	1.84E-05	2.59E-06	7.11903	54.5601	8.56415
Parsis	Estonians	Palestinians	1.06E-05	1.49E-06	7.11431	54.5601	8.56415
Parsis	Russians	Druze	1.02E-05	1.44E-06	7.10795	54.5601	8.56415
Parsis	Russians	Brahui	1.54E-05	2.16E-06	7.10717	54.5601	8.56415
Parsis	Bantus	Tajiks	3.08E-05	4.36E-06	7.07084	54.5601	8.56415
Parsis	French	Bantus	5.16E-05	7.30E-06	7.06169	54.5601	8.56415
Parsis	Makrani	Nogais	7.62E-06	1.08E-06	7.05406	54.5601	8.56415
Parsis	Balochi	Nogais	6.74E-06	9.57E-07	7.0438	54.5601	8.56415
Parsis	Sardinians	Russians	1.18E-05	1.68E-06	7.01085	54.5601	8.56415
Parsis	Georgians	Yorubas	4.71E-05	6.73E-06	7.00574	54.5601	8.56415
Parsis	Estonians	Druze	1.12E-05	1.61E-06	6.99884	54.5601	8.56415
Parsis	French	Yorubas	4.98E-05	7.15E-06	6.96142	54.5601	8.56415
Parsis	Burusho	Bantus	2.98E-05	4.32E-06	6.89294	54.5601	8.56415
Parsis	Estonians	Mandenkas	3.88E-05	5.69E-06	6.81731	54.5601	8.56415
Parsis	Estonians	Bantus	4.48E-05	6.58E-06	6.80775	54.5601	8.56415
Parsis	Bedouins	Estonians	1.24E-05	1.82E-06	6.80327	54.5601	8.56415
Parsis	Mandenkas	Russians	3.69E-05	5.43E-06	6.80088	54.5601	8.56415
Parsis	Russians	Bantus	4.26E-05	6.27E-06	6.79226	54.5601	8.56415
Parsis	Brahui	Tajiks	4.89E-06	7.23E-07	6.76479	54.5601	8.56415
Parsis	Balochi	Tajiks	4.61E-06	6.85E-07	6.73805	54.5601	8.56415
Parsis	French	Sardinians	5.57E-06	8.28E-07	6.73135	54.5601	8.56415
Parsis	Russians	Mozabites	1.23E-05	1.83E-06	6.69911	54.5601	8.56415
Parsis	Estonians	Yorubas	4.30E-05	6.42E-06	6.69732	54.5601	8.56415
Parsis	Russians	Yorubas	4.13E-05	6.18E-06	6.6831	54.5601	8.56415
Parsis	French	Mozabites	1.15E-05	1.72E-06	6.67954	54.5601	8.56415
Parsis	Makrani	Tajiks	5.31E-06	7.96E-07	6.67209	54.5601	8.56415
Parsis	Azeris	French	7.39E-06	1.11E-06	6.63819	54.5601	8.56415
Parsis	Bedouins	French	8.91E-06	1.34E-06	6.6324	54.5601	8.56415
Parsis	Estonians	Mozabites	1.26E-05	1.91E-06	6.62485	54.5601	8.56415
Parsis	Mandenkas	Nogais	2.47E-05	3.75E-06	6.59955	54.5601	8.56415
Parsis	French	Druze	5.74E-06	8.73E-07	6.57292	54.5601	8.56415
Parsis	Sardinians	Estonians	9.84E-06	1.50E-06	6.56174	54.5601	8.56415
Parsis	Georgians	Estonians	1.07E-05	1.64E-06	6.53138	54.5601	8.56415
Parsis	Yorubas	Nogais	2.75E-05	4.23E-06	6.49501	54.5601	8.56415
Parsis	Georgians	French	6.74E-06	1.04E-06	6.46688	54.5601	8.56415
Parsis	Georgians	Sardinians	9.33E-06	1.45E-06	6.41041	54.5601	8.56415
Parsis	Georgians	Russians	8.38E-06	1.32E-06	6.34333	54.5601	8.56415
Parsis	Iranians	French	6.74E-06	1.06E-06	6.33227	54.5601	8.56415
Parsis	Azeris	Estonians	7.57E-06	1.20E-06	6.29105	54.5601	8.56415
Parsis	Iranians	Estonians	7.93E-06	1.27E-06	6.24676	54.5601	8.56415
Parsis	Azeris	Russians	6.31E-06	1.02E-06	6.20765	54.5601	8.56415
Parsis	Iranians	Russians	6.83E-06	1.13E-06	6.02251	54.5601	8.56415
Parsis	Armenians	Iranians	2.96E-06	5.41E-07	5.46193	54.5601	8.56415

Table S8. Analyses of functional variants over the 99.9th percentile of the distribution

CHR	SNP	Ancestral	Derived	Position	Iranians	Gujaratis	Parsis	PBSold	Uploaded_variatior	Location	Consequence	SYMBOL	Gene	SIFT	PolyPhen	PUBMED
1	rs2230301	T	G	220197625	0.1053	0.0941	0.3864	0.227797252	rs2230301	1:220024283-220024283	missense_variant	EPRS	ENSG00000136628	tolerated(1)	benign(0.001)	25310850
6	rs382259	A	G	32209027	0.5789	0.4851	0.8523	0.225229844	rs382259	6:32241250-32241250	intergenic_variant	-	-	-	-	19846760,22194982
3	rs1915087	C	T	121838791	0.4737	0.4406	0.8068	0.257969966	rs1915087	3:122119944-122119944	3_prime_UTR_variant	CD86	ENSG00000114013	-	-	19852851,25912130
17	rs3764383	T	C	76208851	0.3158	0.3713	0.0682	0.228642038	rs3764383	17:78212770-78212770	upstream_gene_variant	BIRC5	ENSG00000089685	-	-	20881643,20057973
12	rs1866074	A	G	104374442	0.4474	0.4257	0.1364	0.221496463	rs1866074	12:103980664-103980664	intron_variant	TDG	ENSG00000139372	-	-	23565320,19403629
12	rs4135054	C	T	104363610	0.1316	0.104	0.4091	0.222879948	rs4135054	12:103969832-103969832	intron_variant	TDG	ENSG00000139372	-	-	23565320,19403629,23504502
6	rs9261129	G	A	29979579	0.8684	0.8812	0.4886	0.352032542	rs9261129	6:30011802-30011802	downstream_gene_variant	ZNRD1-AS1	ENSG00000204623	-	-	20041166,17641165,18495769,18982067,19693088
6	rs2301753	A	C	30039240	0.8684	0.8724	0.5	0.321100619	rs2301753	6:30071463-30071463	downstream_gene_variant	PPP1R11	ENSG00000204619	-	-	20041166,17641165,18495769,18982067,24842830,19693088
6	rs2074480	T	G	30040810	0.1316	0.1188	0.5114	0.352032542	rs2074480	6:30073033-30073033	downstream_gene_variant	PPP1R11	ENSG00000204619	-	-	20041166,17641165,18495769,18982067,24842830,19693088
6	rs2074479	G	A	30041009	0.8684	0.8812	0.4886	0.352032542	rs2074479	6:30073232-30073232	downstream_gene_variant	PPP1R11	ENSG00000204619	-	-	20041166,17641165,18495769,18982067,24842830,19693088
6	rs7758512	C	A	29970589	0.8684	0.8812	0.4886	0.352032542	rs7758512	6:30002812-30002812	upstream_gene_variant	HLA-J	ENSG00000204622	-	-	20041166,19197363,17641165,18495769,18982067,24842830
6	rs3869068	A	G	30004052	0.8684	0.8812	0.4886	0.352032542	rs3869068	6:30036275-30036275	upstream_gene_variant	ZNRD1-AS1	ENSG00000204623	-	-	20041166,21221856,17641165,18495769,18982067,24842830,19693088
5	rs1422795	T	C	156936364	0.5526	0.495	0.2045	0.217718397	rs1422795	5:157509356-157509356	missense_variant	ADAM19	ENSG00000135074	tolerated(0.23)	benign(0.03)	23267696,21659657,22424883,21304900,19319892,20010835,24951661,21784901
6	rs9261174	T	C	29996855	0.132	0.1111	0.511	0.3619103	rs9261174	6:30029078-30029078	intron_variant,non_coding_transcript_variant	ZNRD1-AS1	ENSG00000204623	-	-	20041166,20552027,22474614,19107206,19679225,17641165,18495769,18982067,24842830,19693088,20976252
3	rs1129055	G	A	1.22E+08	0.5	0.4653	0.136	0.28395028	rs1129055	3:122119472-122119472	missense_variant	CD86	ENSG00000114013	tolerated(0.09)	benign(0.015)	19852851,20732370,24298899,25369324,20230296,21870962,22821131,25129060,21090563,22123319,17513529,25505553,16223675

Table S9. The control and conding region mtDNA mutations for Indian and Pakistani Parsi populations

Country	Sample Id	HVS-I (-16000nt)	HVS-II and coding region	Haplogroup	Reference
India	Z176	223-304	73-199-263-489-12561-15928	M35	Present study
India	Z177	086-318T	73-152-263-980-1811-12308-12372	U7	Present study
India	Z178	223-304	73-199-263-489-12561-15928	M35	Present study
India	Z179	129-144A-223	73-263-489-1811	M5	Present study
India	Z180	223-311	73-263-482-489	M3	Present study
India	Z181	126-223	73-263-482-489	M3	Present study
India	Z182	209-223-234	73-195A-263-489-12007-15431	M30	Present study
India	Z183	356	73-263-195-499	U4	Present study
India	Z185	223-234	73-195A-263-489-12007-15431	M30	Present study
India	Z186	223-304	73-199-263-489-12561-15928	M35	Present study
India	Z187	223-234	73-195A-263-489-12007-15431	M30	Present study
India	Z188	217-309	72-73-152-195-263	HV2	Present study
India	Z189	217-309	72-73-152-195-263	HV2	Present study
India	Z190	126-163-186-189-239-243-294-362	73-263-709-15928	T1	Present study
India	Z191	167-223-304	73-199-263-489-12561-15928	M35	Present study
India	Z192	129-309-318T	73-152-263-980-1811-12308-12372	U7	Present study
India	E1	356	11467-12308	U4	Present study
India	E2	168-223-239	2706-8701-9540-10873-11719-14766	H	Present study
India	E3	111-168-223-192-264-275-300-352	15043-12705	M52	Present study
India	E4	111-168-223-192-264-275-300-352	15043-12705	M52	Present study
India	E5	126-223	482-4703-15043	M3	Present study
India	E6	168-239	2706-8701-9540-10873-11719-14766	H	Present study
India	E7	051-168-234	9540-11467-12308-15061	U2c	Present study
India	E8	086-209-223	195A-12007-15043	M30	Present study
India	E9	223-356	195A-12007-15043	M30	Present study
India	E10	356	11467-12308	U4	Present study
India	E11	223-234	195A-12007-15043	M30	Present study
India	E12	086-209-223-278	195A-12007-15043	M30	Present study
India	F1	356	1467-8701-9540-12308	U4	Present study
India	F2	169-172-262	15043	M33	Present study
India	F3	223-234	195A-12007-15043	M30	Present study
India	F4	223-234	195A-12007-15043	M30	Present study
India	F5	126-223	482-15043	M3	Present study
India	F6	223-234	195A-12007-15043	M30	Present study
India	F7	168-239	2706-8701-9540-10873-11719-14766	H	Present study
India	F8	086-209-223-278	195A-12007-15043	M30	Present study
India	F9	185-223-270-274-319-352	8502	M2	Present study
India	F10	no mutation	8701-9540-11719-14766	H	Present study
India	F11	223-234	195A-12007-15043	M30	Present study
India	G1	356	11467-12308	U4	Present study
India	G2	169-172-262-278-223-355	15043	M33	Present study
India	G3	223-234-304-359	15043-15938	M39	Present study
India	G4	126	482-15043	M3	Present study
India	G5	126	482-15043	M3	Present study
India	G6	223-234	195A-12007-15043	M30	Present study
India	G7	223	15043	M	Present study
India	G8	274	9540-10873-11719-12705-14766	H	Present study
India	G10	126-223	482-4703-15043	M3	Present study
India	G11	292	709-750-7094	HV4	Present study
India	G12	223-304	8701	M35	Present study
India	H1	356	8701-9540-11467	U4	Present study
India	H2	no mutation	8701-9540-11719-14766	H	Present study
India	H3	no mutation	2706-11719-12705-14766	H	Present study
India	H4	168-239	8701	R0	Present study
India	H5	126-223	482-4703-15043	M3	Present study
India	H6	223-234	195A-12007-15043	M30	Present study
India	H7	223-234	195A-12007-15043	M30	Present study
India	H8	051-129-362	9540-11467-12308	U2e	Present study
India	H9	356	9540-11467-12308	U4	Present study
India	H10	311-343	11467-12308	U3	Present study
India	H11	217-309	10873-12705-14766	HV2	Present study
India	H12	356	9540-11467-12308-12372	U4	Present study
India	PAR_1	126-223	73-4580-10398-10400	M3	Metspalu et al. 2004
India	PAR_10	126-296-362	73-15607	T2	Metspalu et al. 2004
India	PAR_11	223-304-311	73-10398-10400	M35	Metspalu et al. 2004
India	PAR_12	356	73-12308	U4	Metspalu et al. 2004
India	PAR_13	126-223	73-4580-10398-10400	M3	Metspalu et al. 2004
India	PAR_14	223-311	73-4580-10398-10400	M3	Metspalu et al. 2004
India	PAR_15	126-223	73-4580-10398-10400	M3	Metspalu et al. 2004
India	PAR_16	126-223-311	73-4580-10398-10400	M3	Metspalu et al. 2004
India	PAR_17	223-234	73-10398-10400	M30	Metspalu et al. 2004
India	PAR_18	51-179-234-240C-247-278	73-12308	U2c	Metspalu et al. 2004
India	PAR_19	223-311	73-4580-10398-10400	M3	Metspalu et al. 2004
India	PAR_2	126-223	73-4580-10398-10400	M3	Metspalu et al. 2004
India	PAR_20	111-168-192-223-264-275-300-352	73-10398-10400	M52	Metspalu et al. 2004
India	PAR_21	126-163-186-189-239-243-294-362	73-15607	T1	Metspalu et al. 2004
India	PAR_22	223-270-319-352	73-10398-10400	M2	Metspalu et al. 2004
India	PAR_23	126-223-311	73-4580-10398-10400	M3	Metspalu et al. 2004
India	PAR_24	129-223-265C	73-10398-10400	M5	Metspalu et al. 2004
India	PAR_25	93-126-163-186-189-294	73-15607	T1	Metspalu et al. 2004
India	PAR_26	223-311	73-4580-10398-10400	M3	Metspalu et al. 2004
India	PAR_27	126-223-311	73-4580-10398-10400	M3	Metspalu et al. 2004
India	PAR_28	129-309-318T	73-12308	U7	Metspalu et al. 2004
India	PAR_29	126-223-311	73-4580-10398-10400	M3	Metspalu et al. 2004
India	PAR_3	311-343	73-12308	U3	Metspalu et al. 2004

India	PAR_30	126-296-362	73-15607	T2	Metspalu et al. 2004
India	PAR_31	223-270-319-352	73-10398-10400	M2	Metspalu et al. 2004
India	PAR_32	223-311	73-4580-10398-10400	M3	Metspalu et al. 2004
India	PAR_33	309-318T	73-12308	U7	Metspalu et al. 2004
India	PAR_34	217-309		HV2	Metspalu et al. 2004
India	PAR_35	129-186-223-224	73-10398-10400	M5	Metspalu et al. 2004
India	PAR_36	223-275-327A-362	73-10398-10400	M52	Metspalu et al. 2004
India	PAR_37	223-304	73-10398-10400	M35	Metspalu et al. 2004
India	PAR_38	168-239		H	Metspalu et al. 2004
India	PAR_39	126-223	73-4580-10398-10400	M3	Metspalu et al. 2004
India	PAR_4	129-309-318T	73-12308	U7	Metspalu et al. 2004
India	PAR_40	223-311	73-4580-10398-10400	M3	Metspalu et al. 2004
India	PAR_41	126-296-362	73-15607	T2	Metspalu et al. 2004
India	PAR_42	223-304-359	73-10398-10400	M35	Metspalu et al. 2004
India	PAR_43	182-183-189-223-311-362	73-4580-10398-10400	M3	Metspalu et al. 2004
India	PAR_44	223-311	73-4580-10398-10400	M3	Metspalu et al. 2004
India	PAR_45	356	73-12308	U4	Metspalu et al. 2004
India	PAR_46	223-304	73-10398-10400	M35	Metspalu et al. 2004
India	PAR_47	51-129C-183-194+C-362	73-12308	U2e	Metspalu et al. 2004
India	PAR_48	86-209-223-278	73-10398-10400	M30	Metspalu et al. 2004
India	PAR_49	223-234	73-10398-10400	M30	Metspalu et al. 2004
India	PAR_5	223-311	73-4580-10398-10400	M3	Metspalu et al. 2004
India	PAR_50	223-311	73-4580-10398-10400	M3	Metspalu et al. 2004
India	PAR_51	126-223	73-4580-10398-10400	M3	Metspalu et al. 2004
India	PAR_52	126-223	73-4580-10398-10400	M3	Metspalu et al. 2004
India	PAR_53	223-270-319-352	73-10398-10400	M2	Metspalu et al. 2004
India	PAR_54	223-304-359	73-10398-10400	M35	Metspalu et al. 2004
India	PAR_55	126-187-296-362	73-15607	T2	Metspalu et al. 2004
India	PAR_56	84-124-184		H	Metspalu et al. 2004
India	PAR_6	217		HV2	Metspalu et al. 2004
India	PAR_8	217-274-309		HV2	Metspalu et al. 2004
India	PAR_9	126-223-311	73-4580-10398-10400	M3	Metspalu et al. 2004
Pakistan	PaPRS001	356-519	73-195-263-499	U4	Present study
Pakistan	PaPRS002	126-223-311-519	73-204-217-263-482-489	M3	Present study
Pakistan	PaPRS003	223-304-359	55+T-59d-60d-65+T-66-73-153-263-463-485-489	M39	Present study
Pakistan	PaPRS004	356-519	73-195-263-499	U4	Present study
Pakistan	PaPRS005	126-296-362-519	73-263-522-23d	T2	Present study
Pakistan	PaPRS006	223-304	55+T-59d-60d-65+T-66-73-153-263-463-485-489	M39	Present study
Pakistan	PaPRS007	093-126-163-186-189-294-519	73-152-195-263	T1	Present study
Pakistan	PaPRS008	145-182C-183C-189-249-519	73-263-285-385-522-23d-573+3C	U1	Present study
Pakistan	PaPRS009	223-304	55+T-59d-60d-65+T-66-73-153-263-463-485-489	M39	Present study
Pakistan	PaPRS010	129-309-318T-519	73-151-152-263-522-23d	U7	Present study
Pakistan	PaPRS011	356-519	73-195-263-499	U4	Present study
Pakistan	PaPRS012	223-311-519	73-151-152-263-482-489	M3	Present study
Pakistan	PaPRS013	086-209-223-278-519	72-152-195A-207-263-489-522-23d	M30	Present study
Pakistan	PaPRS014	182C-183C-189-249-390R-519	73-263-285-385-522-23d-573+3C	U1	Present study
Pakistan	PaPRS015	217-309	72-73-152-195-263	HV2	Present study
Pakistan	PaPRS016	356-519	73-195-263-499-524+2C	U4	Present study
Pakistan	PaPRS017	179-223-519	73-195A-263-489-522-23d	M30	Present study
Pakistan	PaPRS018	223-304	55+T-59d-60d-65+T-66-73-153-263-463-485-489	M39	Present study
Pakistan	PaPRS019	129-223-265C-519	73-263-489	M5	Present study
Pakistan	PaPRS020	126-223-519	73-200-204-235-263-482-489	M3	Present study
Pakistan	PaPRS021	126-223-519	73-195-200-204-235-263-482-489	M3	Present study
Pakistan	PaPRS022	126-163-186-189-239-243-294-362-519	73-263	T1	Present study
Pakistan	PaPRS023	093Y-126-163-186-189-294-519	73-152-195-263	T1	Present study
Pakistan	PaPRS024	356-519	73-195-263-499-524+2C	U4	Present study
Pakistan	PaPRS025	223-234-519	73-195A-263-485-489-522-23d	M30	Present study
Pakistan	PaPRS026	126-223-311-519	73-204-217-263-482-489	M3	Present study
Pakistan	PaPRS027	223-275-327A-362-390-519	73-263-489-522-23d-573+3C	M52	Present study
Pakistan	PaPRS028	168-239	263	R0	Present study
Pakistan	PaPRS029	223-304-359	55+T-59d-60d-65+T-66-73-153-263-463-485-489	M39	Present study
Pakistan	PaPRS030	126-296-362	73-263-522-23d	T2	Present study
Pakistan	PaPRS031	111-168-189-192-223-264-275-300-352-519-525	73-146-150-263-489	M52	Present study
Pakistan	PaPRS032	217-309	72-73-152-195-263	HV2	Present study
Pakistan	PaPRS033	234-519	73-195-263-485-489-522-23d	M30	Present study
Pakistan	PaPRS034	223-234-519	73-195A-263-385R-485-489-522-23d	M30	Present study
Pakistan	PaPRS035	217-309	72-73-152-195-263	HV2	Present study
Pakistan	PaPRS036	217-309	72-73-152-263	HV2	Present study
Pakistan	PaPRS037	223-304	55+T-59d-60d-65+T-66-73-153-263-463-485-489	M39	Present study
Pakistan	PaPRS038	309-318T-519	73-151-152-263-522-23d	U7	Present study
Pakistan	PaPRS039	217-519	73-152-195-263	HV2	Present study
Pakistan	PaPRS040	182C-183C-189-249-519	73-263-285-385-522-23d-573+3C	U1	Present study
Pakistan	PaPRS041	209-223-234-519	73-195A-263-485-489-522-23d	M30	Present study
Pakistan	PaPRS042	086-153-223-319-519	73-189-199-263-267	N2a	Present study
Pakistan	PaPRS043	129-309-318T-519	73-151-152-263-522-23d	U7	Present study
Pakistan	PaPRS044	111-168-192-223-264-275-300-352-519-525	73-146-150-263-489	M52	Present study
Pakistan	PaPRS045	189-192-221-223-278-325-519	73-152-195A-263-489-522-23d	M30	Present study
Pakistan	PaPRS046	111-168-192-223-264-275-300-352-519-525	73-146-150-263-489	M52	Present study
Pakistan	PaPRS047	217-309	72-73-152-195-263	HV2	Present study
Pakistan	PaPRS048	126-223-519	73-200-204-235-263-482-489	M3	Present study
Pakistan	PaPRS049	168-239-519	263	R0	Present study
Pakistan	PaPRS050	217-309	72-73-152-195-263	HV2	Present study

Table S10. The detailed available Y chromosome genotype frequency of Indian and Pakistani Parsi groups

Pakistan Parsi *	N= 90	Indian Parsi (pool 1) N=46		Indian Parsi (pool 2) N=38	
E-M123	0.056	E-M123	0.022	E-YAP	0.105
G2-P15	0.011	G-U2	0.022	G-M201	0.026
H1-M52	0.022	H1a-M2972	0.022	J-12f2	0.026
J2a-M410	0.389	J2a-M410	0.304	J2-M12	0.079
L-M20	0.033	J2b-Z1827	0.043	J2-M410	0.289
L-M27	0.011	L-M20	0.022	K-M9	0.026
L-M317	0.133	L-M27	0.022	L-M20	0.237
R-M207	0.011	L-M317	0.152	P	0.053
R1-M173	0.044	P-M45	0.022	R1a-M17	0.132
R1a-M17	0.078	R1a-M417	0.087	R1-M173	0.026
R2-M124	0.211	R1a-M634	0.130		
		R1-M173	0.043		
		R2a-P267	0.109		

Table S11. List of diagnostic sites and their results on mtDNA genotyped among ancient Parsi samples

Id	149	207	249	482	709	1391	1442	1453	1524	1541	1598	1736	1811	1888	2045	2755	3010	3348	3360	3384	3434	3543	3714	3817	4086	4140	4216	4491	4703	4775	4907	5178	5301	5783	5823	6305	6371	6620	7094	7859	8014	8414	8502	8697	8701	9064	9180	9540	9581	9824	10550	10609	10727	10754	11023	11083	11176	11215	11467	11719	12007	12133	12189	12285	12308	12361	12406	12612	12618	12633	12633	12705	12810	12879	12950	13104	13111	13135	13167	13188	13263	13359	13710	13759	13928	14110	14569	14766	14800	14905	15043	15440	15452	15530	15607	15670	15754	16069	16071	16108	16111	16126	16172	16223	16274	16288	16294	16344	Hg
	T	G	A	T	G	T	G	A	A	T	G	A	A	G	A	A	G	A	A	A	A	C	A	C	C	C	T	G	T	A	T	C	A	G	A	G	C	T	T	G	A	C	A	G	A	G	A	C	T	T	A	T	C	A	A	A	G	C	A	G	G	C	T	T	A	A	G	A	G	C	C	C	A	T	A	A	T	G	A	C	A	G	A	G	G	T	G	C	C	G	G	T	C	T	A	T	C	C	C	C	C	T	T	C	G	T	C	C	
SN1	.	.	.	C	.	.	.	.	.	.	.	.	.	.	.	.	.	.	.	.	.	.	.	.	.	.	.	.	C	.	.	.	.	.	.	.	.	.	.	.	.	.	.	.	G	.	.	.	.	.	.	.	.	.	.	.	.	.	.	A	.	.	.	.	.	.	.	.	.	.	.	T	.	.	.	.	.	.	.	.	.	.	.	.	.	.	.	T	.	.	A	.	.	.	.	.	.	.	.	.	.	C	.	T	.	.	.	.	M3
SN2	.	.	.	.	.	.	.	.	.	.	.	.	.	.	.	.	.	.	.	.	.	.	.	.	.	.	.	.	.	.	.	.	.	.	.	.	.	.	.	.	.	.	.	.	.	.	.	T	.	.	.	.	.	.	.	.	.	.	.	.	.	.	.	.	.	.	.	.	.	.	.	.	.	.	.	.	.	.	.	.	.	.	.	.	.	.	.	.	.	.	.	.	.	.	.	.	.	.	.	.	.	.	.	.	.	.	.	.	HV
SN3	.	.	.	C	.	.	.	.	.	.	C	.	.	.	.	.	.	.	.	.	.	.	.	.	.	.	.	.	C	.	.	.	.	.	.	.	.	.	.	.	.	.	.	C	G	C	.	.	.	.	.	.	.	.	.	.	.	.	.	A	.	.	.	.	.	.	.	.	.	.	.	T	.	.	.	.	.	.	.	.	.	.	.	.	.	.	.	T	.	.	A	.	.	.	.	.	.	.	.	.	.	C	.	T	.	.	.	.	M3
SN4	.	.	.	.	.	.	.	.	.	.	.	.	.	.	.	.	.	.	.	.	.	.	.	.	.	.	.	A	.	.	.	.	.	.	.	.	T	.	.	.	.	.	.	.	.	.	.	T	.	.	.	.	.	.	.	.	.	.	.	A	.	.	.	.	.	.	.	.	.	.	.	T	.	.	.	.	.	A	.	.	.	.	.	.	.	.	.	T	.	.	.	.	.	.	.	.	.	.	.	.	.	.	.	T	.	.	.	.	X
SN11	.	.	.	.	.	.	.	.	.	.	C	.	.	.	.	.	.	.	.	.	.	.	.	.	.	.	.	A	.	.	.	.	.	.	.	.	T	.	.	.	.	.	.	.	.	.	.	T	.	.	.	.	.	.	.	.	.	.	.	A	.	.	.	.	.	.	.	.	.	.	.	T	.	.	.	.	.	A	.	.	.	.	.	.	.	.	.	T	.	.	.	.	.	.	.	.	.	.	.	.	.	.	.	T	.	.	.	.	X
SN12	.	.	.	.	.	.	.	.	.	.	.	.	G	.	.	.	.	.	.	.	.	.	.	.	.	.	.	.	.	.	.	.	.	.	.	.	.	.	.	.	.	.	.	.	.	.	.	T	.	.	.	.	.	.	.	.	.	.	G	A	.	.	.	.	G	.	.	.	.	.	.	.	.	.	.	.	.	.	.	.	.	.	.	.	.	.	A	T	.	.	.	.	.	.	.	.	.	.	.	.	.	.	.	.	.	.	T	.	U7
SN13	.	.	.	.	.	.	.	.	.	.	.	.	.	.	.	.	.	.	.	.	.	.	.	.	.	.	.	.	.	.	.	.	.	.	.	.	.	.	.	.	.	.	.	.	.	.	.	T	.	.	.	.	.	G	.	.	.	.	.	A	.	.	.	.	.	G	.	.	.	.	.	.	.	.	.	.	.	.	.	T	.	.	.	.	.	.	.	T	.	.	.	.	.	.	.	.	.	.	.	.	.	.	.	.	.	.	.	.	R5
SN14	.	.	.	.	.	.	.	.	.	.	.	.	G	.	.	.	.	.	.	.	.	.	.	.	.	.	.	.	.	.	.	.	.	.	.	.	.	.	.	.	.	.	.	.	.	.	.	T	.	.	.	.	.	.	.	.	.	.	G	A	.	.	.	.	G	.	.	.	.	.	.	.	.	.	.	.	.	.	.	.	.	.	.	.	.	.	.	T	.	.	.	.	.	.	.	.	.	.	.	.	.	.	.	.	.	.	.	.	U7
SN15	.	A	.	.	A	.	.	.	.	.	.	.	.	A	.	.	.	.	.	.	.	.	.	.	.	.	.	.	.	.	.	.	.	.	.	.	.	.	.	.	.	.	.	.	G	.	.	.	.	.	.	.	.	.	.	.	.	.	.	A	.	.	.	.	.	.	.	.	.	.	.	T	.	.	.	.	.	.	.	.	.	.	.	.	.	.	.	T	.	.	A	.	.	.	.	.	.	.	.	.	.	.	.	T	.	.	.	.	M5
SN16	.	.	.	.	.	.	.	.	.	.	.	.	.	.	.	.	.	.	.	.	.	.	.	.	.	.	C	.	.	.	.	.	.	.	.	.	.	.	.	.	.	.	.	.	.	.	.	T	.	.	.	.	.	.	.	.	.	.	.	A	.	.	.	.	.	.	.	.	.	.	.	.	.	.	.	.	.	.	.	.	.	.	.	.	.	.	.	T	.	.	.	.	.	.	.	.	.	.	.	.	.	C	.	.	.	.	.	.	T
SN5	.	.	.	.	.	.	.	.	.	.	.	.	.	.	.	.	.	.	.	.	.	.	.	A	A	.	C	.	.	.	.	.	.	.	.	.	.	.	.	.	.	.	.	.	.	.	.	T	.	.	.	.	.	.	.	.	A	.	.	A	.	.	.	.	.	.	.	.	.	.	.	.	.	.	.	.	.	.	.	.	.	.	.	.	.	.	.	T	.	.	.	.	.	.	.	.	.	.	.	.	.	C	.	.	.	C	.	.	T2
SN6	.	.	.	.	.	.	.	.	.	.	.	.	.	.	.	.	.	.	.	.	.	.	.	.	.	.	.	.	.	.	.	.	.	.	.	.	.	.	.	.	.	.	.	.	.	.	.	T	.	.	.	.	.	.	.	.	.	.	G	A	.	.	.	.	G	.	.	.	.	.	.	.	.	.	.	.	.	.	.	.	.	.	.	.	.	.	.	T	.	.	.	.	.	.	.	.	.	.	.	.	.	.	.	.	.	.	.	.	U
SN7	.	.	.	.	.	.	.	.	.	.	.	.	.	.	.	.	.	.	.	.	.	.	.	.	.	.	.	.	.	.	.	.	.	.	.	.	.	.	.	.	.	.	.	.	G	.	.	.	.	.	.	.	.	.	.	G	.	.	.	A	.	.	.	.	.	.	.	.	.	.	.	T	.	.	.	.	.	.	.	.	.	.	.	.	.	.	.	T	.	.	A	.	.	.	.	C	.	.	.	.	.	.	.	.	.	.	.	.	M2
SN8	.	.	.	.	.	.	.	.	.	.	.	.	.	.	.	.	.	.	.	.	.	.	.	.	.	.	.	.	.	.	.	.	.	.	.	.	.	.	.	.	.	.	.	.	.	.	.	T	.	.	.	.	.	.	.	.	.	.	.	A	.	.	.	.	.	.	.	.	.	.	.	.	.	.	.	.	.	.	.	.	.	.	.	.	.	.	.	T	.	.	.	.	.	.	.	.	.	.	T	.	.	.	.	.	.	.	.	.	R2
SN9	.	.	.	.	.	.	.	.	.	.	.	.	.	.	.	.	.	.	.	.	.	.	.	.	T	.	.	.	.	.	.	.	.	.	.	.	.	.	.	.	.	.	.	.	.	.	.	T	.	.	.	.	.	.	.	.	.	.	.	A	.	.	.	.	.	.	.	.	.	.	.	.	.	.	.	.	.	.	.	T	.	.	.	.	.	.	.	T	.	.	.	.	.	.	.	.	.	.	.	T	.	C	.	.	.	.	.	.	R0
SN10	.	.	.	C	.	.	.	.	.	.	.	.	.	.	.	.	.	.	.	.	.	.	.	.	.	.	.	.	C	.	.	.	.	.	.	.	.	.	.	.	.	.	.	.	G	.	.	.	.	.	.	.	.	.	.	.	.	.	.	A	.	.	.	.	.	.	.	.	.	.	.	T	.	.	.	.	.	.	.	.	.	.	.	.	.	.	.	T	.	.	A	.	.	.	.	.	.	.	.	.	.	C	.	T	.	.	.	.	M3
SN17	.	.	.	.	.	.	.	.	.	.	.	.	G	.	.	.	.	.	.	.	.	.	.	.	.	.	.	.	.	.	.	.	.	.	.	.	.	.	.	.	.	.	.	.	.	.	.	T	.	.	.	.	.	.	.	.	.	.	G	A	.	.	.	.	G	.	.	.	.	.	.	.	.	.	.	.	.	.	.	.	.	.	.	.	.	.	A	T	.	.	.	.	.	.	.	.	.	.	.	.	.	.	.	.	.	.	.	.	U7
SN19	.	.	.	C	.	.	.	.	.	.	.	.	.	.	.	.	.	.	.	.	.	.	.	.	.	.	.	.	C	.	.	.	.	.	.	.	.	.	.	.	.	.	.	.	G	.	.	.	.	.	.	.	.	.	.	.	.	.	.	A	.	.	.	.	.	.	.	.	.	.	.	T	.	.	.	.	.	.	.	.	.	.	.	.	.	.	.	T	.	.	A	.	.	.	.	.	.	.	.	.	.	C	.	T	.	.	.	.	M3
SN18	.	.	.	C	.	.	.	.	.	.	.	.	.	.	.	.	.	.	.	.	.	.	.	.	.	.	.	.	C	.	.	.	.	.	.	.	.	.	.	.	.	.	.	.	G	.	.	.	.	.	.	.	.	.	.	.	.	.	.	A	.	.	.	.	.	.	.	.	.	.	.	T	.	.	.	.	.	.	.	.	.	.	.	.	.	.	.	T	.	.	A	.	.	.	.	.	.	.	.	.	.	C	.	T	.	.	.	.	M3
SN20	.	.	.	.	A	.	.	.	.	.	.	.	G	A	.	.	.	.	.	.	.	.	.	.	.	.	.	.	.	.	.	.	.	.	.	.	.	.	.	.	.	.	.	.	G	.	.	.	.	.	.	.	.	.	.	.	.	.	.	A	.	.	.	.	.	.	.	.	.	.	.	T	.	.	.	.	.	.	.	.	.	A	.	A	.	.	.	T	.	.	A	.	.	.	.	.	.	.	.	.	.	.	.	T	.	.	.	.	M5
SN21	.	.	.	C	.	.	.	.	.	.	.	.	.	.	.	.	.	.	.	.	.	.	.	.	.	.	.	.	C	.	.	.	.	.	.	.	.	.	.	.	.	.	.	.	G	.	.	.	.	.	.	.	.	.	.	.	.	.	.	A	.	.	.	.	.	.	.	.	.	.	.	T	.	.	.	.	.	.	.	.	.	.	.	.	.	.	.	T	.	.	A	.	.	.	.	.	.	.	.	.	.	C	.	T	.	.	.	.	M3
